# Massive comparative genomic analysis reveals convergent evolution of specialized bacteria

**DOI:** 10.1186/1745-6150-4-13

**Published:** 2009-04-10

**Authors:** Vicky Merhej, Manuela Royer-Carenzi, Pierre Pontarotti, Didier Raoult

**Affiliations:** 1 Unit for Research on Emergent and Tropical Infectious Diseases (URMITE), CNRS-IRD UMR 6236 IFR48, Faculty of Medicine, University of the Mediterranean, Marseilles, France; 2 Evolutionary biology and modeling, LATP UMR CNRS 6632 FR 3098 IFR 48, University of Provence, Marseilles, France

## Abstract

**Background:**

Genome size and gene content in bacteria are associated with their lifestyles. Obligate intracellular bacteria (i.e., mutualists and parasites) have small genomes that derived from larger free-living bacterial ancestors; however, the different steps of bacterial specialization from free-living to intracellular lifestyle have not been studied comprehensively. The growing number of available sequenced genomes makes it possible to perform a statistical comparative analysis of 317 genomes from bacteria with different lifestyles.

**Results:**

Compared to free-living bacteria, host-dependent bacteria exhibit fewer rRNA genes, more split rRNA operons and fewer transcriptional regulators, linked to slower growth rates. We found a function-dependent and non-random loss of the same 100 orthologous genes in all obligate intracellular bacteria. Thus, we showed that obligate intracellular bacteria from different phyla are converging according to their lifestyle. Their specialization is an irreversible phenomenon characterized by translation modification and massive gene loss, including the loss of transcriptional regulators. Although both mutualists and parasites converge by genome reduction, these obligate intracellular bacteria have lost distinct sets of genes in the context of their specific host associations: mutualists have significantly more genes that enable nutrient provisioning whereas parasites have genes that encode Types II, IV, and VI secretion pathways.

**Conclusion:**

Our findings suggest that gene loss, rather than acquisition of virulence factors, has been a driving force in the adaptation of parasites to eukaryotic cells. This comparative genomic analysis helps to explore the strategies by which obligate intracellular genomes specialize to particular host-associations and contributes to advance our knowledge about the mechanisms of bacterial evolution.

**Reviewers:**

This article was reviewed by Eugene V. Koonin, Nicolas Galtier, and Jeremy Selengut.

## Background

**Figure 1:**
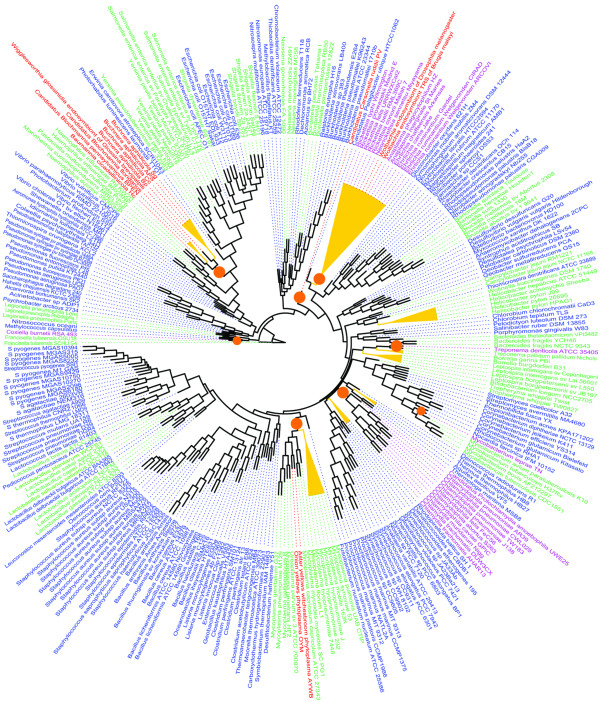
**Phylogenetic relationships and converging evolution**. The phylogenetic unrooted tree was constructed on the basis of the 16S rRNA gene sequences from the 317 bacteria using the neighbour-joining method [15] within the Phylip package [14]. The tree was visualized using FigTree software . Organisms are colored according to lifestyle: red for mutualists, purple for parasitic, green for facultative host-dependent, and blue for free-living bacteria. The events of split rRNA operons (yellow triangles) and loss of the 100 genes set (orange circles) are coincident with the location of the host-dependent bacteria in different phyla.

Genome size in bacteria is extremely variable, ranging from 0.16 megabases (Mb) in *Carsonella ruddii *[1] to approximately 10 Mb in *Burkholderia xenovorans *[2]. Genome size and gene repertoire can increase through gene acquisition, i.e. DNA transfer and gene duplication, and conversely, decrease by deletion [3,4]. Genome analyses of obligate intracellular, including mutualistic and parasitic organisms, showed that these bacteria have small genomes that are derived from larger free-living bacterial ancestors [5-8]. This reductive evolution has been associated with metabolic parasitism or mutualism, during adaptation to an intracellular habitat [7,9,10]. The analysis of gene contents of fully sequenced genomes provided insights into the relationship between the ecology and genome evolution of studied bacteria [11-13]. The growing number of available bacterial genomes makes it possible to perform a comparative genomic analysis of 317 genomes from bacteria with different lifestyles. Bacteria were classified according to their lifestyle as host-dependent (125 bacteria) and free-living (192 bacteria). Among host-dependent bacteria, 85 were identified as facultative host-associated (extracellular or intracellular), and 40 bacteria (27 parasites and 13 mutualists) that are specialized to an obligate intracellular lifestyle (Figure 1 and Additional file 1) [14,15]. We determined the phylogenetic profile of all studied bacteria with respect to their orthologous genes content (COGs). We compared the 317 genomes with respect to their genome size, G+C content, ribosomal RNA operons (rRNA), and orthologous gene composition. We identified genomic features typical of each way of life, highlighting the significant differences in the genomic repertoires between obligate intracellular and free-living bacteria, as well as differences between mutualistic and parasitic bacteria. Our comparative analysis sheds light on the process of evolution from the larger genomes of ancestral species to the specialized smaller genomes of obligate intracellular bacteria, and reveals the genetic basis of their specialization to an intracellular lifestyle. We demonstrate that there is a convergent evolution of obligate intracellular bacteria from different phyla.

## Results and discussion

### Genomic features and lifestyle

**Figure 2:**
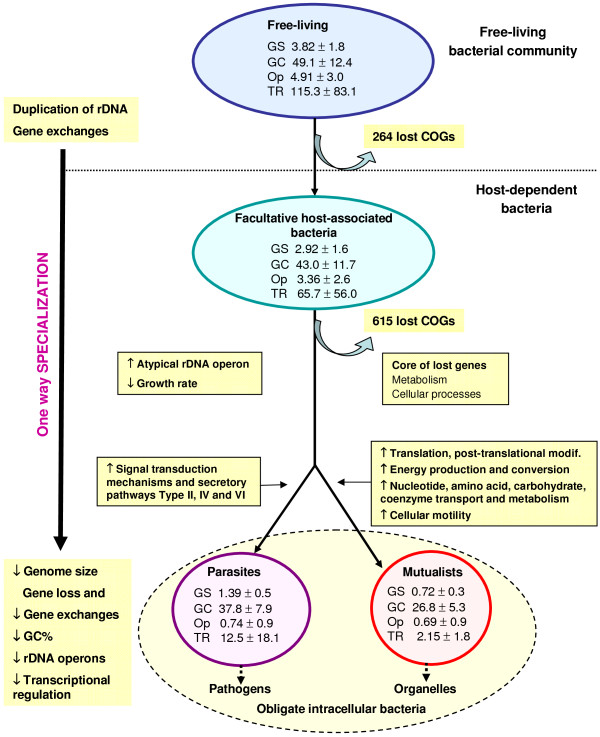
**Proposed scenario for genome evolution and specialization**. Model of evolution involving 3 steps en route to specialization to an intracellular lifestyle. The different steps correspond to the different levels of dependency to eukaryotic cells, the minimum is for the free-living bacteria and the maximum is for obligate intracellular bacteria. Each Step corresponds to a bacterial community sharing common habitat and relationship with eukaryotic cells. Bacterial specialization to an intracellular lifestyle is characterized by gene loss including transcriptional regulators and rRNA operons. Free-living promiscuous bacteria have large genomes because of a high level of gene importation. They also have a large number of rRNA operons. Obligate intracellular bacteria have reduced genomes with few rRNA operons and transcription regulators. When bacterial lineages make the transition from free-living to permanent associations with hosts, they undergo a major loss of genes. Restriction to an intracellular environment limits the opportunity to acquire foreign genes from other bacteria therefore the balance between acquired and lost genes in specialized bacteria is in favour of genome reduction. Irreversible massive gene decay implies that specialization to an intracellular lifestyle is a one-way road. Differential gene loss is noted in mutualistic and parasitic bacterial groups. Data in circles represent the mean (± s.d.) of genome size in megabases (GS), GC content (GC), rRNA operon (Op), and number of genes assigned to transcriptional regulation (TR) in each bacterial community. Numbers on the arrows represent the average number of lost genes ± the standard error in order to compute confidence intervals for the estimated loss ratio (proportion of genes lost with respect to the whole number of genes present at least in one bacterium).

Host-dependent bacteria typically have a smaller genome size and fewer genes compared to their close relatives in the same phylum (Additional file 2). Linear regression analysis showed strong positive correlations between genome size and GC content (*R*^2 ^= 0.376, *F*_1,315 _= 190, *p *< 10^-6^) and between genome size and gene number (*R*^2 ^= 0.976, *F*_1,315 _= 12900, *p *< 10^-6^). Indeed, the trend towards genome reduction holds true for all host-dependent bacteria and the maximum reduction has been noted for the obligate intracellular bacteria (unpaired Student's t-test, all *p *< 10^-2^, Figure 2). The AT mutational bias may be explained by the impairment of the reparation system [16,17] or by metabolic reasons [18,19]. Genome size, number of genes and GC content of bacteria diminish during the specialization to an intracellular lifestyle, indicating a continual selective pressure for a minimal genome [20]. One explanation is the intracellular habitat that limits the capacity for gene acquisition by lateral gene transfer (LGT) [13,21-23]. Other reasons include gene loss with increased adaptation to the host [10,24]. Once restricted to the intracellular environment, the opportunities for LGT are diminished, so the likelihood of reversal is low, and the possibility of ever acquiring the functions needed to live in a less specialized environment successfully (i.e. in competition with better equipped organisms) is small. Indeed, the balance between acquired and lost genes is in favour of genome reduction and irreversible massive gene decay implies that specialization to an intracellular lifestyle is a one-way road.

### Gene repertoire and lifestyle

**Figure 3:**
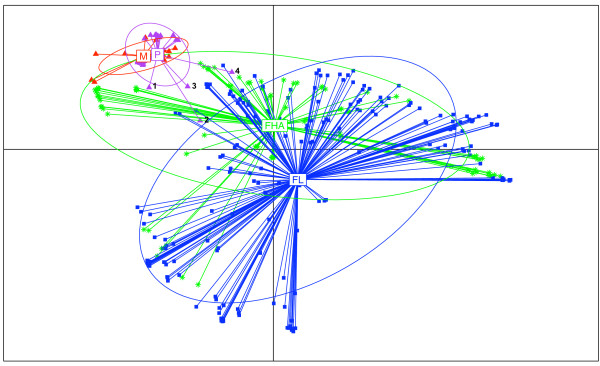
**Plot of the first Principal Coordinate Analysis (PCO) axis of COGs content distances**. Multivariate analysis graphics of the COGs content of all studied bacteria using R *ade4 *package. Each bacterium is represented by a symbol linked by a line to the gravity center of the group it belongs to (M, obligate intracellular mutualists, *red triangles*; P, obligate intracellular parasites, *purple triangles*; FHA, facultative host- associated, *green asterisks*; and FL, free-living, *blue squares*). An ellipse was also drawn for each class, which size increases with the coordinates' dispersion in the class. It is computed such that it would contain 68% of the individuals in the studied class if the distribution were Gaussian. Otherwise, it is just a way to compare dispersion between classes. 1 represents *Treponema pallidum; *2 represents *Mycobacterium leprae; *3 represents Candidatus *Protochlamydia amoebophila *UWE25; 4 represents *Coxiella burnetii*. These species with larger genome sizes and gene contents than the other obligate intracellular bacteria undergo reductive evolution [28,30]. Some of these bacteria have high number of pseudogenes [27,29,31]. The presence of pseudogenes displays an ongoing process of gene degradation.

Using the Reverse PSI-Blast program [25], we were able to assign, on average, 71.03 ± 7.73% of the ORFs (Open Reading Frame) in any genome to a COG functional category. Thus we have characterized the large majority of the repertoire of each bacterium, knowing that ≈ 15 to 20% of the predicted genes in every genome sequenced so far, is species-specific [26]. The principal coordinate (PCO) analysis of COG content distances, calculated on the basis of the presence or absence of a COG, showed that bacteria from the same lifestyle tend to be clustered together (multivariate analysis variance, *p *< 10^-6^, Figure 3) [27-31]. The difference in genome contents reflects a differential gene loss, with a greater extent of loss in obligate intracellular bacteria. To identify function repertoires related to each lifestyle, we compared the number of genes assigned to each COG in the host-dependent genomes with that in free-living bacteria. Genes involved in DNA replication, recombination and repair, RNA processing and modification, translation, post-translational modification, and intracellular trafficking and secretion significantly increased their representation in all the host-dependent compared to free-living bacteria. In contrast, genes belonging to the functional categories of transcription, defence mechanisms, transport and metabolism of amino acids, inorganic ions, and secondary metabolites significantly decreased in their percentage of genome representation (paired Student's t-test, *p *< 10^-2^, Table 1). Bacteria display different functional gene inventories, with functions specific to their ecological niche.

**Table 1:** Distribution of the protein-coding genes of host-dependent and free-living bacteria in COG functional categories

**COG functional categories**	**Code**	**OI **(n = 40)	**FHA **(n = 85)	**HD (n = 125)**	**FL (n = 192)**	**HD vs. FL**
**(a) Gene count Mean +/- s.d**.						*p*-value*

Chromatine structure and dynamics	B	0.28 ± 0.45	0.44 ± 1.07	0.38 ± 0.92	1.16 ± 1.28	0.4982
Replication, recombination and repair	L	56.78 ± 26.49	161.58 ± 171.99	128.04 ± 150.56	141.23 ± 55.84	0.3035
Transcription	K	23.70 ± 10.23	105.65 ± 74.89	79.42 ± 72.84	163.82 ± 99.52	**0.0000**
RNA processing and modification	A	0.70 ± 1.26	1.20 ± 2.09	1.04 ± 1.87	1.11 ± 1.87	0.9290
Translation, ribosomal structure and biogenesis	J	116.68 ± 13.97	143.42 ± 25.75	134.86 ± 25.83	157.62 ± 19.27	**0.0000**
Posttranslational modification, protein turnover, chaperones	O	39.08 ± 10.84	74.60 ± 41.27	63.23 ± 38.31	94.13 ± 39.75	**0.0000**
Intracellular trafficking, secretion, and vesicular transport	U	29.68 ± 10.05	59.62 ± 44.82	50.04 ± 39.86	63.77 ± 44.54	**0.0002**
Signal transduction mechanisms	T	10.48 ± 8.45	56.65 ± 41.04	41.87 ± 40.39	103.67 ± 67.24	**0.0001**
Cell cycle control, cell division, chromosome partitioning	D	11.20 ± 5.52	21.96 ± 9.96	18.52 ± 10.11	25.72 ± 7.73	**0.0000**
Defense mechanisms	V	2.60 ± 4.32	20.56 ± 13.81	14.82 ± 14.35	29.46 ± 14.94	**0.0000**
Cell wall/membrane/envelope biogenesis	M	39.10 ± 22.84	110.27 ± 65.03	87.50 ± 64.34	130.06 ± 59.91	**0.0000**
Cell motility	N	8.23 ± 10.53	44.73 ± 44.12	33.05 ± 40.57	53.24 ± 51.60	**0.0172**
Cytoskeleton	Z	0.08 ± 0.47	0.28 ± 1.09	0.22 ± 0.94	0.24 ± 0.78	0.8927
Nucleotide transport and metabolism	F	26.70 ± 10.47	54.55 ± 19.22	45.64 ± 21.33	69.52 ± 15.52	**0.0000**
Amino acid transport and metabolism	E	39.65 ± 20.39	152.06 ± 108.15	116.09 ± 104.05	213.71 ± 106.02	**0.0000**
Carbohydrate transport and metabolism	G	24.05 ± 10.89	112.52 ± 86.46	84.21 ± 82.57	132.33 ± 70.07	**0.0000**
Lipid transport and metabolism	I	26.15 ± 11.18	58.54 ± 45.94	48.18 ± 41.22	81.96 ± 54.49	**0.0000**
Coenzyme transport and metabolism	H	40.25 ± 17.78	82.91 ± 47.76	69.26 ± 45.21	109.84 ± 39.55	**0.0000**
Inorganic ion transport and metabolism	P	22.48 ± 7.80	89.40 ± 61.77	67.98 ± 59.89	127.67 ± 60.67	**0.0000**
Secondary metabolites biosynthesis, transport and catabolism	Q	6.18 ± 6.09	32.34 ± 40.30	23.97 ± 35.52	50.04 ± 44.50	**0.0000**
Energy production and conversion	C	48.63 ± 16.77	111.98 ± 75.61	91.70 ± 69.58	148.99 ± 82.31	**0.0000**
General function prediction only	R	53.58 ± 27.82	187.75 ± 106.15	144.82 ± 108.75	265.65 ± 119.15	**0.0000**
Function unknown	S	43.23 ± 27.19	184.84 ± 124.19	139.52 ± 122.80	262.59 ± 118.90	**0.0000**
***Total***		***669.43***	***1867.85***	***1484.35***	***2427.53***	

**(b) Percent of genes in the different functional categories (%)**						

Information storage and processing (B+L+K+A+J)		29.60	22.07	23.16	19.15	
Cellular processes and signalling (O+U+T+D+V+M+N+Z)		20.98	20.81	20.83	20.61	
Metabolism (F+E+G+I+H+P+Q+C)		34.97	37.17	36.85	38.48	
Poorly characterized (R+S)		14.46	19.95	19.16	21.76	

OI corresponds to obligate intracellular, FHA corresponds to facultative host-associated, HD corresponds to all host-dependent, FL corresponds to free-living bacteria.

*p-values < 0.05 are shown in bold to indicate significant differences between HD and FL bacteria (paired Student's *t-*test and Wilcoxon signed rank test)

Based upon the assumption that COGs conserved in the reduced genomes of host-dependent bacteria from different phyla are likely to be essential and therefore result from a vertical transmission, we searched for the essential COGs. We found that only 35 COGs were conserved in all 317 bacteria. The size of the essential COGs set could be underestimated due to the small genome size of *Carsonella ruddii *(160 Kb). Indeed, 100 COGs were found conserved in 99% of the bacteria (Table 2). These genes are likely to be essential and are good candidates for inclusion in the minimal gene set. Like other minimal gene sets [32-37], this set consisted mainly of genes encoding for the DNA replication and translation system components, genes that preserve the integrity of their product, such as genes encoding proteins involved in DNA repair, protein degradation and proofreading, chaperone-like proteins, and a few basal components of the transcription system (Table 3, Chi-squared test for independence χ^2 ^= 485.5, *df *= 3, *p *< 10^-6^). Altogether, the essential genes that are conserved in all genomes become proportionally more important in small reduced genomes than in large genomes.

**Table 2:** Set of 100 essential COGs conserved in 99% of bacteria

**COG**	**Code**	**COG's description**
COG0563	F	Adenylate kinase and related kinases
COG0528	F	Uridylate kinase
COG0587	L	DNA polymerase III, alpha subunit
COG2812	L	DNA polymerase III, gamma/tau subunits
COG0592	L	DNA polymerase sliding clamp subunit (PCNA homolog)
COG0358	L	DNA primase (bacterial type)
COG0084	L	Mg-dependent DNase
COG0305	L	Replicative DNA helicase
COG0629	L	Single-stranded DNA-binding protein
COG0188	L	Type IIA topoisomerase (DNA gyrase/topo II, topoisomerase IV), A subunit
COG0187	L	Type IIA topoisomerase (DNA gyrase/topo II, topoisomerase IV), B subunit
COG0202	K	DNA-directed RNA polymerase, alpha subunit/40 kD subunit
COG0086	K	DNA-directed RNA polymerase, beta' subunit/160 kD subunit
COG0568	K	DNA-directed RNA polymerase, sigma subunit (sigma70/sigma32)
COG0571	K	dsRNA-specific ribonuclease
COG0250	K	Transcription antiterminator
COG0195	K	Transcription elongation factor
COG0081	J	Ribosomal protein L1
COG0244	J	Ribosomal protein L10
COG0080	J	Ribosomal protein L11
COG0102	J	Ribosomal protein L13
COG0093	J	Ribosomal protein L14
COG0200	J	Ribosomal protein L15
COG0197	J	Ribosomal protein L16/L10E
COG0203	J	Ribosomal protein L17
COG0256	J	Ribosomal protein L18
COG0335	J	Ribosomal protein L19
COG0090	J	Ribosomal protein L2
COG0292	J	Ribosomal protein L20
COG0091	J	Ribosomal protein L22
COG0089	J	Ribosomal protein L23
COG0198	J	Ribosomal protein L24
COG0211	J	Ribosomal protein L27
COG0087	J	Ribosomal protein L3
COG0254	J	Ribosomal protein L31
COG0088	J	Ribosomal protein L4
COG0094	J	Ribosomal protein L5
COG0097	J	Ribosomal protein L6P/L9E
COG0222	J	Ribosomal protein L7/L12
COG0051	J	Ribosomal protein S10
COG0100	J	Ribosomal protein S11
COG0048	J	Ribosomal protein S12
COG0099	J	Ribosomal protein S13
COG0184	J	Ribosomal protein S15P/S13E
COG0228	J	Ribosomal protein S16
COG0186	J	Ribosomal protein S17
COG0238	J	Ribosomal protein S18
COG0052	J	Ribosomal protein S2
COG0268	J	Ribosomal protein S20
COG0092	J	Ribosomal protein S3
COG0522	J	Ribosomal protein S4 and related proteins
COG0098	J	Ribosomal protein S5
COG0360	J	Ribosomal protein S6
COG0049	J	Ribosomal protein S7
COG0096	J	Ribosomal protein S8
COG0103	J	Ribosomal protein S9
COG0233	J	Ribosome recycling factor
COG0858	J	Ribosome-binding factor A
COG0013	J	Alanyl-tRNA synthetase
COG0018	J	Arginyl-tRNA synthetase
COG0215	J	Cysteinyl-tRNA synthetase
COG0008	J	Glutamyl- and glutaminyl-tRNA synthetases
COG0124	J	Histidyl-tRNA synthetase
COG0060	J	Isoleucyl-tRNA synthetase
COG0495	J	Leucyl-tRNA synthetase
COG0143	J	Methionyl-tRNA synthetase
COG0016	J	Phenylalanyl-tRNA synthetase alpha subunit
COG0072	J	Phenylalanyl-tRNA synthetase beta subunit
COG0193	J	Peptidyl-tRNA hydrolase
COG0442	J	Prolyl-tRNA synthetase
COG0172	J	Seryl-tRNA synthetase
COG0441	J	Threonyl-tRNA synthetase
COG0180	J	Tryptophanyl-tRNA synthetase
COG0162	J	Tyrosyl-tRNA synthetase
COG0024	J	Methionine aminopeptidase
COG0336	J	tRNA-(guanine-N1)-methyltransferase
COG0030	J	Dimethyladenosine transferase (rRNA methylation)
COG0012	J	Predicted GTPase, probable translation factor
COG0216	J	Protein chain release factor A
COG0050	J	GTPases – translation elongation factors
COG0231	J	Translation elongation factor P (EF-P)/translation initiation factor 5A (eIF-5A)
COG0264	J	Translation elongation factor Ts
COG0480	J	Translation elongation factors (GTPases)
COG0361	J	Translation initiation factor 1 (IF-1)
COG0532	J	Translation initiation factor 2 (IF-2; GTPase)
COG0290	J	Translation initiation factor 3 (IF-3)
COG0465	O	ATP-dependent Zn proteases
COG0484	O	DnaJ-class molecular chaperone with C-terminal Zn finger domain
COG0533	O	Metal-dependent proteases with possible chaperone activity
COG0443	O	Molecular chaperone
COG0576	O	Molecular chaperone GrpE (heat shock protein)
COG0691	O	tmRNA-binding protein
COG0653	U	Preprotein translocase subunit SecA (ATPase, RNA helicase)
COG0201	U	Preprotein translocase subunit SecY
COG0706	U	Preprotein translocase subunit YidC
COG0481	M	Membrane GTPase LepA
COG0275	M	Predicted S-adenosylmethionine-dependent methyltransferase involved in cell envelope biogenesis
COG0536	R	Predicted GTPase
COG1160	R	Predicted GTPases
COG0319	R	Predicted metal-dependent hydrolase

**Table 3:** Functional classification of 100 conserved and 100 lost COGs

**COG description**	**Code**	**Conserved**	**Lost**
Chromatine structure and dynamics	**B**	0	0
Replication, recombination and repair	**L**	9	1
Transcription	**K**	6	5*
Rna processing and modification	**A**	0	0
Translation, ribosomal structure and biogenesis	**J**	69	3
**Information storage and processing**		**84**	**9**
Posttranslational modification, protein turnover, chaperones	**O**	6	11*
Intracellular trafficking, secretion, and vesicular transport	**U**	3	0
Signal transduction mechanisms	**T**	0	12*
Cell cycle control, cell division, chromosome partitioning	**D**	0	1
Defense mechanisms	**V**	0	0
Cell wall/membrane/envelope biogenesis	**M**	2	6
Cell motility	**N**	0	8*
**Cellular processes and signaling**		**11**	**38**
Nucleotide transport and metabolism	**F**	2	0
Amino acid transport and metabolism	**E**	0	17*
Carbohydrate transport and metabolism	**G**	0	1*
Lipid transport and metabolism	**I**	0	2*
Coenzyme transport and metabolism	**H**	0	7
Inorganic ion transport and metabolism	**P**	0	8
Secondary metabolites biosynthesis, transport and catabolism	**Q**	0	3*
Energy production and conversion	**C**	0	8
**Metabolism**		**2**	**46**
General function prediction only	**R**	3	10
Function unknown	**S**	0	7
**Poorly characterized**		**3**	**17**

*Some of the COGs in the corresponding functional category belong to other categories as well.

### Convergent reductive evolution of obligate intracellular bacteria

**Figure 4:**
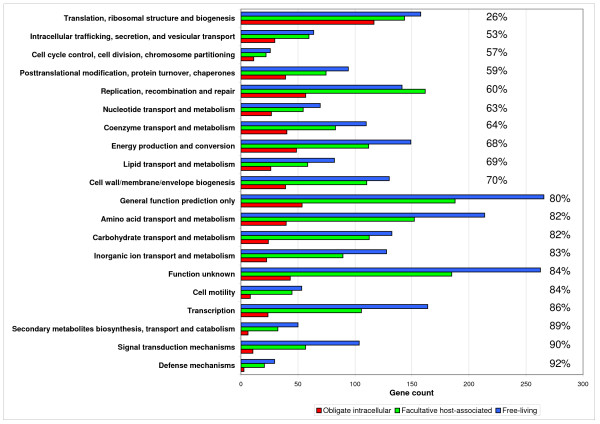
**Functions lost during specialization**. The bars represent the mean number of loci in different functional categories based on functional categorizations established for the clusters of Orthologous Groups (COGs).The proportion of genes lost by obligate intracellular compared to free-living bacteria is indicated next to the bars.

When considering the presence or absence of a COG, we found that facultative host-associated bacteria have 264 COGs less than the free-living bacteria and 615 COGs more than the obligate intracellular bacteria. The differences observed between the different bacterial communities correspond to the COGs lost in relation with the level of host-dependency (mean loss ratio ± sd: 6 ± 11% and 16 ± 17%, Figure 2). Thus, on the first level of dependency (i.e., facultative host-associated lifestyle) bacteria have already lost 264 COGs. To achieve the second level of host-dependency (i.e., obligate intracellular lifestyle) bacteria undergo an additional loss of 615 COGs significantly greater than the previous loss (unpaired Student's t-test, *p *< 10^-6^). Moreover, the number of genes assigned to each COG category decreased in each step of specialization to an intracellular lifestyle (Figure 4). However, we suspected that gene loss was not random and resulted from a converging evolutionary process. To test this hypothesis, we developed a new statistical approach (Details in methods). We considered the most parsimonious hypothesis, suggesting that consistent gene presence in the free-living bacteria in a phylum indicates that the corresponding gene was probably present in the ancestor of that phylum, whereas the occasional absence of a gene in obligate intracellular bacteria might result from gene loss. Also, we considered the repetition of gene loss across distantly related intracellular species in comparison with their close relative free-living bacteria indicative of convergent evolution. By comparing obligate intracellular to their phylogenetically close free-living relatives, in the same phylum, we identified a set of 100 COGs lost in concert by obligate intracellular bacteria from all phyla (Additional files 3 and 4, Table 4). The number of COGs lost in concert was significantly more important than expected (mean loss ratio ± s.e.m = 0.062 ± 7.8 × 10^-3 ^%) if the loss were random (Randomization test, n = 2000, *p *< 10^-6^) (Additional file 5). The set of 100 COGs lost in concert among the obligate intracellular bacteria, mainly encoded metabolism (41%) and cellular process (35%) proteins (Table 3), showing that gene loss is function-dependent (Chi-squared test for independence χ^2 ^= 36.4, degrees of freedom *df *= 3, *p *< 10^-6^). The obligate intracellular bacteria from different phyla did not lose COGs independently (Binomial distribution, *p *< 10^-6^) (Additional file 6). Thereby, using statistical tests, we demonstrated that there is a causal link between specialization and gene loss. The common loss of the same genes in obligate intracellular bacteria from different phyla reflects the convergent evolution of these specialized bacteria in relation to their lifestyle (Figure 1).

**Table 4:** Set of 100 COGs lost by obligate intracellular bacteria

**COG**	**Code**	**COG's description**
COG2264	J	Ribosomal protein L11 methylase
COG1188	J	Ribosome-associated heat shock protein implicated in the recycling of the 50S subunit (S4 paralog)
COG2265	J	SAM-dependent methyltransferases related to tRNA (uracil-5-)-methyltransferase
COG1595*	K	DNA-directed RNA polymerase specialized sigma subunit, sigma24 homolog
COG1508	K	DNA-directed RNA polymerase specialized sigma subunit, sigma54 homolog
COG1522	K	Transcriptional regulators
COG1167	KE	Transcriptional regulators containing a DNA-binding HTH domain and an aminotransferase domain (MocR family) and their eukaryotic orthologs
COG1974*	KT	SOS-response transcriptional repressors (RecA-mediated autopeptidases)
COG1643	L	HrpA-like helicases
COG0277*	C	FAD/FMN-containing dehydrogenases
COG0247*	C	Fe-S oxidoreductase
COG2225*	C	Malate synthase
COG1902	C	NADH:flavin oxidoreductases, Old Yellow Enzyme family
COG0778*	C	Nitroreductase
COG2352*	C	Phosphoenolpyruvate carboxylase
COG1600	C	Uncharacterized Fe-S protein
COG1062*	C	Zn-dependent alcohol dehydrogenases, class III
COG0239	D	Integral membrane protein possibly involved in chromosome condensation
COG0683	E	ABC-type branched-chain amino acid transport systems, periplasmic component
COG1176	E	ABC-type spermidine/putrescine transport system, permease component I
COG1177	E	ABC-type spermidine/putrescine transport system, permease component II
COG2049*	E	Allophanate hydrolase subunit 1
COG1984*	E	Allophanate hydrolase subunit 2
COG2303*	E	Choline dehydrogenase and related flavoproteins
COG0014*	E	Gamma-glutamyl phosphate reductase
COG0405	E	Gamma-glutamyltransferase
COG0263*	E	Glutamate 5-kinase
COG0665	E	Glycine/D-amino acid oxidases (deaminating)
COG0346*	E	Lactoylglutathione lyase and related lyases
COG2755*	E	Lysophospholipase L1 and related esterases
COG1410*	E	Methionine synthase I, cobalamin-binding domain
COG0347	E	Nitrogen regulatory protein PII
COG1280	E	Putative threonine efflux protein
COG2008	E	Threonine aldolase
COG1762	GT	Phosphotransferase system mannitol/fructose-specific IIA domain (Ntr-type)
COG0315	H	Molybdenum cofactor biosynthesis enzyme
COG2896	H	Molybdenum cofactor biosynthesis enzyme
COG0303*	H	Molybdopterin biosynthesis enzyme
COG0521*	H	Molybdopterin biosynthesis enzymes
COG0314	H	Molybdopterin converting factor, large subunit
COG1977	H	Molybdopterin converting factor, small subunit
COG0746	H	Molybdopterin-guanine dinucleotide biosynthesis protein A
COG2267*	I	Lysophospholipase
COG0318	IQ	Acyl-CoA synthetases (AMP-forming)/AMP-acid ligases II
COG2230*	M	Cyclopropane fatty acid synthase and related methyltransferases
COG1596	M	Periplasmic protein involved in polysaccharide export
COG0810	M	Periplasmic protein TonB, links inner and outer membranes
COG1247	M	Sortase and related acyltransferases
COG2148	M	Sugar transferases involved in lipopolysaccharide synthesis
COG3206	M	Uncharacterized protein involved in exopolysaccharide biosynthesis
COG1580	N	Flagellar basal body-associated protein
COG1291	N	Flagellar motor component
COG1344	N	Flagellin and related hook-associated proteins
COG0643	NT	Chemotaxis protein histidine kinase and related kinases
COG2201	NT	Chemotaxis response regulator containing a CheY-like receiver domain and a methylesterase domain
COG0835	NT	Chemotaxis signal transduction protein
COG0840	NT	Methyl-accepting chemotaxis protein
COG1352	NT	Methylase of chemotaxis methyl-accepting proteins
COG0229	O	Conserved domain frequently associated with peptide methionine sulfoxide reductase
COG4235	O	Cytochrome c biogenesis factor
COG1281	O	Disulfide bond chaperones of the HSP33 family
COG0386	O	Glutathione peroxidase
COG2360	O	Leu/Phe-tRNA-protein transferase
COG0652*	O	Peptidyl-prolyl cis-trans isomerase (rotamase) – cyclophilin family
COG1764	O	Predicted redox protein, regulator of disulfide bond formation
COG2518	O	Protein-L-isoaspartate carboxylmethyltransferase
COG3118*	O	Thioredoxin domain-containing protein
COG2844	O	UTP:GlnB (protein PII) uridylyltransferase
COG1391*	OT	Glutamine synthetase adenylyltransferase
COG0725	P	ABC-type molybdate transport system, periplasmic component
COG0600	P	ABC-type nitrate/sulfonate/bicarbonate transport system, permease component
COG0004	P	Ammonia permease
COG1393*	P	Arsenate reductase and related proteins, glutaredoxin family
COG0704*	P	Phosphate uptake regulator
COG0855*	P	Polyphosphate kinase
COG2897*	P	Rhodanese-related sulfurtransferase
COG0659*	P	Sulfate permease and related transporters (MFS superfamily)
COG0179*	Q	2-keto-4-pentenoate hydratase/2-oxohepta-3-ene-1,7-dioic acid hydratase (catechol pathway)
COG3127	Q	Predicted ABC-type transport system involved in lysophospholipase L1 biosynthesis, permease component
COG0664*	T	cAMP-binding proteins – catabolite gene activator and regulatory subunit of cAMP-dependent protein kinases
COG5001*	T	Predicted signal transduction protein containing a membrane domain, an EAL and a GGDEF domain
COG0394	T	Protein-tyrosine-phosphatase
COG3852	T	Signal transduction histidine kinase, nitrogen specific
COG1253	R	Hemolysins and related proteins containing CBS domains
COG0714*	R	MoxR-like ATPases
COG1741	R	Pirin-related protein
COG0388*	R	Predicted amidohydrolase
COG2081	R	Predicted flavoproteins
COG1694	R	Predicted pyrophosphatase
COG1611*	R	Predicted Rossmann fold nucleotide-binding protein
COG0523	R	Putative GTPases (G3E family)
COG2334	R	Putative homoserine kinase type II (protein kinase fold)
COG1540*	R	Uncharacterized proteins, homologs of lactam utilization protein B
COG0397	S	Uncharacterized conserved protein
COG1576	S	Uncharacterized conserved protein
COG2127*	S	Uncharacterized conserved protein
COG2606*	S	Uncharacterized conserved protein
COG2983	S	Uncharacterized conserved protein
COG1671	S	Uncharacterized protein conserved in bacteria
COG3024	S	Uncharacterized protein conserved in bacteria

*indicates COGs that are present in the genome of *Mycobacterium leprae*

### Correlation between transcription, translation and growth rate

The DNA encoding ribosomal RNAs (rRNA) genes of bacteria are typically organized in operons with the general structure 16S-23S-5S, transfer RNA (tRNA) genes are often found in the spacer between the 16S and the 23S rRNA genes [38]. Host-dependent bacteria have fewer copies of each rRNA gene than free-living and significantly lower copy number of typical rRNA operon (2.52 ± 2.53 vs. 4.91 ± 2.98 copies, *p *< 10^-6^). This difference in the number of typical rRNA operons between host-dependent and free-living bacteria remained significant when adjusted for genome size (1.07 ± 1.06 vs. 1.46 ± 0.97 operons/Mb, *p *= 0.001). Atypical operons, where the general structure 16S-23S-5S is disrupted by protein-coding genes, were found significantly more frequently in host-dependent than in free-living bacteria (38 vs. 5, Chi-squared test for independence χ^2 ^= 49.9, *df *= 1, *p *< 10^-6^, these bacteria are listed in Additional file 1). Our data show that different independent clades of host-dependent bacteria contain few copies of ribosomal RNA genes which do not form an operon (Figure 1). The split in rRNA operon is a key evolutionary factor for obligate intracellular bacteria from the order *Rickettsiales *[39,40]. It has been suggested that recombination between gene repeats might have led to both gene loss and genome shuffling in *Rickettsia and Wolbachia spp*. [41,42]. The split in rRNA operon helps to elucidate one of the mechanisms of specialized intracellular genomes evolution.

**Figure 5:**
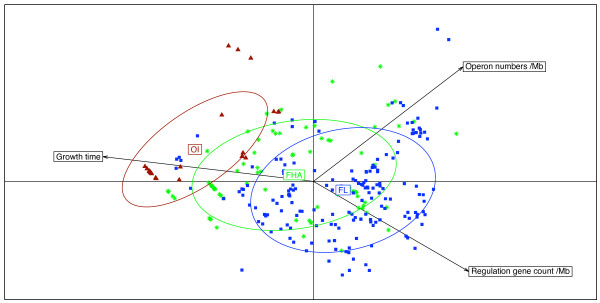
**Relationship between growth time, operon number and transcriptional regulators per Mb**. Bacteria were classified into 3 categories depending on the experimental growth time: fast growing (24–48 hours), median (3 to 7 days) and slow growing fastidious bacteria (more than 7 days). The 284 genomes for which information about time of growth is available are projected on the first two Principal Component Analysis (PCA) axes, which represent 66.2% and 19.5% of the total inertia. OI, obligate intracellular bacteria, *dark red triangles*; FHA, facultative host-associated, *green asterisks*; FL, free-living, *blue squares*.

When counting the number of genes involved in transcription, host-dependent bacteria were found to have significantly fewer transcriptional regulators. This decrease is pronounced in obligate intracellular bacteria (6.98 ± 12.32 genes/Mb) compared to facultative host-associated (19.07 ± 11.49 genes/Mb, *p *< 10^-6^) and to free-living bacteria (28.69 ± 11.18 genes/Mb, *p *< 10^-6^). The ratio of genes involved in transcriptional regulation over the total number of genes involved in transcription in free-living (66.3 ± 10.7%) is significantly greater than that of facultative host-associated (52.46 ± 17.5%) and than that of obligate intracellular bacteria (26.11 ± 14.8%) (unpaired Student's t-test, both *p *< 10^-6^). These genomic features dealing with transcription and translation (the rRNA apparatus) may have an implication in a phenotypic criterion such as growth time. When compared to free-living bacteria, obligate intracellular bacteria have lower copy numbers of the rRNA genes, increased rearranged rRNA operon structures, and fewer transcriptional regulators, and a tendency of slow growth (Figure 5). Moreover, we found a significant negative correlation between growth time on one side and the number of rRNA operons and transcriptional regulators per Mb on the other side (*F*_2,284 _= 93.7; adjusted *R*^2 ^= 0.393; *p *< 10^-6^, Additional file 1). These findings are all correlated with the obligate intracellular bacterial lifestyle and make sense with respect to the physical constraints of that lifestyle. Free-living bacteria exhibit larger genomes, more lateral gene transfer [13], and more rRNA operons. They have great capabilities to adapt to different environmental surroundings, like soil and water. A high copy number of rRNA may be necessary to tolerate increased gene content and larger genome size. The occurrence of multiple typical operons may be important in the ability of bacteria to respond to changing growth conditions [43]. Moreover, the versatile environments of free-living organisms require greater regulatory potential than do the relatively stable niches of obligate intracellular bacteria [4,44]. Specialization is correlated with a lower possibility of gene exchanges with other bacteria [13] add to this a more constant environment that may explain the lack of positive selection for both rRNA operon copies and transcriptional regulators [24]. Finally, the decrease of host-dependent bacteria's growth rate may be critical to synchronize with that of their host cells in order to avoid detrimental virulence (cell lysis).

### Divergence of parasites and mutualists

**Figure 6:**
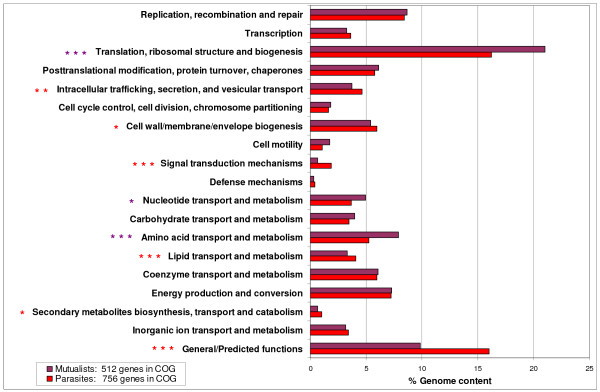
**Comparison of the genome content from mutualistic and parasitic bacteria**. Bars correspond to the mean number of genes in a given COG divided by the total number of genes. The significance of results in the figure is represented by triple asterisks (***) indicating *p *≤ 0.001, double asterisks (**) indicating *p *≤ 0.01 and a single asterisk (*) indicating *p *≤ 0.05 (paired Student's *t-*test).

Among obligate intracellular bacteria, we observed differences between the genomic repertoires of mutualists and parasites that seem to reflect the nature of the relationship these organisms have with their host-cells (beneficial and harmful, respectively). Mutualists have smaller genomes than parasites (0.72 ± 0.27 vs. 1.39 ± 0.49 Mb, Wilcoxon rank sum test, *p *< 10^-6^) and they have significantly less genes in almost all COGs categories. Nevertheless, the genome content difference between mutualists and parasites is not significant for genes involved in amino acid transport and metabolism (40.62 ± 21.21 vs. 39.19 ± 20.38), nucleotide transport and metabolism (25.38 ± 11.30 vs. 27.33 ± 10.20), cell motility (8.85 ± 12.66 vs. 7.93 ± 9.59), and cell cycle control, cell division, and chromosome partition (9.38 ± 5.09 vs. 12.07 ± 5.59). These findings show that despite genome reduction, mutualists have retained genes involved in the transport and metabolism of amino acid and nucleotide, genes for cell motility and cell cycle control. Moreover, 8.76% of the genome vs. only 5.18% (paired Student's t-test, *p *< 10^-4^) is occupied by genes for amino acid transport and metabolism (Figure 6). Parasitic reduced genomes have eliminated genes underlying biosynthesis of amino acids that can be obtained from the host cytoplasm [45], whereas mutualistic genomes have retained genes for the biosynthesis of amino acids required by their hosts [46]. Likewise, mutualists devote a higher fraction of their genomes than parasites for genes involved in nucleotide transport and metabolism (4.7% vs. 3.77%) and genes for translation, ribosomal structure and biogenesis (22.92 vs. 16.74%) (paired Student's t-test, all *p *< 0.05). In contrast, mutualists devote a lower fraction of their genomes than parasites for genes involved in lipid transport and metabolism (2.98 vs. 4.13%), secondary metabolites biosynthesis transport and metabolism (0.62 vs. 0.95%), in cell wall, membrane and envelope (4.76 vs. 5.80%) signal transduction mechanisms (0.61 vs. 1.78%) and intracellular trafficking and signaling (3.47 vs. 4.84%) (paired Student's t-test, all *p *< 0.05) (Figure 6). Some genes increased their representation per genome size in the smaller genomes of mutualists compared to parasitic genomes, such as genes encoding for proteins involved in translation (169.54 ± 56.24 vs. 92.64 ± 18.99 genes/Mb); post-translational modification, protein turnover, and chaperones (47.13 ± 15.57 vs. 32.22 ± 6.98 genes/Mb); cell motility (14.11 ± 19.26 vs. 6.43 ± 8.49 genes/Mb); energy production and conversion (55.26 ± 19.04 vs. 40.38 ± 10.50 genes/Mb); and the transport and metabolism of nucleotides (34.70 ± 12.67 vs. 20.21 ± 6.89 genes/Mb), amino acids (69.56 ± 46.10 vs. 27.91 ± 7.64 genes/Mb), carbohydrates (31.07 ± 13.32 vs. 19.09 ± 7.78 genes/Mb), and coenzymes (43.10 ± 28.66 vs. 33.17 ± 9.73 genes/Mb, paired Student's t-test, all *p *< 0.05). In contrast, parasites have significantly more genes/Mb involved in signal transduction mechanisms (10.02 ± 5.25 vs. 4.68 ± 2.35, paired Student's t-test, *p *= 0.011) that may facilitate the process of entry and survival in cells; as parasitic life requires passage outside the host cell to allow horizontal transmission [47]. Parasites exhibit a very specialized repertoire of secretory pathway genes, type II, IV, and VI (Wilcoxon rank sum test, *p *< 0.05, Additional file 7), enabling them to modulate the host environment by secreted effector molecules [48]. This divergence between parasites' and mutualists' genomes is striking because intracellular parasites have been previously considered as a possible intermediate step to a mutualist lifestyle, en route to the extreme situation of becoming organelles (mitochondria and chloroplasts). Our comparative genomics of parasites and mutualists point out to general similarities and distinctions in the evolution of bacteria specialized to intracellular lifestyle. Obligate intracellular genomes have undergone a reductive evolution, however they have evolved different strategies for bacteria-host interactions and they have lost and conserved genes accordingly [49]. Finally, transitions between parasitism and mutualism might be restricted, owing to the irreversible loss of genes and the associated functional capabilities.

## Conclusion

Based on the comparative genomics of a large number of bacterial genomes, we postulate that bacterial specialization is a one-way irreversible and converging journey causing massive gene loss. New specialists are constantly provided by free-living bacterial community reservoir. In contrast to what was initially hypothesized, the evolution of pathogenic bacteria, that are specialized bacteria of particular interest to humans [50], consists mainly of gene losses [10,51,52]. This general evolution strategy was recently confirmed for *Rickettsia *species (unpublished data), *Mycobacterium tuberculosis *[53,54] and *Mycobacterium ulcerans *[55]; free-living bacteria having more genes named virulence factors than do pathogenic bacteria [13]. It is noteworthy that virulence factors, like genes encoding for the ATP/ADP translocases have been identified in *Chlamydiae *and *Rickettsiae *[56,57]. The presence of these transport systems in obligate intracellular parasites' genomes helps to exploit the host's ATP [9]. It has been suggested that these transport systems have been transferred horizontally to obligate intracellular bacteria during their specialization to an intracellular parasitic lifestyle [58]. Lateral gene transfer seems to be a very rare event in the evolution of intracellular bacteria, comparing to the gene loss events [13]. Increase in virulence may, therefore, be related to the loss of regulation rather than to gene acquisition [52].

## Methods

### Genome data

A total of 317 bacterial genomes were obtained from the NCBI website  together with their genome size, GC content, and the number of genes. We classified bacterial species on the basis of their ecological diversity: host-dependent bacteria living in association with eukaryotic cells and free-living bacteria living in soil, water, or in the air, independently of a eukaryotic cell according to the information given in literature. Host-dependent bacteria were divided into two subgroups: facultative host-associated and obligate intracellular. Obligate intracellular bacteria were classified as mutualists or parasites, depending on the effect of the association on the fitness of the host: positive or negative, respectively (The entire list of studied bacteria along with their genome features can be found in Additional file 1). In this paper, the term "specialists" refers to obligate intracellular bacteria specialized to an intracellular lifestyle.

### Identification of orthologs

We retrieved protein sequence data for bacterial genomes from the KEGG database [59] and COG data from NCBI [60,61]. In the COG database, individual COGs are clustered into 23 functional categories, which are further grouped into four major classes: information storage and processing, cellular processes and signaling, metabolism and poorly characterized. Each set of all predicted proteins was compared to the COG profile database using the Reverse PSI-Blast program [25]. A significance score was determined for each COG so that any sequence not used to build the COG profile scored below this score. ORFs were assigned to a functional category according to the category where their best COG homolog is classified.

In the COG database constructed herein, the presence or absence of a COG in a given organism was noted as 1 or 0, respectively [62]. On the basis of this matrix [0,1] we performed a principal coordinate (PCO) analysis to suggest relationships between genomes [63]. For that we used the resulting pattern of 1's and 0's for the presence or absence of a COG to construct the matrix for Euclidean distances between pairs of points. Then we performed a projection to get a 2-dimensional visualization.

We determined the mean number of genes assigned to each COGs for each bacterial lifestyle. We compared the mean number of genes assigned to each COG function between free-living bacteria (125 organisms) and all host-dependent bacteria (125 organisms), then between free-living and obligate intracellular bacteria (40 organisms), and between mutualists (13 organisms) and parasites (27 organisms). All data were analyzed with the R statistical software package [64] using the Student's *t-*test (either paired or unpaired) for sample sizes of 30 or more per group and Wilcoxon signed rank test or Wilcoxon rank sum test for small samples (paired or independent samples, respectively). All tests were two-tailed, and *p*-values < 0.05 were considered significant.

### Determination of lost COGs

We used the alpha- and gamma-proteobacteria species as a model system for our comparative studies because the variation of the genome size in these subdivision spans the entire size range for bacteria from 0.86 Mb in *Neorickettsia sennetsu *to 5.51 Mb in *Rhodopseudomonas palustris *and 0.16 Mb for *Carsonella ruddii *to 7.22 Mb in *Hahella chejuensis*. Furthermore, there is a large variation in lifestyles in these subdivisions, including obligate intracellular (*Rickettsia *and *Wolbachia*; *Coxiella *and *Buchnera*), facultative host-associated (*Bartonella *and *Brucella*, *Legionella*, *Haemophilus*) and free-living (*Zymonas mobilis *and *Pseudomonas sp*.) bacteria, which enables correlations between gene content and lifestyle features to be examined. We looked for significantly lost COGs: as the mean number of genes in free-living bacteria is three times that of obligate intracellular bacteria, we looked for COGs present in more than 75% of free-living bacteria, but fewer than 25% of obligate intracellular bacteria in alpha- and gamma-proteobacterial phyla (Additional file 3). We further searched for these COGs (presence or absence) within the genomes of other obligate intracellular bacteria belonging to distinct phyla. The 100 COGs lost in concert among the obligate intracellular bacteria from all phyla constitute the core of lost COGs.

### Gene function and evolutionary relatedness

Under the null hypothesis that loss or conservation of COGs is due to chance, independently of their function, the classification of the COGs lost in concert into the different functional categories should be the same as that of all COGs present in bacteria. We compared the functional classifications of both lost and conserved COGs, to that of all COGs which are present in at least one bacterium, using Chi-squared test for independence χ^2^. We also looked for functions that are significantly more lost or conserved, than expected.

### Test of convergent evolution

We evaluated the probability that a COG is in the core of lost COGs given that it has been lost at least by mutualists or parasites from alpha- or gamma-proteobacteria (Additional file 4). We used a randomization test to see if the observed numbers of losses in common can be attributed to random chance (Additional file 5). For that, we have simulated 2000 random losses for mutualists and parasites from alpha- and gamma-proteobacteria, among the COGs present at least in one of these bacteria. Then we counted the number of COGs lost in common and computed the proportion that it represents from the set of COGs lost by at least one bacterium. We summarized the 2000 simulated proportions in a histogram, where we add the 2.5% and 97.5% quantiles. Under random loss assumption, common loss proportions should not exceed these interval bounds.

Furthermore, under the null hypothesis that all observed convergent losses can be explained by random chance, the common loss probability p_1 _(0.0259) should be equal to the product



Where r_θ _= probability that the COG is lost by bacteria of the group θ given that it is at least present in free-livings from α – or γ-proteobacteria (set of N = 3865 COGs).

We computed the theoretical probability p_0 _for any COG to be in the set of COGs lost in concert and we used the binomial distribution B (N, p) to compute the probability of observing, under the hypothesis of independent phyla, at least as many convergent losses. The random variable counting COGs lost in concert among the N COGs, under the hypothesis of independent phyla, has a Binomial distribution B (N, p_0_). We computed P (X ≥ 100), that indicates whether independence hypothesis is likely or not. Although, the real theoretical probability p_0 _is not given yet it can be estimated from loss proportion in each phylum. Moreover we know the behaviour of P(X ≥ 100) with respect to probability p_0. _It increases with p_0 _and reaches the critical probability 0.05 when p_0 _becomes greater than 0.0218 (Additional file 6). Consequently, if probability p_0 _is contained, with sufficiently confidence, in an interval with upper bound less than 0.0218, then convergence is proved. Hence we use observed losses counts to give a confidence interval for each phylum loss probability, from which we deduced a confidence interval for the unknown probability p_0_. Results are given using the 95%-confidence interval of p_0_.

### Ribosomal DNA database

We created a ribosomal RNA (rRNA) and transfer RNA (tRNA) database using BLASTn (e-value threshold of 10^-4^) for the 16S, 23S, 5S rRNA, and tRNA gene sequences from NCBI Genbank against the complete genome sequences of studied bacteria. For each ribosomal operon, the base-pair coordinates corresponding to the 3' terminus of the 16S rRNA and the 5' start of the 23S rRNA gene were entered into the sequence retrieval function on the created database and used to extract the Internal Transcribed Spacer (ITS) sequence. We determined the number and organization of rRNA operons, the ITS length, and the number of tRNAs (Additional file 1).

### Transcription, intracellular trafficking and secretory pathways

We counted the number of genes for the COGs involved in basal transcription (90 COGs) and transcriptional regulators (111 COGs) in all studied bacteria (Additional file 8). We determined the ratio of transcriptional regulators, i.e., the proportion of genes that have a function as transcriptional regulators, over the total of genes involved in transcription. We compared the number of genes involved in transcriptional regulation and the ratio of transcriptional regulators between obligate intracellular, facultative host-associated and free-living bacteria using unpaired Student's t-test. We counted the number of genes assigned to the intracellular trafficking and secretory pathways (Type II, III, IV, V and VI) in all obligate intracellular bacteria (104 COGs including a list of COGs that are involved in the sixth secretory pathway [65], which is not currently described in the COGs' database) (Additional file 9). Data were analyzed using Wilcoxon rank sum test.

## Competing interests

The authors declare that they have no competing interests.

## Authors' contributions

PP and DR designed the research project. VM performed the data collection and analysis. MRC provided statistical and analytical support. VM, PP, and DR analyzed the data. VM and DR wrote the paper. All authors read and approved the final version.

## Reviewers' comments

### Reviewer's report 1

Eugene Koonin (National Center for Biotechnology Information, NIH)

This paper reports a massive comparative-genomic analysis of parasitic and symbiotic bacteria and comes to the conclusion that their evolution is dominated, at least, quantitatively, by extensive gene loss that proceeds, in large part, along parallel routes in distant organisms. Special emphasis is made on the loss of regulatory genes that is considered to be a potentially more important process leading to pathogenicity than acquisition of "virulence factors".

I believe that the conclusions of the paper are, basically, correct. Just a few comments, not so much to criticize, but rather to put these conclusions into the context of previous research and thinking.

1. The parallel loss of genes in diverse bacterial parasites and symbionts, certainly, has been noticed before, for instance, in the context of the analysis of genome-trees constructed by gene content comparison in which the parasitic and symbiotic forms tend to cluster together (Wolf et al. Trends Genet. 2002 Sep;18(9):472–9.)

2. The paths of gene loss are partially parallel and partially divergent as also noticed on many previous occasions, just for instance: Foster J. PLoS Biol. 2005 Apr;3(4):e121

3. Parallel gene loss seems to be quantitatively dominant in the evolution of parasites and symbionts, but this is not to discount "virulence factors" (generally viewed) that can be qualitatively critical. Even one such gene can make a big difference like, for instance, ATP/ADP translocase in Rickettsia and Chlamydia.

**Authors' response**: The authors thank the reviewer for these comments. We have referred to these works in the paper.

### Reviewer's report 2

Nicolas Galtier (CNRS-Université Montpellier II)

This manuscript provides a thorough analysis of >300 bacterial genomes, distinguishing free-living from (various levels of) host-dependent species. It is reported that parasitic and mutualistic bacteria have a higher AT-content, a lower number of ribosomal RNA genes and intact ribosomal operons, and have experienced massive gene loss in a non-independent way – host-dependent species from distinct phyla tend to lose/retain the same genes and functions.

I found the analysis well-conducted, and the results interesting. Not everything is new, of course, but this is the first overall picture of the situation, as far as I know, and I learnt much by reading this piece. Here are a couple of comments.

The discussion about rRNA operon, growth rate and lifestyle appears a bit unclear to me. The relationship between doubling time and number of rRNA operons seems pretty strong (but unfortunately not shown, figure 5 being difficult to decipher). My guess would be that growth rate, which everybody says is primarily limited by protein synthesis, is the leading player here. So the question could be: why would intracellular species evolve a slower growth rate than free-living bacteria? The authors invoke environmental stability and reduced number of genes, but the connection is not obvious. I would suggest that growth rate is under more subtle selective pressure in a host-dependent species than in a free-living species. This is because the growth rate of a parasite/mutualist is (presumably) strongly related to its virulence. Because of the two-level selection process – between individuals within a host vs. between colonies of distinct hosts – growth rate in host-dependent bacteria could be limited to avoid too strong, detrimental virulence.

**Authors' response**: Intracellular genomes encounter elevated genetic drift resulting from relaxed selection on many genes and from radical change in population structure that results in lowered efficacy of selection on genes [66-68]. It has been suggested that the occurrence of multiple typical operons may be important in the ability of bacteria to respond to changing growth conditions [43]. Living continuously within the host eliminates the extreme environmental fluctuations encountered by free-living bacteria [24]. Selective coefficients for the maintenance of genes for regulation and for rRNA genes may be reduced in an intracellular environment, resulting in the loss of these genes.

We found a significant negative correlation between generation time on one side and the number of rRNA operons and transcriptional regulators on the other side. Growth rate is linked on one hand to the number of rRNA operons which are the principle apparatus for translation and to the regulation of transcription on the other hand. Hence, as you said growth rate is primarily limited by protein synthesis. The slower growth rate of intracellular bacterial parasites may be important as these bacteria have to dissimulate in order to avoid immune system. Add to this growth leads to host-cell lysis in *Chlamydia *for example, thus to the loss of the host supply. Therefore, slow growth for parasitic bacteria may be essential to prolong their life within cell. The slow growth rate is also beneficial for mutualistic bacteria. Mutualists and their host exchange gene products, because of this complementary and mutual dependence, mutualistic bacteria have to synchronize their metabolism with that of their host. Indeed, symbiotic relationship implicates that the endosymbionts and the host cell live in concert. Altogether, slow growth may be under more selective pressure in host-dependent bacteria than in free-living bacteria.

**Reviewer**: Reporting that parasitic and mutualistic bacterial species have distinct, irreversible gene-loss signatures, the authors question the scenario invoking parasitism as an intermediate step before mutualism, which is a good point. They do not, however, comment on the fact that "global" markers of host-dependence (AT-content, number of genes, number of rRNA operons) are more extreme in mutualists than in parasites. I would tend to interpret this as a consequence of higher stability in time of mutualistic associations, as compared to parasitism, which seems to make sense. This is good self-corroboration for the life style annotations used in this study.

**Authors' response**: Thank you for this remark. We have demonstrated that there is a significant difference in genome content between parasites and mutualists due to the differential gene loss in relation with their lifestyles. Add to this the sequestration of these intracellular genomes limits their capacity for lateral gene transfer, which renders gene loss irreversible. Altogether, these observations suggest that mutualism and parasitism are two distinct host-relationships supported by significantly different sets of functions. The difference between mutualists and parasites is significant for many functions, especially for amino acids transport and metabolism that represent a higher fraction of the mutualistic genomes than in the parasitic genomes.

Relevant genomic studies about mutualists, like Buchnera spp., revealed the stability of these genomes [21]. This genomic stasis (absence of chromosome rearrangements and gene acquisition) is likely attributable to the loss of phages, repeated sequences, and recA. Hence, the loss of genetic elements that mediate recombination is responsible for the genome stability. In contrast, genome analyses of parasitic genomes like Rickettsiae, showed gene rearrangements [39] and genome shuffling [69]. Moreover certain genomes contain plasmids and present evidence for lateral gene transfer [70,71]. Even though these events are rare in obligate intracellular bacteria comparing to free-living bacteria, they are more important in parasitic genomes than in symbiotic genomes. Altogether, these observations confirm that mutualistic genomes present a higher level of genome stability than parasitic genomes.

**Reviewer**: By the way: where do these annotations come from? Is there a database for bacterial ecology?

**Authors' response**: Considering the large amount of bacterial genomes, we were interested in a possible relationship between the phenotype and the genome. We doubt there is a database for bacterial ecology. We made an exhaustive literature review looking for information about bacterial lifestyle. We checked for the habitat of these bacteria and eventual eukaryotic cell-dependency. Bacterial classification is indicated in additional file 1.

**Reviewer**: One major result of the paper is the discovery of non-independent gene loss in various, distantly related, transitions from free-living to host-dependent life style. This result, however, is perahps not surprising, knowing that essentiality varies across genes – some can be lost, some can not, irrespective of life style. The authors partly account for this by restricting the statistical test to the set of genes lost at least once, so that essential genes are not considered in this analysis. Even among "losable" genes, however, the probability of gene loss irrespective of life style could vary. I would suggest to perform a control analysis in which host-dependent genomes would be replaced by small, free-living genomes – several free-living alpha and gamma proteobacteria have less than 2500 genes. It could be good to know what is specific to host-dependence-associated genome reduction, as compared to "random" (whatever it means) genome reduction.

**Authors' response**: The process of genome reduction and gene loss is well-known for obligate intracellular bacteria [8,10,20,72]. The aim of this paper was not to reproduce such results but to test for an eventual convergent evolution, characterized by a non-random loss induced by the common phenotype.

According to your suggestion, we have performed the control analysis to verify that the 100 lost COGs are specific to the reductive evolution of obligate intracellular bacteria. For that we have separated free-living bacteria into two groups: free-living small genomes and free-living large genomes using the cut-off of 2.92 Mb which is the mean genome size of facultative host-associated bacteria. In order to make the comparison between phylogenetically close relatives, we have treated the only phyla where there were small and large genomes. Genome sizes are given in additional file 1. We got 8 phyla *Alpha*-, *Beta*- and *Gammaproteobacteria*, *Clostridia*, *Lactobacillales*, *Bacillales*, *Actinobacteria*, *and Cyanobacteria*, which represent a total of 168 free-living to include in the analysis. In each phylogenetic group, we identified COGs that are lost by 75% of the small free-living and conserved in more than 25% of the large free-living. First, we looked for COGs that are lost in common, i.e. by small free-living from more than one phylum. Second, in order to see if the 100 lost COGs are specific to the obligate intracellular reductive evolution, we studied the losses of small free-living bacteria. Thus, we compared their loss distribution for a COG in the set of the 100 COGs to the loss distribution for a COG in all the other COGs (among COGs lost at least once in small free-living), using a Chi-squared test for independence.

We found no COG lost in common by all small free-living from the 8 phylogenetic studied groups. Similarly, we didn't find a COG that is lost in concert by small free-living from 7 or 6 groups. Thus no convergence phenomenon occurs within these phylogenetic groups, and the hypothesis of independent loss for small free-living cannot be rejected. The majority of the losable COGs are lost by only one phylogenetic group (Additional file 10). Free-living small genomes lose COGs from the set of 100 COGs or from the set of other COGs without difference (Chi-squared test for independence χ^2 ^= 3.695, df = 2, p-value = 0.158). Thus, in the free-living loss process, there is no significant preference for the set of 100 COGs that we found associated to the obligate intracellular genome's reduction. Consequently, we can deduce that these 100 COGs are specific to the reductive evolution of obligate intracellular genomes.

### Reviewer's report 3

Jeremy Selengut (The Institute for Genomic Research)

This work presents a useful overall comparative analysis and comparison of the genomic content of free-living, host-associated and obligate intracellular organisms. The well-known observation that small genome sizes are correlated with host-association is supported with concrete data, but more importantly, this is dissected with respect do different types of host-associated lifestyle. Difference between mutualists and parasites are delineated, and most importantly, commonalities are found between the classes of genes and changes in rRNA operons that are observed in many phylogenetically independent cases of adaptation to a host-associated lifestyle.

Unfortunately, in its current form, this manuscript suffers from many faults in language, logic, organization, data analysis and data presentation. I have offered extensive comments on these issues and sincerely hope that these deficiencies can be improved. None of these issues is fatal, and I expect that with an improved manuscript I will be able to provide a more positive endorsement in the public commentary accompanying its publication.

Authors' response: Thank you for the time you have spent revising the manuscript. Thank you for all the valuable comments you have addressed to us. Please find attached the manuscript revised according to your remarks. We have made the corrections in language and data presentation. We tried to clarify some areas of the method and data analysis. Hope that this version of the manuscript conforms to the requirements and can get your positive support.

#### Final Review

**Reviewer**: The authors, in their attempt to probe into the nature of genome reduction in obligate intracellular (OI) "specialists", lay out a stepwise model from large free-living organisms to somewhat reduced host-associated organisms finally to OI organisms with their small genomes. On the one hand, this model is inarguable, in order to go from large to small, an intermediate medium state must be passed through. A pertinent question, however, is whether currently observable host-associated, moderately reduced species are representative in terms of their detailed gene content of that intermediate state which OI organisms passed through. Are the host-associated organisms of today the OI organisms of the future, or did today's OI organisms pass through a different kind of intermediate state on their evolutionary pathway?

**Authors' response**: The purpose of this paper was to correlate genomic features with phenotype i.e. the small genome size and gene content with the bacterial lifestyle. For that we classified bacteria in 3 large communities on the basis of their lifestyles then we compared their genomic contents. According to previous relevant papers, small genome is not an ancestral state. Molecular phylogenetic studies and genome analyses showed that the small reduced genomes derived from large genomes through massive gene loss [5-8,20,73,74]. Our analysis of genome content shows that facultative host-associated bacteria constitute a large community of bacteria that we can consider as intermediate because their genome content is smaller than that of free-living bacteria and larger than that of obligate intracellular bacteria (Figure 3). Moreover, genome analysis of obligate intracellular bacteria of *Rickettsia *spp. revealed the presence of degraded genes and gene remnants [41]. The presence of gene remnants in these reduced genomes constitutes molecular fossils that witnesses for the gradual genome degradation leading to specialized intracellular bacteria, and confirms the role of intermediate stage that we can attribute to the facultative host-associated bacteria. In fact, our results confirm the simple evidence that the more genes are lost the more a bacterium becomes dependent of its host.

**Reviewer**: A separate question to be addressed is whether all OI organisms, irrespective of the specifics of the evolutionary paths they have travelled to get there, have more or less arrived at the same end point. There are two ways to look at this question, one could try to determine whether the set of remaining genes is the same across all OI organisms, or one could ask whether the set of genes *lost *from free-living ancestors is similar. The former method is simpler, more commonly essayed, but the answers it provides are much clouded by the lineage-specific genes which are what make each OI organism unique and adapted to its particular host. The latter tack is the focus of this work and requires a bit more effort to reconstruct an accounting of the lost genes.

**Authors' response**: Our analysis considers the lost and the remained genes. One, we could demonstrate that the loss event concerns the same set of COGs and that is what we described as convergent reductive evolution. Second, we compared the gene content between obligate intracellular bacteria, parasites vs. mutualists, and we showed that gene inventories are significantly different, because the remaining genes reflect the host-relationship, harmful or beneficial, respectively.

A future approach that we would like to develop consists of reconstructing the ancestral genomes then describing the evolution of genes using the phylogenetic profile. We would relate the history of gene loss and gene gain of each phylum more precisely in the evolutionary time scale.

**Reviewer**: In the results section of the abstract it is stated that the observation of fewer rRNA genes, split rRNA operons and fewer transcriptional regulators are linked to slower growth rates. I find this usage of the term "linked" unfortunate, as nothing over and above a statistical corellation is presented here. These four measurables are all corellated to one another, the OI lifestyle and any number of other factors characteristic of OI organisms. It may be that there is a rationale to explain a linkage between slow growth rates, rRNA operons and transcriptional regulators, but the authors have neither spelled out that rationale nor suported this linkage as a significant "result" of their work. On a similar note, the results also state that the specialization is an "irreversible phenomenon characterized by translation modification and massive gene loss..." That specialization is irreversible in OI organisms considering their genetic isolation from a gene pool for lateral gene transfer has been suggested in other work and is quite reasonable, but it can hardly be construed as a result of this study, and the particular connection to "translation modification" (changes in the apparatus of translation i.e. rRNAs – author's comment) is unsupported.

**Authors' response**: The PCA in Figure 5 shows that the obligate intracellular reduced genomes have few genes for transcriptional regulation, few rRNA operons and that they do need long time for growth. On the contrary, the fast-growing free-living bacteria have more genes involved in the regulation of transcription and more rRNA operons. We have done a multiple regression analysis to test for correlation between the 3 factors (growth time, transcriptional regulation and rRNA operon numbers). The multiple regression analysis showed that there is a significant relation between the phenotypic character which is the growth time, and the 2 other genomic features.

In what concerns irreversibility, genome size and gene repertoire can increase through gene acquisition, i.e. lateral gene transfer and gene duplication, and conversely, decrease by deletion [3,4]. It is not surprising that the genomes of obligate intracellular bacteria includes the smallest genome of any characterized bacteria, that of *Candidatus Carsonella ruddii*, an endosymbiotic *Gammaproteobacteria*. In fact, the dynamics of bacterial genomes are affected by their niches. On one hand, specialization to an intracellular lifestyle implies a genetic isolation characterized by the diminution of lateral gene transfer. Indeed, a phylogenomic study has shown that bacteria from different phyla cluster together according to their waterborne lifestyle [13]. Audic *et al*., have quantified the lateral gene transfer events and have concluded that bacterial communities living in water have the highest percentage of LGT, whereas intracellular bacteria have the lowest percentage of LGT. On the other hand, Andersson *et al*. [72,75] and Moran *et al*. [8,76] have studied the genome evolution by gene loss, in the parasites in *Alphaproteobacteria *and in the endosymbionts in *Gammaproteobacteria*. Altogether, the reduced genome that results from or follows the restriction of bacteria to a special niche implies massive gene loss that is not compensated by the acquisition of foreign DNA (LGT). Thus, the severe gene loss we described in obligate intracellular genomes comparing to free-living bacteria, may represent an irreversible evolutionary trajectory that constrains existence outside a eukaryotic cell, and limits transitions in lifestyles (e.g. parasitic versus mutualistic associations with hosts) [7,47]. Indeed, the irreversibility is not a result of our paper, but it is an important argument to support the different evolutionary fates of mutualists' and parasites' genomes. Irreversibility is essential in the description of the model we propose: "the one-way road specialization".

Finally it has been suggested that recombination events concerning the duplicated rRNA operons induced genome shuffling and contributed to the rickettsial genome evolution [39,40]. We could show that the altered structure of rRNA operons concerns more than one host-dependent lineage. That's why we judged important to mention this result as it helps in the understanding of one of the mechanisms of evolution.

**Reviewer**: The abstract states that this work identifies the loss of "100 genes" in OI organisms. This is not strictly true in the sense of 100 individual, named orthologous genes, rather, they have identified 100 gene *functions *represented by 100 different COGs (clusters of orthologous groups). Many of these COGs have more than one representative in free-living organisms. Members of these 100 orthologous groups are absent from OI organisms and nearly universal in free-living organisms. Finally, that the statement that mutualists and parasites have lost distinct sets of genes is paired with statements about which types of genes are *retained *is curious and confusing.

**Authors' response**: Our analysis consisted on comparing bacterial genomes from different lifestyles on the basis of their genome content. We used the classification of the genes in the functional categories as defined in the COG database [60,61]. Our method was based on the comparaison of:

(i) the number of genes for each category of COG (copy number of genes) (Table 1)

(ii) and the number of COG present or absent in each bacteria.

Thus, considering the presence or absence of COGs we identified 100 orthologous genes lost in obligate intracellular bacteria and present in free-living bacteria (Table 4). Thank you for this precision, we have highlighted this notion in the abstract.

When comparing the number of genes, free-living bacteria have more genes than obligate intracellular bacteria (Additional file 2), and the difference is more than 100 genes. Thus, obligate intracellular bacteria have lost more than the core of 100 lost COGs. Obviously, we can say that these additional losses are specific to the evolutionary history of each species. Our comparison of the gene contents of mutualists and parasites showed that the gene inventories are specific to each lifestyle. This suggests that the additional losses we talk about are in fact specific to the type of host-relationship.

**Reviewer**: In their abstract conclusion, the authors state that gene loss *rather than *acquisition of virulence factors has been a *driving force *in the adaptation of parasites to eukaryotic cells. I find this a bit hard to take, considering that there is no explicit study of virulence factors in this work, in fact the only mention of virulence factors is in the manuscript's conclusion where a single reference supports a statement about the occurrence of genes *named *virulence factors in free-living organisms compared to pathogenic bacteria, a statement whose character has the distinct odor of transitive annotation error about it.

**Authors' response**: In what concerns the driving force, what we claim makes a lot of sense on evolutionary point of view. Human bacterial pathogens are highly specialized bacteria their evolution obeys to the allotropic model of speciation by Mayr [77]. When specializing to an intracellular lifestyle, bacteria lose their capabilities to survive in another niche because of the loss of genes and genetic isolation. Thus the overall consequence of allotropic speciation is genome reduction and gene loss. The reversibility of this phenomenon has never been demonstrated and is basically against evolutionary principle see [78]. We didn't study the virulence factors in this work but the mention in the manuscript's conclusion can be considered as a prospect. Actually, the evolutionary history of virulence factors would be very interesting to explore in a further work.

**Reviewer**: To support the statement (Results and Discussion, first line) that "Host-dependent bacteria typically have a smaller genome size and fewer genes compared to their close relatives in the same phylum.", Additional file 2 is included. Unfortunately, that plot only establishes that host-dependent organisms trend towards smaller size within phyla, but makes no statements about organisms which are *close relatives in the same phylum*. A phylum is a pretty broad taxonomic grouping. In fact the authors repeatedly use the phrase "close relatives" when trying to relate free-living and host-associated organisms while in fact basing this closeness on broad taxonomic categories and never holding up a specific example. An analysis based on a tree of life (for instance, Wu & Eisen, 2008) would be much more useful. Specific examples would be useful, i.e. what specific free-living organisms can be held up as the *closest relatives *of specific OI organisms and what can be concluded about them?

**Authors' response**: The phylogenetic close relatives are determined according to the current bacterial taxonomy and standard prokaryotic phylogeny, based on sequence similarity of 16S rRNA. In agreement with previous phylogenomic studies [11-13], our work confirms that based on the genome content and the bacterial lifestyle, bacteria may be close relatives to distant phylogenetically related bacteria (on the basis of the 16S rRNA). Thus on the phylogenetic tree in figure 1 we can appreciate the convergent evolution, i.e. similar genomic features (like gene loss and alteration of rRNA operons) occurring in bacteria from distinct phyla but similar habitat.

**Reviewer**: One of the central claims of this work is that genome-reductive adaptation of distantly related organisms to varied intracellular environments is convergent. That is to say, the same sets of gene functions are lost (as represented here by COGs). In order to support this claim it is not sufficient to tally the number of COGs which are lost in all or most OI organisms, considering the total amount of genes lost, some number will be lost in common. The task then is to determine whether the observed number of concerted losses of the same function is statistically significant as compared to a random loss model. The representation of the statistical methods used to prove this point in the main body of the paper and the caption to Additional File 5 are vague, but are detailed in the methods section. Unfortunately, insufficient detail and more important, clarity, is provided in the methods to allow one to fully understand the procedure or to allow replication of their results. Part of the murkiness in the description may result from difficulties in the use of English, for instance the many missing articles, and does "commonly lost" mean "often observed to be lost" or "lost in concert among all test groups"? A more basic question is whether the overall approach, a comparison of only alpha and gamma proteobacterial mutualists and parasites is a valid one. The authors should have spent more effort in the main section of the manuscript outlining why this approach was chosen over other possibilities, and why the resulting statistical significance value proves the point they are trying to prove. Why are they focussing on cases where a COG is "lost by bacteria of the group θ given that it is at least present in mutualists or parasites from α or γ-proteobacteria"? This would be those COGs which are *not *among the "core of lost COGs" because they are present in one of these OI classes. The very next sentence says that this calculation is used to compute the probability for any COG to be "in the set of commonly lost COGs". How is this done?

**Authors' response**: We have modified the method section concerning the test of convergence in order to be more explicit. The "commonly lost" COGs are "COGs lost in concert among all test groups", i.e. core of lost COGs. Alpha and gamma proteobacteria are the unique phyla containing both free-living and obligate intracellular bacteria, in sufficient species number to allow statistical comparative studies to be done. The obligate intracellular bacteria from these two phyla represent a total of 25 bacteria over the 40 studied obligate intracellular bacteria. Add to this, we didn't extrapolate the results of these two phyla to the rest of obligate intracellular bacteria but we searched for the COGs that we found lost in concert among the obligate intracellular bacteria from alpha and gamma Proteobacteria, in the obligate intracellular bacteria of other phyla. Thus the 100 COGs we retained are absent from all obligate intracellular bacteria from different phyla. With the growing number of genome sequences, we can expect to get more sequences for obligate intracellular and free-living bacteria in all phyla, in order to make comparative genomic analyses including phyla remaining unstudied to date. We aimed to prove the non-independent gene loss between phyla, i.e. the obligate intracellular bacteria from different phyla tend to lose the same COGs or they tend to lose no COG in common. For that, we first calculated the probability for an obligate intracellular bacterium to lose a COG among the 969 COGs lost at least by mutualists or parasites from α – or γ-proteobacteria (Additional file 4). We obtained [0.0446, 0.0847] as 95%-confidence interval of p0. Then we calculated the probability P (X ≥ 100) with X having the Binomial distribution B (969, p = 0.0847), this probability was less than 0.05.

Consequently the same holds true for any probability p in the 95%-confidence interval of p_0 _(Additional file 11). This indicates that the obligate intracellular bacteria do not lose COGs independently and thus proves convergent loss phenomenon because the observed common loss probability p_1 _(0.1032) was significantly greater than the probability p_0 _(p_0 _≤ 0.0847 with 95% confidence) of loss in common under the hypothesis of independent gene loss between phyla. Second, we did not restrict our analysis to the set of COGs lost by obligate intracellular bacteria (969 COGs), but we considered also the whole losable COGs, i.e. COGs present in free-living bacteria from α – or γ-proteobacteria (3865 COGs). We obtained [1.6 × 10^-4^, 3.7 × 10^-4^] as 95%-confidence interval of p_0_. The probability P(X ≥ 100) with X having the Binomial distribution B (3865, 3.7 × 10^-4^) was once again less than 0.05. In the final version of the manuscript we only kept the latter test considering the losable COGs (Additional file 6).

**Reviewer**: The authors make much of their correlations between various calculated values and growth rates in particular. For instance, they state that they find a "significant negative correlation between generation time on one side and the number of rRNA operons and transcriptional regulators per Mb on the other side." And then state that all of these are correlated with the OI lifestyle. Of all of the measurables in this work, it is the growth rate data which is most indirect and subject to bias. The authors have compiled growth data from other compilers of such data (in a way that is not easily traceable) and from primary literature from a wide variety of organisms growing under many different conditions with varying relationships to the conditions in which they were evolved to live. They have binned these data into only three discrete categories (fast, medium and slow) and represented those categories by three discrete numbers for their PCA analyses. Considering the highly correlated nature of all of the observables under study here and the over-processed and indirect nature of the growth data, I find little of value is added to this work by its inclusion.

**Authors' response**: We disagree with this statement. This is the first work of this nature published to date, and it may be improved latter. Actually, introducing a wide scale analysis of growth time, transcriptional regulators and ribosome is a real contribution. The ribosome is probably a critical point in the evolution of specialized bacteria. For that, we made an exhaustive literature search looking in previous papers for exact experimentally time of growth for bacteria. And we have asked two international reference collections, Pasteur and CCUG, for help about these data. They agreed that there are a lot of problems to determine precisely the time of growth of bacteria. Thus we looked in literature and in CCUG website  for information concerning the doubling time, or the generation time or the colony observation or plaque formation (We have mentioned all the references we used in additional file 1). The time of growth is given for bacteria in their optimal growth conditions known *nowadays: *plate growth or cell cultures (for fastidious bacteria). We grouped the studied bacteria into 3 categories fast growing (24–48 hours), median (3 to 7 days) and slow growing fastidious bacteria (more than 7 days). We proposed approximate numbers for the 3 categories (2, 5 and 10) that reflect the 3 ordered levels of the experimental growth time. The Principal Component Analysis (PCA) revealed similar behaviors of bacteria on the basis of the gene count of transcriptional regulators, rRNA operon numbers and growth time. The task was difficult to determine precisely the growth time but we consider that it is worthy doing it because it revealed an important correlation between translation, transcription and time of growth. Hope that future experimental works will give more precise determination of generation time that reflects the real growth time of these bacteria.

## Supplementary Material

Additional file 1 **Genome information data.** Click here for file

Additional file 2 **Trends between genome size and gene count in different bacterial phyla**. Columns correspond to the genome size (left axis), and the points correspond to the gene count (right axis). Red and blue colours correspond to host dependent (HD) and free-living (FL) bacteria, respectively. Other phyla: *Aquifex*, *Thermotoga*, *Chlorobium*, *Dehalococcoides*, *Deinococci*, *Thermus*, *Fusobacteria*, *and planctomyces*. Taxa are listed in Additional file 1. Click here for file

Additional file 3 **Schematic representation of strategy used to identify essential and lost COGs**. FL corresponds to free-living and OI corresponds to obligate intracellular bacteria. Click here for file

Additional file 4 **Comparison of the sets of lost COGs**. Venn diagram shows the number of shared and group-specific COGs lost in obligate intracellular bacteria and conserved in their close free-living relatives in alpha- and gamma-proteobacteria. *Mycobacterium leprae *lost only 63% of these COGs, however its genome content is rapidly degrading according to the high number of pseudogene [27]. Click here for file

Additional file 5 **Distribution of the common loss proportion, simulated from 2000 re-samplings**. The number of COGs lost by each phylum is fixed and equals the observed loss numbers. Red dashed lines represent the 2.5% and 97.5% quantiles and blue line indicates the observed common loss proportion. 100 COGs were lost in concert among the obligate intracellular bacteria over the 969 COGs that are lost by at least one of the studied bacteria, which give a proportion of 0.1032 (100/969). The number of common COGs lost was significantly more important than expected if the loss were random (Randomization test, n = 2000, *p *< 10^-6^). Click here for file

Additional file 6 **Probability of losing at least 100 COGs in common **Curve describing the probability of losing at least 100 COGs in common with respect to the theoretical probability of random loss (p0). The red point corresponds to the probability-threshold under which the hypothesis of independent loss between phyla is rejected (with α = 5%). Click here for file

Additional file 7 **Mean number of genes per Mb encoding for intracellular trafficking**. *p-values < 0.05 are shown in bold to indicate significant differences between mutualists and parasites (Wilcoxon rank sum test). Click here for file

Additional file 8 **COGs involved in transcription.** Click here for file

Additional file 9 **COGs involved in intracellular trafficking and secretion.** Click here for file

Additional file 10 **Distribution of COGs lost by small free-living bacteria among the set of 100 COGs lost in common by obligate intracellular bacteria and among all other COGs.** Click here for file

Additional file 11 **Probability of losing at least 100 COGs in common among the 969 COGs lost at least by one obligate intracellular bacterium**. Curve describing the probability of losing at least 100 COGs in common with respect to the theoretical probability of random loss (p0). The red point corresponds to the probability-threshold under which the hypothesis of independent loss between phyla is rejected (with α = 5%). Click here for file
