# Supplementary material for: Massive comparative genomic analysis reveals convergent evolution of specialized bacteria
Source: Biol Direct. 2009 Apr 10;4:13. doi: 10.1186/1745-6150-4-13 (PMC2688493; doi:10.1186/1745-6150-4-13)
Supplement: Additional file 1 — Genome information data. [file 1745-6150-4-13-S1.pdf]

Genome Name	Phylum	Refseq	Lifestyle*	Genome Size(Mb)	GC%	Gene Count	CDS	COG Genes	operon nber	16s_23s_5s	16s_1_23s_5s	16s_2_23s_5s	16s_3_23s_5s	16s_5_23s_5s	16s_4_23s_5s	16s_23s_1_5s	additional§	5s	23s	16s	tRNA	ITS_ length (Mean)	Genes for Regulation	# Growth time	[References]
Bifidobacterium longum NCC2705	Actinobacteria	NC_004307	FHA	2,26	60	1799	1729	1336	**4**	4											57	423	89	24 - 48h	[1]
Corynebacterium diphtheriae NCTC 13129	Actinobacteria	NC_002935	FL	2,49	53	2349	2272	1576	**5**	5											54	460	73	24 - 48h	[2]
Corynebacterium efficiens YS-314	Actinobacteria	NC_004369	FL	3,15	63	3020	2950	2042	**5**	5											55	463	7	24 - 48h	[2]
Corynebacterium glutamicum ATCC 13032 (Bielefeld)	Actinobacteria	NC_006958	FL	3,28	54	3147	3057	2129	**6**	6											60	390	108	24 - 48h	[2]
Corynebacterium glutamicum ATCC 13032 (Kitasato)	Actinobacteria	NC_003450	FL	3,31	54	3073	2993	2123	**5**	5											60	391	107	24 - 48h	[2]
Corynebacterium jeikeium K411	Actinobacteria	NC_007164	FL	2,48	61	2181	2120	1559	**3**	3											50	413	53	24 - 48h	[2]
Mycobacterium avium paratuberculosis K-10	Actinobacteria	NC_002944	FHA	4,83	69	4412	4350	3188	**1**	1											46	1030	141	> 7 days	[3]
Mycobacterium bovis AF2122/97	Actinobacteria	NC_002945	FHA	4,35	66	3983	3920	2760	**1**	1											45	276	114	> 7 days	
Mycobacterium leprae TN	Actinobacteria	NC_002677	P	3,27	58	1663	1605	1175	**1**	1											45	1048	40	> 7 days	
Mycobacterium tuberculosis CDC1551	Actinobacteria	NC_002755	FHA	4,40	66	4237	4189	2716	**1**	1											45	277	116	> 7 days	
Mycobacterium tuberculosis H37Rv	Actinobacteria	NC_000962	FHA	4,41	66	4052	3989	2807	**1**	1											45	276	114	> 7 days	
Nocardia farcinica IFM 10152	Actinobacteria	NC_006361	FL	6,29	71	6000	5936	3814	**3**	3											53	307	237	3 days	[4]
Propionibacterium acnes KPA171202	Actinobacteria	NC_006085	FL	2,56	60	2351	2297	1689	**3**	3											45	385	80	48h	[2]
Rhodococcus sp. RHA1	Actinobacteria	NC_008268	FL	9,70	67	9210	9145	6069	**4**	4											50	351	399	48h	[2]
Streptomyces avermitilis MA-4680	Actinobacteria	NC_003155	FL	9,12	71	7761	7673	5044	**6**	6									1			68	307	408	48 - 72h	[5]
Streptomyces coelicolor A3(2)	Actinobacteria	NC_003888	FL	9,05	72	8263	8154	5261	**6**	6												66	197	455	2 - 5 days	[6]
Symbiobacterium thermophilum IAM 14863	Acidobacteria	NC_006177	FL	3,57	69	3476	3337	2464	**6**	6												99	217	98	24h	[7]
Thermobifida fusca YX	Actinobacteria	NC_007333	FL	3,64	68	3184	3117	2239	**4**	4												52	523	130		
Tropheryma whipplei TW08/27	Actinobacteria	NC_004551	FHA	0,93	46	840	783	620	**1**	1												52	294	13	> 7 days	[8]
Tropheryma whipplei Twist	Actinobacteria	NC_004572	FHA	0,93	46	864	808	624	**1**	1												53	296	13	> 7 days	[8]
Aquifex aeolicus VF5	Aquificae	NC_000918	FL	1,59	43	1613	1560	1346	**2**			2									44	315	24	3 - 5 days	[9]
Bacteroides fragilis NCTC 9343	Bacteroidetes	NC_003228	FHA	5,24	43	4323	4231	2543	**6**			6						1			73	478	43	24 - 48h	[2]
Bacteroides fragilis YCH46	Bacteroidetes	NC_006347	FHA	5,31	43	4717	4625	2600	**6**			6									74	481	44	24 - 48h	[2]
Bacteroides thetaiotaomicron VPI-5482	Bacteroidetes	NC_004663	FHA	6,29	43	4917	4816	2900	**5**			5									71	590	56	24 - 48h	[2]
Porphyromonas gingivalis W83	Bacteroidetes	NC_002950	FL	2,34	48	1984	1909	1223	**4**			4									53	835	20	4 - 7 days	[2]
Salinibacter ruber DSM 13855	Bacteroidetes	NC_007677	FL	3,59	66	2881	2833	2030	**1**			1									44	569	59	DT 14 - 18h	[10]
Candidatus Protochlamydia amoebophila UWE25	Chlamydiae	NC_005861	P	2,41	35	2075	2031	1127	**3**	3											35	1	20		
Chlamydia muridarum Nigg	Chlamydiae	NC_002620	P	1,08	40	957	911	629	**2**	2											37	242	7	cell cycle 18h<br>cell lysis 40-48 h	[11]
Chlamydia trachomatis A/HAR-13	Chlamydiae	NC_007429	P	1,05	41	961	919	640	**2**	2											37	316	8	72h	[12]
Chlamydia trachomatis D/UW-3/CX	Chlamydiae	NC_000117	P	1,04	41	941	895	634	**2**	2											37	316	8	72 h	[12]
Chlamydophila abortus S26/3	Chlamydiae	NC_004552	P	1,14	40	974	932	649	**1**	1											38	310	7	72h	[13]

Genome Name	Phylum	Refseq	Lifestyle*	Genome Size(Mb)	GC%	Gene Count	CDS	COG Genes	operon nber	16s_23s_5s	16s_1_23s_5s	16s_2_23s_5s	16s_3_23s_5s	16s_5_23s_5s	16s_4_23s_5s	16s_23s_1_5s	additional§	5s	23s	16s	tRNA	ITS_ length (Mean)	Genes for Regulation	# Growth time	[References]
Chlamydophila caviae GPIC	Chlamydiae	NC_003361	P	1,18	39	1049	1005	658	**1**	1											38	311	9	3 - 5 days	
Chlamydophila felis Fe/C-56	Chlamydiae	NC_007899	P	1,17	39	1054	1013	676	**1**	1											38	225	95	72h	[14]
Chlamydophila pneumoniae AR39	Chlamydiae	NC_002179	P	1,23	41	1156	1112	665	**1**	1											38	308	8	3 - 7 days	[15]
Chlamydophila pneumoniae CWL029	Chlamydiae	NC_000922	P	1,23	41	1096	1052	669	**1**	1											38	300	8	3 - 7 days	[15]
Chlamydophila pneumoniae J138	Chlamydiae	NC_002491	P	1,23	41	1107	1069	671	**1**	1											38	308	8	3 - 7 days	[15]
Chlamydophila pneumoniae TW-183	Chlamydiae	NC_005043	P	1,23	41	1157	1113	670	**1**	1											38	300	8	3 - 7 days	[15]
Chlorobium chlorochromatii CaD3	Chlorobi	NC_007514	FL	2,57	44	2047	2002	1452	**1**	1											45	457	28		
Chlorobium tepidum TLS	Chlorobi	NC_002932	FL	2,15	57	2308	2252	1502	**2**			2									50	526	25	24 - 48h	[16]
Pelodictyon luteolum DSM 273	Chlorobi	NC_007512	FL	2,36	57	2137	2083	1611	**2**			2									48	577	38		
Dehalococcoides ethenogenes 195	Chloroflexi	NC_002936	FL	1,47	49	1631	1580	1119	**0**									1	1	1	46		54		
Dehalococcoides sp. CBDB1	Chloroflexi	NC_007356	FL	1,40	47	1510	1458	1091	**0**									1	1	1	47		59	> 7 days	[17]
Anabaena variabilis ATCC 29413	Cyanobacteria	NC_007413	FL	7,07	41	5763	5701	3688	**4**	1		3									47	502	99	24 - 48h	[18]
Gloeobacter violaceus PCC 7421	Cyanobacteria	NC_005125	FL	4,66	62	4482	4430	2869	**1**	1											45	459	106	3 - 5 days	[19]
Nostoc sp. PCC 7120	Cyanobacteria	NC_003272	FL	7,21	41	6213	6130	3802	**4**	1		3									48	287	104	48h	[20]
Prochlorococcus marinus marinus CCMP1375	Cyanobacteria	NC_005042	FL	1,75	36	1932	1883	1167	**2**			2									44	830	16	DT 6h + circadian rythm	[21,22]
Prochlorococcus marinus MIT 9312	Cyanobacteria	NC_007577	FL	1,71	31	1856	1811	1182	**1**			1									39	541	16	DT 6h + circadian rythm	
Prochlorococcus marinus MIT 9313	Cyanobacteria	NC_005071	FL	2,41	51	2333	2275	1440	**1**			1									38	690	23	DT 6h + circadian rythm	
Prochlorococcus marinus NATL2A	Cyanobacteria	NC_007335	FL	1,84	35	1940	1896	1175	**1**			1									40	667	15	DT 6h + circadian rythm	
Prochlorococcus marinus pastoris CCMP1986	Cyanobacteria	NC_005072	FL	1,66	31	1765	1719	1174	**1**			1									37	549	14	DT 6h + circadian rythm	
Synechococcus elongatus PCC 6301	Cyanobacteria	NC_006576	FL	2,70	55	2582	2527	1833	**2**			2									45	546	36	24h	[23]
Synechococcus elongatus PCC 7942	Cyanobacteria	NC_007604	FL	2,74	55	2715	2662	1869	**2**	2											44	546	40	24h	[24]
Synechococcus sp. CC9311	Cyanobacteria	NC_008319	FL	2,61	52	2944	2892	1612	**2**			2									44	763	24	24h	[25]
Synechococcus sp. CC9605	Cyanobacteria	NC_007516	FL	2,51	59	2756	2702	1556	**2**			2									45	796	22	24h	
Synechococcus sp. CC9902	Cyanobacteria	NC_007513	FL	2,23	54	2358	2307	1459	**2**	2											45	778	18	24h	
Synechococcus sp. JA-2-3Ba(2-13)	Cyanobacteria	NC_007776	FL	3,05	58	2914	2862	1968	**2**			2									45	729	41	24h	
Synechococcus sp. JA-3-3Ab	Cyanobacteria	NC_007775	FL	2,93	60	2815	2760	1874	**2**			2									47	556	42	24h	
Synechococcus sp. WH 8102	Cyanobacteria	NC_005070	FL	2,43	59	2586	2528	1560	**2**			2									44	808	26	DT 15 - 23h	[26]
Synechocystis sp. PCC 6803	Cyanobacteria	NC_000911	FL	3,95	47	3619	3569	2473	**2**		2										43	466	50	1 - 3 days	[27]
Thermosynechococcus elongatus BP-1	Cyanobacteria	NC_004113	FL	2,59	54	2525	2476	1765	**1**			1									42	421	31	DT 20 h	[28]
Deinococcus radiodurans R1	Deinococcus-	NC_001263	FL	3,28	67	3248	3181	2292	**3**	1		2									49		91	24 - 48h	[2]
Thermus thermophilus HB27	Thermus	NC_005835	FL	2,13	69	2272	2210	1742	**0**									2	2	2	47		46	24 - 48 h	[29]

Genome Name	Phylum	Refseq	Lifestyle*	Genome Size(Mb)	GC%	Gene Count	CDS	COG Genes	operon nber	16s_23s_5s	16s_1_23s_5s	16s_2_23s_5s	16s_3_23s_5s	16s_5_23s_5s	16s_4_23s_5s	16s_23s_1_5s	additional§	5s	23s	16s	tRNA	ITS_ length (Mean)	Genes for Regulation	# Growth time	[References]
Thermus thermophilus HB8	Thermus	NC_006461	FL	2,12	69	2291	2238	1748	**0**									2	2	2	47		49	24 - 48 h	[30]
Aster yellows witches-broom phytoplasma AYWB	Firmicutes	NC_007716	M	0,72	27	728	693	393	**2**		2										31	246	1	> 7 days	[31]
Bacillus anthracis Ames	Firmicutes	NC_003997	FL	5,23	35	5536	5311	3430	**11**	9		2									95	171	246	24 - 48h	[2]
Bacillus anthracis Ames Ancestor	Firmicutes	NC_007530	FL	5,50	35	5745	5617	3488	**11**	9		2									95	179	249	24 - 48h	[2]
Bacillus anthracis Sterne	Firmicutes	NC_005945	FL	5,23	35	5415	5287	3639	**11**	9		2									95	169	250	24 - 48h	[2]
Bacillus cereus ATCC 10987	Firmicutes	NC_003909	FL	5,43	35	6080	5844	3675	**12**	10		2									98	137	270	24 - 48h	[2]
Bacillus cereus ATCC 14579	Firmicutes	NC_004722	FL	5,43	35	5502	5255	3510	**13**	11		2									108	182	244	24 - 48h	[2]
Bacillus cereus E33L	Firmicutes	NC_006274	FL	5,84	35	5776	5641	3815	**13**	11		2									96	175	285	24 - 48h	[2]
Bacillus clausii KSM-K16	Firmicutes	NC_006582	FL	4,30	45	4192	4096	3172	**7**	6		1						1			74		228	24 - 48h	[2]
Bacillus halodurans C-125	Firmicutes	NC_002570	FL	4,20	44	4171	4066	3088	**8**	6		2						1			78	683	190	24 - 48h	[2]
Bacillus licheniformis ATCC 14580 (Goettingen)	Firmicutes	NC_006270	FL	4,22	46	4289	4196	3130	**7**	5		2									72	170	206	24 - 48h	[2]
Bacillus licheniformis ATCC 14580 (Novozymes)	Firmicutes	NC_006322	FL	4,22	46	4245	4152	3091	**7**	5		2									72	170	209	24 - 48h	[2]
Bacillus subtilis 168	Firmicutes	NC_000964	FL	4,21	44	4291	4105	3048	**10**	8		2									86	166	199	24 - 48h	[2]
Bacillus thuringiensis sv konkukian 97-27	Firmicutes	NC_005957	FL	5,31	35	5341	5197	3608	**13**	11		2						2	2	2	105	175	244	24 - 48h	[2]
Carboxydothermus hydrogenoformans Z-2901	Firmicutes	NC_007503	FL	2,40	42	2683	2620	1898	**4**	3		1									50	341	64	24 - 48h	[32]
Clostridium acetobutylicum ATCC 824	Firmicutes	NC_003030	FL	4,13	31	4017	3848	2808	**11**	11											73	178	196	24 - 48h	[2]
Clostridium perfringens ATCC 13124	Firmicutes	NC_008261	FL	3,26	28	2993	2876	2068	**8**	6		2									93	186	99	24 - 48h	[2]
Clostridium perfringens SM101	Firmicutes	NC_008262	FL	2,96	28	2726	2631	1922	**10**	8		2						1			94	415	94	24 - 48h	[2]
Clostridium perfringens str. 13	Firmicutes	NC_003366	FL	3,09	29	2848	2723	2022	**10**	9		1									96	189	96	24 - 48h	[2]
Clostridium tetani E88	Firmicutes	NC_004557	FL	2,87	29	2558	2432	1853	**6**	3	2	1									54	265	84	24 - 48h	[2]
Desulfitobacterium hafniense Y51	Firmicutes	NC_007907	FL	5,73	47	5137	5060	3856	**6**	2	3	1									59	536	242	24 - 48h	[33]
Desulfotomaculum reducens MI-1	Firmicutes	NC_007519	FL	3,61	42	3424	3324	2409	**4**			4									71	461	69		
Enterococcus faecalis V583	Firmicutes	NC_004668	FL	3,36	37	3384	3265	2210	**4**	2	2										68	259	113	24 - 48h	[2]
Geobacillus kaustophilus HTA426	Firmicutes	NC_006510	FL	3,59	52	3654	3540	2594	**9**	7		2									87	341	117	24 - 48h	[34]
Lactobacillus acidophilus NCFM	Firmicutes	NC_006814	FHA	1,99	35	1938	1864	1433	**3**	2		1						1	2	1	61	133	61	24 - 48h	[2]
Lactobacillus brevis ATCC 367	Firmicutes	NC_008497	FL	2,34	46	2300	2218	1678	**5**	3		2						1			65	419	115	2 - 4 days	[35]
Lactobacillus casei ATCC 334	Firmicutes	NC_008526	FL	2,92	47	2847	2771	1959	**5**	3		2						1			59	829	111	24 - 48h	[2]
Lactobacillus delbrueckii bulgaricus ATCC 11842	Firmicutes	NC_008054	FL	1,86	50	1684	1562	1153	**9**	5		4									95	466	40	24 - 48h	[2]
Lactobacillus delbrueckii bulgaricus ATCC BAA-365	Firmicutes	NC_008529	FL	1,86	50	1848	1721	1196	**9**	4		5						1			98	566	48	24 - 48h	[2]
Lactobacillus gasseri ATCC 33323	Firmicutes	NC_008530	FL	1,89	35	1853	1755	1316	**6**	4		2						1			78	553	57	24 - 48h	[2]
Lactobacillus johnsonii NCC 533	Firmicutes	NC_005362	FHA	1,99	35	1936	1821	1403	**6**	4		2									79	100	57	24 - 48h	[2]

Genome Name	Phylum	Refseq	Lifestyle*	Genome Size(Mb)	GC%	Gene Count	CDS	COG Genes	operon nber	16s_23s_5s	16s_1_23s_5s	16s_2_23s_5s	16s_3_23s_5s	16s_5_23s_5s	16s_4_23s_5s	16s_23s_1_5s	additional§	5s	23s	16s	tRNA	ITS_ length (Mean)	Genes for Regulation	# Growth time	[References]
Lactobacillus plantarum WCFS1	Firmicutes	NC_004567	FHA	3,35	44	3182	3059	2305	**5**	3		2						1			70	206	146	24 - 48h	[2]
Lactobacillus sakei sakei 23K	Firmicutes	NC_007576	FHA	1,88	41	1963	1879	1462	**7**	6		1									63	434	82	24 - 48h	[2]
Lactobacillus salivarius salivarius UCC118	Firmicutes	NC_007929	FHA	2,13	33	2116	2017	1476	**7**	5		2									78	409	67	24 - 48h	[2]
Lactococcus lactis lactis Il1403	Firmicutes	NC_002662	FHA	2,37	35	2424	2321	1756	**6**		6							1			61	305	81	3 days	[36]
Leuconostoc mesenteroides mesenteroides ATCC 8293	Firmicutes	NC_008531	FL	2,08	38	2090	2005	1547	**4**		4										71	376	69	24 - 48h	[2]
Listeria innocua Clip11262	Firmicutes	NC_003212	FL	3,09	37	3172	3043	2391	**6**	4		2									66	246	144	24 - 48h	[2]
Listeria monocytogenes 4b F2365	Firmicutes	NC_002973	FL	2,91	38	2964	2821	2266	**6**	4		2									67	245	140	24 - 48h	[2]
Listeria monocytogenes EGD-e	Firmicutes	NC_003210	FL	2,94	38	2976	2846	2335	**6**	4		2						1			68	245	149	24 - 48h	[2]
Mesoplasma florum L1	Firmicutes	NC_006055	FHA	0,79	27	717	682	510	**2**	2											29	208	12		
Moorella thermoacetica ATCC 39073	Firmicutes	NC_007644	FL	2,63	56	2615	2523	2008	**1**			1						1			51	414	92		
Mycoplasma capricolum capricolum ATCC 27343	Firmicutes	NC_007633	FHA	1,01	24	854	812	535	**2**	2								1			30	232	8	5 - 7 days	
Mycoplasma gallisepticum R	Firmicutes	NC_004829	FHA	1,00	31	769	726	468	**0**									2	2	2	33	661	3	3 - 5 days	
Mycoplasma genitalium G37	Firmicutes	NC_000908	FHA	0,58	32	520	477	387	**1**	1											36	203	3	GT 24h	[37]
Mycoplasma hyopneumoniae 232	Firmicutes	NC_006360	FHA	0,89	29	727	691	431	**0**									1	1	1	30	467	5	3 - 5 days	
Mycoplasma hyopneumoniae 7448	Firmicutes	NC_007332	FHA	0,92	28	696	663	437	**0**									1	1	1	30	484	5	3 - 5 days	
Mycoplasma hyopneumoniae J	Firmicutes	NC_007295	FHA	0,90	29	698	665	433	**0**									1	1	1	30	492	5	3 - 5 days	
Mycoplasma mobile 163K	Firmicutes	NC_006908	FHA	0,78	25	667	633	442	**0**									1	1	1	28	303	7	5 days	[38]
Mycoplasma mycoides mycoides SC PG1	Firmicutes	NC_005364	FHA	1,21	24	1061	1016	622	**2**	2											30	224	14	3 - 5 days	
Mycoplasma penetrans HF-2	Firmicutes	NC_004432	FHA	1,36	26	1069	1037	601	**1**	1											29	299	11	3 - 7 days	[39]
Mycoplasma pneumoniae M129	Firmicutes	NC_000912	FHA	0,82	40	733	689	426	**1**	1											37	233	3	3 - 5 days	
Mycoplasma pulmonis UAB CTIP	Firmicutes	NC_002771	FHA	0,96	27	820	782	517	**0**									2	1	1	29	285	6		
Mycoplasma synoviae 53	Firmicutes	NC_007294	FHA	0,80	28	713	672	448	**0**									3	2	2	34	260	6	3 - 5 days	
Oceanobacillus iheyensis HTE831	Firmicutes	NC_004193	FL	3,63	36	3592	3500	2669	**7**	6		1						1			76	234	141	24 - 48 h	[40]
Oenococcus oeni PSU-1	Firmicutes	NC_008528	FL	1,78	38	1742	1691	1318	**2**		2										43	386	58	5 days	[41]
Onion yellows phytoplasma OY-M	Firmicutes	NC_005303	M	0,86	28	793	754	477	**2**							2					32	25	1		
Pediococcus pentosaceus ATCC 25745	Firmicutes	NC_008525	FL	1,83	37	1827	1755	1389	**5**	3		2									55	418	75		
Staphylococcus aureus RF122	Firmicutes	NC_007622	FL	2,74	33	2591	2515	1903	**5**	2	2	1						1			55	442	82	24 - 48h	[2]
Staphylococcus aureus subsp. aureus COL	Firmicutes	NC_002951	FL	2,81	33	2691	2618	1954	**6**	4	1	1						1			53	303	84	24 - 48h	
Staphylococcus aureus subsp. aureus MRSA252	Firmicutes	NC_002952	FL	2,90	33	2757	2656	2000	**5**	3	1	1						1			60	365	90	24 - 48h	
Staphylococcus aureus subsp. aureus MSSA476	Firmicutes	NC_002953	FL	2,82	33	2702	2598	1957	**6**	4	1	1						1			60	303	82	24 - 48h	
Staphylococcus aureus subsp. aureus Mu50	Firmicutes	NC_002758	FL	2,90	33	2808	2731	2048	**5**	3	1	1						1			60	336	87	24 - 48h	

Genome Name	Phylum	Refseq	Lifestyle*	Genome Size(Mb)	GC%	Gene Count	CDS	COG Genes	operon nber	16s_23s_5s	16s_1_23s_5s	16s_2_23s_5s	16s_3_23s_5s	16s_5_23s_5s	16s_4_23s_5s	16s_23s_1_5s	additional§	5s	23s	16s	tRNA	ITS_ length (Mean)	Genes for Regulation	# Growth time	[References]
Staphylococcus aureus subsp. aureus MW2	Firmicutes	NC_003923	FL	2,82	33	2712	2632	1973	**6**	4	1	1						1			60	304	82	24 - 48h	
Staphylococcus aureus subsp. aureus N315	Firmicutes	NC_002745	FL	2,84	33	2698	2619	2036	**5**	2	1	2						1			62	337	89	24 - 48h	
Staphylococcus aureus subsp. aureus NCTC 8325	Firmicutes	NC_007795	FL	2,82	33	2969	2892	1987	**5**	3	1	1						1			61	365	84	24 - 48h	
Staphylococcus aureus subsp. aureus USA300	Firmicutes	NC_007793	FL	2,92	33	2674	2604	2012	**5**	3	1	1						1			53	365	86	24 - 48h	
Staphylococcus epidermidis ATCC 12228	Firmicutes	NC_004461	FL	2,56	32	2600	2485	1847	**5**	3	1	1						1			60	262	73	24 - 48h	
Staphylococcus epidermidis RP62A	Firmicutes	NC_002976	FL	2,64	32	2609	2526	1870	**6**	4	1	1						1			61	262	77	24 - 48h	
Staphylococcus haemolyticus JCSC1435	Firmicutes	NC_007168	FL	2,69	33	2752	2676	2014	**5**	4	1							1			59	338	101	24 - 48h	
Staphylococcus saprophyticus saprophyticus ATCC 15305	Firmicutes	NC_007350	FL	2,58	33	2596	2514	2042	**6**	4	1	1									61	284	102	24 - 48h	
Streptococcus agalactiae 2603V/R	Firmicutes	NC_004116	FL	2,16	36	2242	2124	1592	**7**		7										80	317	81	24 - 48 h	[2]
Streptococcus agalactiae A909	Firmicutes	NC_007432	FL	2,13	36	2098	1996	1590	**7**		7										80	319	76	24 - 48h	
Streptococcus agalactiae NEM316	Firmicutes	NC_004368	FL	2,21	36	2214	2094	1582	**7**		7										80	318	78	24 - 48h	
Streptococcus mutans UA159	Firmicutes	NC_004350	FL	2,03	37	2055	1960	1522	**5**		5										65	389	87	24 - 48h	
Streptococcus pneumoniae D39	Firmicutes	NC_008533	FL	2,05	40	1987	1914	1499	**4**		4										58	324	70	24 - 48h	
Streptococcus pneumoniae R6	Firmicutes	NC_003098	FL	2,04	40	2139	2043	1584	**4**		4										58	271	72	24 - 48h	
Streptococcus pneumoniae TIGR4	Firmicutes	NC_003028	FL	2,16	40	2189	2094	1542	**4**		4										58	336	76	24 - 48h	
Streptococcus pyogenes M1 GAS	Firmicutes	NC_002737	FL	1,85	39	1790	1697	1341	**7**		7										60	459	64	24 - 48h	
Streptococcus pyogenes MGAS10270	Firmicutes	NC_008022	FL	1,93	38	2068	1987	1415	**6**	4	2										63	426	64	24 - 48h	
Streptococcus pyogenes MGAS10394	Firmicutes	NC_006086	FL	1,90	39	1971	1886	1387	**6**		6										67	421	62	24 - 48h	
Streptococcus pyogenes MGAS10750	Firmicutes	NC_008024	FL	1,94	38	2060	1979	1432	**6**	4	2										63	426	64	24 - 48h	
Streptococcus pyogenes MGAS2096	Firmicutes	NC_008023	FL	1,86	39	1979	1898	1413	**6**	4	2										63	426	65	24 - 48h	
Streptococcus pyogenes MGAS315	Firmicutes	NC_004070	FL	1,90	39	1967	1865	1369	**6**		6										67	416	62	24 - 48h	
Streptococcus pyogenes MGAS5005	Firmicutes	NC_007297	FL	1,84	39	1950	1865	1393	**6**		6										67	421	63	24 - 48h	
Streptococcus pyogenes MGAS6180	Firmicutes	NC_007296	FL	1,90	38	1977	1894	1378	**6**		6										65	426	64	24 - 48h	
Streptococcus pyogenes MGAS8232	Firmicutes	NC_003485	FL	1,90	39	1947	1845	1381	**6**	5	1										66	459	64	24 - 48h	
Streptococcus pyogenes MGAS9429	Firmicutes	NC_008021	FL	1,84	39	1962	1877	1356	**6**		6										67	426	62	24 - 48h	
Streptococcus pyogenes SSI-1	Firmicutes	NC_004606	FL	1,89	39	1928	1861	1348	**5**		5										57	422	62	24 - 48h	
Streptococcus thermophilus CNRZ1066	Firmicutes	NC_006449	FL	1,80	39	2000	1915	1465	**6**		6										67	266	47	24 - 48h	
Streptococcus thermophilus LMD-9	Firmicutes	NC_008500	FL	1,86	39	1803	1716	1262	**6**		6								1			67	278	43	24 - 48h	
Streptococcus thermophilus LMG 18311	Firmicutes	NC_006448	FL	1,80	39	1974	1889	1453	**6**		6										61	266	45	24 - 48h	
Thermoanaerobacter tengcongensis MB4	Firmicutes	NC_003869	FL	2,69	38	2692	2588	1947	**4**	3		1									55	148	78	48 h	[42]
Ureaplasma parvum sv 3 ATCC 700970	Firmicutes	NC_002162	FHA	0,75	25	655	614	409	**2**	2											30	348	4	24 - 48h	[2]

Genome Name	Phylum	Refseq	Lifestyle*	Genome Size(Mb)	GC%	Gene Count	CDS	COG Genes	operon nber	16s_23s_5s	16s_1_23s_5s	16s_2_23s_5s	16s_3_23s_5s	16s_5_23s_5s	16s_4_23s_5s	16s_23s_1_5s	additional§	5s	23s	16s	tRNA	ITS_ length (Mean)	Genes for Regulation	# Growth time	[References]
Fusobacterium nucleatum nucleatum ATCC 25586	Fusobacteria	NC_003454	FL	2,17	27	2129	2067	1531	**5**	5											47	159	39	48 - 72h	[2]
Rhodopirellula baltica SH 1	Planctomycetes	NC_005027	FL	7,15	55	7413	7325	3135	**0**									1	1	1	79		66	5 days	[43]
Anaplasma marginale St. Maries	Proteobacteria (alpha)	NC_004842	P	1,20	50	986	949	688	**0**									1	1	1	37		5	1 - 3 weeks	
Anaplasma phagocytophilum HZ	Proteobacteria (alpha)	NC_007797	P	1,47	42	1306	1264	679	**0**									1	1	1	37		4	1 - 3 weeks	[44,45]
Bartonella henselae Houston-1	Proteobacteria (alpha)	NC_005956	FHA	1,93	38	1541	1488	1137	**2**			2						1			44	1256	17	5 - 7 days	[46]
Bartonella quintana Toulouse	Proteobacteria (alpha)	NC_005955	FHA	1,58	39	1191	1142	986	**2**			2									42	1206	12	5 - 7 days	
Brucella abortus bv 1 9-941	Proteobacteria (alpha)	NC_006932 NC_006933	FHA	3,29	57	3149	3085	2389	**3**			3									55	790	112	3 - 5 days	[2]
Brucella melitensis 16M	Proteobacteria (alpha)	NC_003317 NC_003318	FHA	3,29	57	3282	3198	2615	**3**	1	1	2						1			55	791	112	3 - 5 days	[2]
Brucella melitensis bv Abortus 2308	Proteobacteria (alpha)	NC_007618 NC_007624	FHA	3,28	57	3102	3034	2385	**3**			3									60	790	109	3 - 5 days	[2]
Brucella suis 1330	Proteobacteria (alpha)	NC_004310 NC_004311	FHA	3,32	57	3335	3271	2475	**4**	1		3									55	763	109	3 - 5 days	[2]
Candidatus Pelagibacter ubique HTCC1062	Proteobacteria (alpha)	NC_007205	FL	1,31	30	1389	1354	1131	**1**			1									32	270	21		
Caulobacter crescentus CB15	Proteobacteria (alpha)	NC_002696	FL	4,02	67	3810	3737	2886	**2**			2									51	690	138	24 - 48h	[47]
Ehrlichia canis Jake	Proteobacteria (alpha)	NC_007354	P	1,32	29	984	942	665	**0**									1	1	1	36		5	> 7 days	[48]
Ehrlichia chaffeensis Arkansas	Proteobacteria (alpha)	NC_007799	P	1,18	30	1148	1105	668	**0**									1	1	1	37		5	> 7 days	
Ehrlichia ruminantium Gardel	Proteobacteria (alpha)	NC_006831	P	1,50	28	989	950	671	**0**									1	1	1	36		6	> 7 days	
Ehrlichia ruminantium Welgevonden (ARC-OVI)	Proteobacteria (alpha)	NC_006832	P	1,52	27	929	888	660	**0**									1	1	1	36		6	> 7 days	
Ehrlichia ruminantium Welgevonden (CIRAD)	Proteobacteria (alpha)	NC_005295	P	1,51	27	997	958	678	**0**									1	1	1	36		6	> 7 days	
Erythrobacter litoralis HTCC2594	Proteobacteria (alpha)	NC_007722	FL	3,05	63	3056	3011	2145	**1**			1									44	814	56	4 days	[49]
Gluconobacter oxydans 621H	Proteobacteria (alpha)	NC_006677	FL	2,92	61	2731	2664	1952	**4**			4									50	663	64	24 - 48h	[2]
Jannaschia sp. CCS1	Proteobacteria (alpha)	NC_007802	FL	4,40	62	4336	4283	3175	**1**			1									42	780	135		
Magnetospirillum magneticum AMB-1	Proteobacteria (alpha)	NC_007626	FL	4,97	65	4563	4559	3098	**2**		2										46	403	111	3 - 5 days	[50]
Neorickettsia sennetsu Miyayama	Proteobacteria (alpha)	NC_007798	P	0,86	41	970	932	586	**0**									1	1	1	33		4	> 7 days	[48]
Nitrobacter winogradskyi Nb-255	Proteobacteria (alpha)	NC_007406	FL	3,40	62	3198	3143	2294	**1**			1									49	823	55		
Novosphingobium aromaticivorans DSM 12444	Proteobacteria (alpha)	NC_007794	FL	3,56	65	3412	3338	2498	**3**			3									57	672	98		
Rhodobacter sphaeroides 2.4.1	Proteobacteria (alpha)	NC_007493	FL	4,60	69	4369	4304	3267	**3**			3						1			42	666	115	3 days	[2]
Rhodopseudomonas palustris BisB18	Proteobacteria (alpha)	NC_007925	FL	5,51	65	5016	4943	3688	**2**			2									50	1090	122	3 - 5 days	[51]
Rhodopseudomonas palustris CGA009	Proteobacteria (alpha)	NC_005296	FL	5,47	65	4918	4838	3791	**2**			2						1			49	748	118	3 - 5 days	
Rhodopseudomonas palustris HaA2	Proteobacteria (alpha)	NC_007778	FL	5,33	66	4772	4712	3637	**1**			1									50	816	114	3 - 5 days	

Genome Name	Phylum	Refseq	Lifestyle*	Genome Size(Mb)	GC%	Gene Count	CDS	COG Genes	operon nber	16s_23s_5s	16s_1_23s_5s	16s_2_23s_5s	16s_3_23s_5s	16s_5_23s_5s	16s_4_23s_5s	16s_1_23s_1_5s	additional§	5s	23s	16s	tRNA	ITS_length (Mean)	Genes for Regulation	# Growth time	[References]
Rhodospirillum rubrum ATCC 11170	Proteobacteria (alpha)	NC_007643	FL	4,41	65	3920	3850	3048	**4**			4						1			55	766	115	5 days	
Rickettsia bellii RML369-C	Proteobacteria (alpha)	NC_007940	P	1,52	32	1469	1429	954	**0**									1	1	1	34		10	> 7 days	[52]
Rickettsia conorii Malish 7	Proteobacteria (alpha)	NC_003103	P	1,27	32	1414	1374	855	**0**									1	1	1	33		8	> 7 days	[53]
Rickettsia felis URRWXCal2	Proteobacteria (alpha)	NC_007109	P	1,59	33	1551	1512	1037	**0**									1	1	1	33		10	> 7 days	
Rickettsia prowazekii Madrid E	Proteobacteria (alpha)	NC_000963	P	1,11	29	875	835	713	**0**									1	1	1	33		5	> 7 days	
Rickettsia typhi Wilmington	Proteobacteria (alpha)	NC_006142	P	1,11	29	877	838	704	**0**									1	1	1	33		4	> 7 days	
Roseobacter denitrificans OCh 114	Proteobacteria (alpha)	NC_008209	FL	4,33	59	4171	4129	3076	**1**			1									38	885	113	3 - 4 days	[54]
Silicibacter pomeroyi DSS-3	Proteobacteria (alpha)	NC_003911	FL	4,60	64	4316	4252	3399	**3**			3									53	891	173	3 - 5 days	[55]
Wolbachia endosymbiont of *Drosophila melanogaster*	Proteobacteria (alpha)	NC_002978	M	1,27	35	1239	1195	753	**0**									1	1	1	34		5	> 7 days	[56]
Wolbachia endosymbiont TRS of *Brugia malayi*	Proteobacteria (alpha)	NC_006833	M	1,08	34	842	805	598	**0**									1	1	1	34		2	> 7 days	
Zymomonas mobilis mobilis ZM4	Proteobacteria (alpha)	NC_006526	FL	2,06	46	2058	1998	1432	**3**			3									51	607	41	3 - 5 days	[2]
Azoarcus sp. BH72	Proteobacteria (beta)	NC_006513	FL	4,38	68	4054	3989	3226	**4**			4									56	515	88		
Bordetella bronchiseptica RB50	Proteobacteria (beta)	NC_002927	FHA	5,34	68	5070	4994	4218	**3**			3									56	593	192	24 - 48h	[57]
Bordetella parapertussis 12822	Proteobacteria (beta)	NC_002928	FHA	4,77	68	4259	4185	3654	**3**			3									54	593	165	2 - 3 days	
Bordetella pertussis Tohama I	Proteobacteria (beta)	NC_002929	FHA	4,09	68	3505	3436	2824	**3**			3									51	585	123	2 - 3 days	
Burkholderia mallei ATCC 23344	Proteobacteria (beta)	NC_006348	FL	5,84	68	5094	5025	3686	**3**		1	2								1	56	624	183	24 - 48h	[58]
Burkholderia pseudomallei 1710b	Proteobacteria (beta)	NC_007434<br>NC_007435	FL	7,31	68	6420	6347	4439	**4**			4									60	662	210	2 - 5 days	
Burkholderia pseudomallei K96243	Proteobacteria (beta)	NC_006349<br>NC_006350<br>NC_006351	FL	7,25	68	5809	5728	4468	**4**			4									61	634	218	2 - 5 days	
Burkholderia sp. 383	Proteobacteria (beta)	NC_007509<br>NC_007510<br>NC_007511	FL	8,68	66	7813	7725	6026	**6**	1		5									67	544	398	24 - 48h	
Burkholderia thailandensis E264	Proteobacteria (beta)	NC_007650<br>NC_007651	FL	6,72	68	5706	5634	4409	**4**			4									58	594	219	24 - 48h	[59]
Burkholderia xenovorans LB400	Proteobacteria (beta)	NC_007951<br>NC_007952	FL	9,73	63	9037	8951	6696	**6**			6									65	601	391	24 - 48h	
Chromobacterium violaceum ATCC 12472	Proteobacteria (beta)	NC_005085	FL	4,75	65	4547	4407	3316	**8**			8						1			98	508	136	24 - 48h	[2]
Dechloromonas aromatica RCB	Proteobacteria (beta)	NC_007298	FL	4,50	59	4283	4204	3201	**4**			4									64	436	103		
Methylobacillus flagellatus KT	Proteobacteria (beta)	NC_007947	FL	2,97	56	2822	2759	2203	**2**			2									46	685	63	3 days	[60]
Neisseria gonorrhoeae FA 1090	Proteobacteria (beta)	NC_002946	FHA	2,15	53	2069	2002	1418	**4**			4									55	591	32	48h	[2]
Neisseria meningitidis MC58	Proteobacteria (beta)	NC_003112	FHA	2,27	52	2134	2063	1536	**4**			4									59	664	28	48h	[2]

Genome Name	Phylum	Refseq	Lifestyle*	Genome Size(Mb)	GC%	Gene Count	CDS	COG Genes	operon nber	16s_23s_5s	16s_1_23s_5s	16s_2_23s_5s	16s_3_23s_5s	16s_5_23s_5s	16s_4_23s_5s	16s_23s_1_5s	additional§	5s	23s	16s	tRNA	ITS_ length (Mean)	Genes for Regulation	# Growth time	[References]
Neisseria meningitidis Z2491	Proteobacteria (beta)	NC_003116	FHA	2,18	52	2147	2065	1546	**4**			4									58	653	31	48h	[2]
Nitrosomonas europaea ATCC 19718	Proteobacteria (beta)	NC_004757	FL	2,81	51	2628	2572	1995	**1**			1						1			41	406	46	7 days	[61]
Nitrosospira multiformis ATCC 25196	Proteobacteria (beta)	NC_007614	FL	3,23	54	2876	2827	2102	**1**			1									43	657	38	5 days	[62]
Ralstonia eutropha H16	Proteobacteria (beta)	NC_008313 NC_8314	FL	6,96	67	6279	6206	5102	**5**	1		4									58	524	307	24 - 48h	[63]
Rhodoferax ferrireducens T118	Proteobacteria (beta)	NC_007908	FL	4,97	60	4555	4495	3505	**2**			2									44	625	139	5 days	[64]
Thiobacillus denitrificans ATCC 25259	Proteobacteria (beta)	NC_007404	FL	2,91	66	2879	2827	2228	**2**			2									43	803	66	4 - 5 days	[65]
Anaeromyxobacter dehalogenans 2CP-C	Proteobacteria (delta)	NC_007760	FL	5,01	75	4419	4361	3085	**2**			2									49	566	62	3 - 5 days	[66]
Bdellovibrio bacteriovorus HD100	Proteobacteria (delta)	NC_005363	FHA	3,78	51	3631	3587	2132	**2**		2										36	328	46	3 - 5 days	[67]
Desulfotalea psychrophila LSv54	Proteobacteria (delta)	NC_006138	FL	3,66	47	3321	3234	2313	**7**	5		2						1			64	443	49	3 - 5 days	[68]
Desulfovibrio vulgaris vulgaris Hildenborough	Proteobacteria (delta)	NC_002937	FL	3,77	63	3626	3531	2351	**5**			5						1			68	416	61	3 - 5 days	[69]
Geobacter metallireducens GS-15	Proteobacteria (delta)	NC_007517	FL	4,01	59	3635	3576	2721	**2**			2									50	430	78	GT 24h	[70,71]
Geobacter sulfurreducens PCA	Proteobacteria (delta)	NC_002939	FL	3,81	61	3503	3446	2527	**2**			2									49	454	76	24 - 48h	[72]
Myxococcus xanthus DK 1622	Proteobacteria (delta)	NC_008095	FL	9,14	69	7410	7331	4347	**4**		2	2									65	705	116	GT 5-6h	[73]
Pelobacter carbinolicus DSM 2380	Proteobacteria (delta)	NC_007498	FL	3,67	55	3211	3148	2482	**2**			2									54	528	60		
Syntrophus aciditrophicus SB	Proteobacteria (delta)	NC_007759	FL	3,18	51	3219	3168	2164	**1**			1									48	440	42	GT 24 - 50h	[74]
Campylobacter jejuni jejuni NCTC 11168	Proteobacteria (epsilon)	NC_002163	FHA	1,64	31	1686	1629	1294	**3**			3									44	906	14	24 - 48h	[2]
Campylobacter jejuni RM1221	Proteobacteria (epsilon)	NC_003912	FHA	1,78	30	1891	1838	1301	**3**			3									44	807	12	24 - 48h	[2]
Helicobacter acinonychis Sheeba	Proteobacteria (epsilon)	NC_008229	FHA	1,56	38	1660	1618	1078	**0**									2	2	2	36		6	3 - 5 days	[2]
Helicobacter hepaticus ATCC 51449	Proteobacteria (epsilon)	NC_004917	FHA	1,80	36	1918	1875	1249	**1**			1									37	502	14	3 - 5 days	[2]
Helicobacter pylori 26695	Proteobacteria (epsilon)	NC_000915	FHA	1,67	39	1623	1576	1104	**0**									3	2	2	36		7	3 - 5 days	[2]
Helicobacter pylori HPAG1	Proteobacteria (epsilon)	NC_008086	FHA	1,61	39	1586	1544	1094	**0**									2	2	2	36		7	3 - 5 days	[2]
Helicobacter pylori J99	Proteobacteria (epsilon)	NC_000921	FHA	1,64	39	1536	1491	1087	**0**									1	1	1	35		8	3 - 5 days	[2]
Thiomicrospira crunogena XCL-2	Proteobacteria (epsilon)	NC_007520	FL	2,43	43	2255	2200	1785	**3**			3									43	835	39	24 - 48h	[75]
Wolinella succinogenes DSM 1740	Proteobacteria (epsilon)	NC_005090	FHA	2,11	48	2096	2043	1639	**3**			3									40	560	25	24 - 48h	[2]
Acinetobacter sp. ADP1	Proteobacteria (gamma)	NC_005966	FL	3,60	40	3425	3325	2583	**7**			7									76	594	103	24 - 48h	[76]
Aeromonas hydrophila hydrophila ATCC 7966	Proteobacteria (gamma)	NC_008570	FL	4,74	62	4281	4122	3426	**10**		7	3									128	506	148	24 - 48h	[77]
Alcanivorax borkumensis SK2	Proteobacteria (gamma)	NC_008260	FL	3,12	55	2806	2755	2274	**3**	2		1									42	334	75	3 - 5 days	[78,79]
Baumannia cicadellinicola Hc	Proteobacteria (gamma)	NC_007984	M	0,69	33	641	595	584	**2**		2										39	271	6	> 7 days	[80]

Genome Name	Phylum	Refseq	Lifestyle*	Genome Size(Mb)	GC%	Gene Count	CDS	COG Genes	operon nber	16s_23s_5s	16s_1_23s_5s	16s_2_23s_5s	16s_3_23s_5s	16s_5_23s_5s	16s_4_23s_5s	16s_23s_1_5s	additional§	5s	23s	16s	tRNA	ITS_ length (Mean)	Genes for Regulation	# Growth time [References]	
Buchnera aphidicola APS	Proteobacteria (gamma)	NC_002528	M	0,66	26	610	574	561	**0**									1	1	1	32		1		
Buchnera aphidicola Bp	Proteobacteria (gamma)	NC_004545	M	0,62	25	546	507	494	**0**									1	1	1	32		2		
Buchnera aphidicola Cc	Proteobacteria (gamma)	NC_008513	M	0,42	20	394	357	350	**0**									1	1	1	31		0		
Buchnera aphidicola Sg	Proteobacteria (gamma)	NC_004061	M	0,64	25	585	546	537	**0**									1	1	1	32		2		
Candidatus Blochmannia floridanus	Proteobacteria (gamma)	NC_005061	M	0,71	27	626	583	574	**0**									1	1	1	37	307	2		
Candidatus Blochmannia pennsylvanicus BPEN	Proteobacteria (gamma)	NC_007292	M	0,79	30	654	610	599	**0**									1	1	1	39	498	3		
Candidatus Carsonella ruddii PV	Proteobacteria (gamma)	NC_008512	M	0,16	17	213	182	133	**1**	1											28	46	0		
Colwellia psychrerythraea 34H	Proteobacteria (gamma)	NC_003910	FL	5,37	38	5027	4910	3320	**9**		9							1			88	610	184	24 - 48h	[2]
Coxiella burnetii RSA 493	Proteobacteria (gamma)	NC_002971	P	2,03	43	2099	2052	1256	**1**			1									42	543	21	> 7 days	[81]
Erwinia carotovora atroseptica SCRI1043	Proteobacteria (gamma)	NC_004547	FL	5,06	51	4594	4472	3553	**7**		4	3						1			76	445	180	24 - 48h	[2]
Escherichia coli 536+A166	Proteobacteria (gamma)	NC_008253	FL	4,94	51	4732	4629	3705	**7**		6	1						1			81	441	194	24 - 48h	[2]
Escherichia coli APEC O1	Proteobacteria (gamma)	NC_008563	FL	5,08	51	4583	4467	3625	**7**	7								1			94	351	191	24 - 48h	
Escherichia coli CFT073	Proteobacteria (gamma)	NC_004431	FL	5,23	50	5562	5379	3814	**7**		5	2						1			89	355	205	24 - 48h	
Escherichia coli K12	Proteobacteria (gamma)	NC_000913	FL	4,64	51	4400	4243	3566	**7**		4	3						2			86	355	185	24 - 48h	
Escherichia coli O157:H7 EDL933	Proteobacteria (gamma)	NC_002655	FL	5,62	50	5552	5423	3990	**7**		4	3						2			98	355	198	24 - 48h	
Escherichia coli O157:H7 Sakai	Proteobacteria (gamma)	NC_002695	FL	5,59	50	5482	5341	3915	**7**		4	3						2			105	352	197	24 - 48h	
Escherichia coli UTI89	Proteobacteria (gamma)	NC_007946	FL	5,18	51	5321	5211	3792	**7**		4	3						1			88	438	197	24 - 48h	
Escherichia coli W3110	Proteobacteria (gamma)	AC_000091	FL	4,65	51	4492	4227	3565	**7**		4	3						2			91	355	185	24 - 48h	
Francisella tularensis holarctica OSU18	Proteobacteria (gamma)	NC_007880	FHA	1,90	32	1604	1555	1168	**3**			3						1			38	318	18	2 - 4 days	[82]
Francisella tularensis tularensis SCHU S4	Proteobacteria (gamma)	NC_006570	FHA	1,89	32	1651	1603	1218	**3**			3						1			38	336	20	2 - 4 days	
Haemophilus ducreyi 35000HP	Proteobacteria (gamma)	NC_002940	FHA	1,70	38	1795	1717	1256	**6**		3	3						1			47	352	24	24 - 48h	[2]
Haemophilus influenzae 86-028NP	Proteobacteria (gamma)	NC_007146	FHA	1,91	38	1868	1791	1513	**6**		3	3						1			58	478	43	24 - 48h	
Haemophilus influenzae Rd KW20	Proteobacteria (gamma)	NC_000907	FHA	1,83	38	1749	1657	1529	**6**		3	3						1			58	478	43	24 - 48h	
Hahella chejuensis KCTC 2396	Proteobacteria (gamma)	NC_007645	FL	7,22	54	6860	6778	4156	**5**			5									67	632	190	24 - 48h	[2]
Idiomarina loihiensis L2TR	Proteobacteria (gamma)	NC_006512	FL	2,84	47	2699	2628	2133	**4**			4									56	591	71	24 - 48h	[83]
Legionella pneumophila Lens	Proteobacteria (gamma)	NC_006369	FHA	3,41	38	2988	2934	2027	**3**		3										43	346	41	48 - 72h	[2]
Legionella pneumophila Paris	Proteobacteria (gamma)	NC_006368	FHA	3,64	38	3220	3166	2142	**3**		3										43	346	47	48 - 72h	
Legionella pneumophila pneumophila Philadelphia 1	Proteobacteria (gamma)	NC_002942	FHA	3,40	38	2994	2942	2040	**3**		3										43	384	46	48 - 72h	

Genome Name	Phylum	Refseq	Lifestyle*	Genome Size(Mb)	GC%	Gene Count	CDS	COG Genes	operon nber	16s_23s_5s	16s_1_23s_5s	16s_2_23s_5s	16s_3_23s_5s	16s_5_23s_5s	16s_4_23s_5s	16s_1_23s_1_5s	additional§	5s	23s	16s	tRNA	ITS_ length (Mean)	Genes for Regulation	# Growth time	[References]
Mannheimia succiniciproducens MBEL55E	Proteobacteria (gamma)	NC_006300	FHA	2,31	43	2459	2380	1882	**5**		3	2						2	1	1	60	401	68	24 - 48h	[2]
Methylococcus capsulatus Bath	Proteobacteria (gamma)	NC_002977	FL	3,30	64	3014	2960	2205	**2**			2									46	568	44	24 - 48h	[84]
Nitrosococcus oceani ATCC 19707	Proteobacteria (gamma)	NC_007484	FL	3,52	50	3186	3132	2290	**2**	1		1									45	715	51		
Pasteurella multocida multocida Pm70	Proteobacteria (gamma)	NC_002663	FHA	2,26	40	2100	2015	1819	**6**		3	3						1			57	386	52	24 - 48h	[2]
Photobacterium profundum SS9	Proteobacteria (gamma)	NC_006370 NC_006371	FL	6,40	42	5738	5491	3980	**15**	5		3	7					2			163	339	196		
Photorhabdus luminescens laumondii TTO1	Proteobacteria (gamma)	NC_005126	FL	5,69	43	4825	4683	3186	**7**		4	3						1			85	346	134	24 - 48h	[2]
Pseudoalteromonas haloplanktis TAC125	Proteobacteria (gamma)	NC_007481	FL	3,85	40	3620	3486	2639	**9**	7		2						1			106	301	99		
Pseudomonas aeruginosa PAO1	Proteobacteria (gamma)	NC_002516	FL	6,26	67	5657	5566	4592	**4**			4						1			63	472	238	24 - 48h	[2]
Pseudomonas aeruginosa UCBPP-PA14	Proteobacteria (gamma)	NC_008463	FL	6,54	66	5964	5892	4696	**4**			4									59	473	243	24 - 48h	
Pseudomonas entomophila L48	Proteobacteria (gamma)	NC_008027	FL	5,89	64	5241	5134	4018	**7**			7						1			78	513	190	24 - 48h	
Pseudomonas fluorescens Pf-5	Proteobacteria (gamma)	NC_004129	FL	7,07	63	6224	6137	4869	**5**			5									71	527	264	24 - 48h	
Pseudomonas fluorescens PfO-1	Proteobacteria (gamma)	NC_007492	FL	6,44	61	5833	5738	4533	**6**			6						1			73	510	241	24 - 48h	
Pseudomonas putida KT2440	Proteobacteria (gamma)	NC_002947	FL	6,18	62	5473	5350	4199	**7**	4		3						1			74	510	208	24 - 48h	
Pseudomonas syringae pv. phaseolicola 1448A	Proteobacteria (gamma)	NC_005773	FL	6,11	58	5250	5170	4050	**5**			5									64	553	184	24 - 48h	
Pseudomonas syringae pv. syringae B728a	Proteobacteria (gamma)	NC_007005	FL	6,09	59	5219	5136	4020	**5**			5						1			64	550	192	24 - 48h	
Pseudomonas syringae pv. tomato DC3000	Proteobacteria (gamma)	NC_004578	FL	6,54	58	5686	5608	4177	**5**			5						1			63	544	190	24 - 48h	
Psychrobacter arcticus 273-4	Proteobacteria (gamma)	NC_007204	FL	2,65	43	2211	2147	1694	**4**			4									49	594	42		
Saccharophagus degradans 2-40	Proteobacteria (gamma)	NC_007912	FL	5,06	46	4067	4017	2796	**2**	2											41	2881	98	24 - 48h	[85]
Salmonella enterica enterica sv Choleraesuis SC-B67	Proteobacteria (gamma)	NC_006905	FHA	4,94	52	4769	4662	3513	**7**		4	3						1			85	354	167	24 - 48h	[2]
Salmonella enterica enterica sv Paratyphi A ATCC 9150	Proteobacteria (gamma)	NC_006511	FHA	4,59	52	4233	4093	3320	**7**		4	3						2			82	352	153	24 - 48h	
Salmonella enterica enterica sv Typhi CT18	Proteobacteria (gamma)	NC_003198	FHA	5,13	52	4923	4758	3522	**7**		4	3						2			81	355	168	24 - 48h	
Salmonella enterica enterica sv Typhi Ty2	Proteobacteria (gamma)	NC_004631	FHA	4,79	52	4478	4318	3408	**7**		4	3						1			79	356	161	24 - 48h	
Salmonella typhimurium LT2	Proteobacteria (gamma)	NC_003197	FHA	4,95	52	4699	4527	3663	**7**		4	3						2			87	353	176	24 - 48h	
Shewanella oneidensis MR-1	Proteobacteria (gamma)	NC_004347	FL	5,13	46	4618	4472	3195	**9**	6		3						1			102	316	128	24 - 48h	[2]
Shigella boydii Sb227	Proteobacteria (gamma)	NC_007613	FHA	4,65	51	4397	4284	3470	**7**		2	5						1			91	447	133	24 - 48h	[2]
Shigella dysenteriae Sd197	Proteobacteria (gamma)	NC_007606	FHA	4,55	51	4604	4497	3725	**7**		4	3									85	438	135	24 - 48h	
Shigella flexneri 2a 2457T	Proteobacteria (gamma)	NC_004741	FHA	4,60	51	4262	4068	3457	**7**		4	3						2			100	355	135	24 - 48h	
Shigella flexneri 2a 301	Proteobacteria (gamma)	NC_004337	FHA	4,83	51	4572	4443	3637	**7**		5	1	1					2			97	355	133	24 - 48h	

Genome Name	Phylum	Refseq	Lifestyle*	Genome Size(Mb)	GC%	Gene Count	CDS	COG Genes	operon nber	16s_23s_5s	16s_1_23s_5s	16s_2_23s_5s	16s_3_23s_5s	16s_5_23s_5s	16s_4_23s_5s	16s_23s_1_5s	additional§	5s	23s	16s	tRNA	ITS_ length (Mean)	Genes for Regulation	# Growth time [References]	
Shigella flexneri 5 8401	Proteobacteria (gamma)	NC_008258	FHA	4,57	51	4235	4116	3446	**7**		7							1			97	355	134	24 - 48h	
Shigella sonnei Ss046	Proteobacteria (gamma)	NC_007384	FHA	5,04	51	4580	4461	3753	**7**		5	2						1			97	447	134	24 - 48h	
Thiomicrospira denitrificans ATCC 33889	Proteobacteria (gamma)	NC_007575	FL	2,20	34	2163	2104	1597	**4**		4										44	547	23	24 - 48h	[2]
Vibrio cholerae O1 bv eltor N16961	Proteobacteria (gamma)	NC_002505	FL	4,03	47	3989	3835	2894	**8**		3	3	1		1			1			98	427	136	24 - 48h	[2]
Vibrio fischeri ES114	Proteobacteria (gamma)	NC_006840 NC_006841	FL	4,28	38	3957	3802	3026	**12**	2	3	3	3		1			1			118	361	133	24 - 48h	
Vibrio parahaemolyticus RIMD 2210633	Proteobacteria (gamma)	NC_004603 NC_004605	FL	5,17	45	4992	4832	3529	**11**	3	1	3	2		2			1			126	358	160	24 - 48h	
Vibrio vulnificus CMCP6	Proteobacteria (gamma)	NC_004459 NC_004460	FL	5,13	47	4658	4488	3474	**9**	3	2	1	2		1			1			111	418	162	24 - 48h	
Vibrio vulnificus YJ016	Proteobacteria (gamma)	NC_005139 NC_005140	FL	5,26	47	5167	5024	3509	**9**		3	1	3		2						112	421	172	24 - 48h	
Wigglesworthia glossinidia endosymbiont of Glossina brevipalpis	Proteobacteria (gamma)	NC_004344	M	0,70	22	657	617	598	**2**		2										34	271	3	> 7 days	[86]
Yersinia pestis Antiqua	Proteobacteria (gamma)	NC_008150	FHA	4,88	48	4471	4368	3533	**7**		4	3									68	466	131	24 - 48h	[2]
Yersinia pestis biovar Microtus 91001	Proteobacteria (gamma)	NC_005810	FHA	4,80	48	4278	4142	3260	**7**		3	4						1			72	492	128	24 - 48h	
Yersinia pestis CO92	Proteobacteria (gamma)	NC_003143	FHA	4,83	48	4199	4066	3278	**6**		3	3						1			70	466	123	24 - 48h	
Yersinia pestis KIM	Proteobacteria (gamma)	NC_004088	FHA	4,70	48	4337	4202	3257	**7**		5	2						2			73	422	126	24 - 48h	
Yersinia pestis Nepal516	Proteobacteria (gamma)	NC_008149	FHA	4,65	48	4204	4097	3344	**7**		5	2									72	525	126	24 - 48h	
Yersinia pseudotuberculosis IP 32953	Proteobacteria (gamma)	NC_006155	FHA	4,84	48	4159	4038	3240	**7**		4	3									85	509	128	24 - 48h	
Borrelia burgdorferi B31	Spirochaetes	NC_001318	FHA	1,52	28	1663	1640	694	**0**									2	2	1	31		8	GT 12 - 24h	[87,88]
Borrelia garinii PBi	Spirochaetes	NC_006156	FHA	0,99	28	968	932	634	**0**									2	1	1	31		8	2 - 4 days	
Leptospira borgpetersenii sv Hardjo-bovis JB197	Spirochaetes	NC_008510	FHA	3,88	40	2920	2880	1884	**0**									1		3	37		52	21 days	[89]
Leptospira borgpetersenii sv Hardjo-bovis L550	Spirochaetes	NC_008508	FHA	3,93	40	2987	2945	1931	**0**									1	2	2	37		56	21 days	
Leptospira interrogans sv Copenhageni Fiocruz L1-130	Spirochaetes	NC_005823	FHA	4,63	35	3705	3658	2098	**0**									1	2	2	37		53	21 days	
Leptospira interrogans sv Lai 56601	Spirochaetes	NC_004342	FHA	4,69	35	4774	4727	2177	**0**									1	1	2	37		55	21 days	
Treponema denticola ATCC 35405	Spirochaetes	NC_002967	FHA	2,84	38	2824	2767	1626	**2**		2										44	385	50	3 - 5 days	[90]
Treponema pallidum pallidum Nichols	Spirochaetes	NC_000919	P	1,14	53	1088	1036	729	**2**		2										45	294	13	> 7 days	[91]
Thermotoga maritima MSB8	Thermotogae	NC_000853	FL	1,86	46	1912	1858	1553	**1**			1									46	241	48	24 - 48h	[92]

* Lifestyle: M, obligate intracellular mutualist; P, obligate intracellular parasite; FHA, facultative host-associated; FL, free-living bacteria

Grey columns represent rDNA operon organization with numbers of tRNA indicated between 16s-23s and 23s-5s; and § additional 16s, 23s and/or 5s not included in the typical operon.

Growth time corresponds to the Generation time (GT), Doubling time (DT), or Colonies (plaque essays) observation, as it has been indicated in the references.

References

1. Coute Y, Hernandez C, Appel RD, Sanchez JC, Margolles A: **Labeling of Bifidobacterium longum cells with 13C-substituted leucine for quantitative proteomic analyses.** *Appl Environ Microbiol* 2007, **73:**5653-5656.

2. **CCUG: Culture Collection, University of Göteborg, Sweden** [http://www.ccug.se/]

3. Havlir DV, Ellner JJ: ***Mycobacterium avium* complex.** In *Principles and practice of infectious diseaes*. 4th edition. Edited by Mandell GL, Douglas RG, Dolin R. New York: Churchill Livingstone; 1994:2250-2264.

4. Torres OH, Domingo P, Pericas R, Boiron P, Montiel JA, Vazquez G: **Infection caused by Nocardia farcinica: case report and review.** *Eur J Clin Microbiol Infect Dis* 2000, **19:**205-212.

5. [http://www.freepatentsonline.com/7388085.html]

6. Kelemen GH, Plaskitt KA, Lewis CG, Findlay KC, Buttner MJ: **Deletion of DNA lying close to the glkA locus induces ectopic sporulation in Streptomyces coelicolor A3(2).** *Mol Microbiol* 1995, **17:**221-230.

7. Ohno M, Okano I, Watsuji T, Kakinuma T, Ueda K, Beppu T: **Establishing the independent culture of a strictly symbiotic bacterium Symbiobacterium thermophilum from its supporting Bacillus strain.** *Biosci Biotechnol Biochem* 1999, **63:**1083-1090.

8. Fenollar F, Birg ML, Gauduchon V, Raoult D: **Culture of Tropheryma whipplei from human samples: a 3-year experience (1999 to 2002).** *J Clin Microbiol* 2003, **41:**3816-3822.

9. Deckert G, Warren PV, Gaasterland T, Young WG, Lenox AL, Graham DE, Overbeek R, Snead MA, Keller M, Aujay M et al.: **The complete genome of the hyperthermophilic bacterium Aquifex aeolicus.** *Nature* 1998, **392:**353-358.

10. Anton J, Oren A, Benlloch S, Rodriguez-Valera F, Amann R, Rossello-Mora R: **Salinibacter ruber gen. nov., sp. nov., a novel, extremely halophilic member of the Bacteria from saltern crystallizer ponds.** *Int J Syst Evol Microbiol* 2002, **52:**485-491.

11. Hackstadt T, Fischer ER, Scidmore MA, Rockey DD, Heinzen RA: **Origins and functions of the chlamydial inclusion.** *Trends Microbiol* 1997, **5:**288-293.

12. Hanada H, Ikeda-Dantsuji Y, Naito M, Nagayama A: **Infection of human fibroblast-like synovial cells with Chlamydia trachomatis results in persistent infection and interleukin-6 production.** *Microb Pathog* 2003, **34:**57-63.

13. Appino S, Pregel P, Manuali E, Vincenti L, Rota A, Carnieletto P, Tiberi C, Bollo E: **Infection of bovine oviduct cell cultures with *Chlamydophila abortus*.** *Animal Reproduction Science* 2007, **98:**350-356.

14. Ohya K, Takahara Y, Kuroda E, Koyasu S, Hagiwara S, Sakamoto M, Hisaka M, Morizane K, Ishiguro S, Yamaguchi T et al.: **Chlamydophila felis CF0218 is a novel TMH family protein with potential as a diagnostic antigen for diagnosis of C. felis infection.** *Clin Vaccine Immunol* 2008, **15:**1606-1615.

15. Tjhie JH, Roosendaal R, MacLaren DM, Vandenbroucke-Grauls CM: **Improvement of growth of Chlamydia pneumoniae on HEp-2 cells by pretreatment with polyethylene glycol in combination with additional centrifugation and extension of culture time.** *J Clin Microbiol* 1997, **35:**1883-1884.

16. **Chlorobium tepidum** [http://www.ebi.ac.uk/2can/genomes/bacteria/Chlorobium_tepidum.html]

17. Kube M, Beck A, Zinder SH, Kuhl H, Reinhardt R, Adrian L: **Genome sequence of the chlorinated compound-respiring bacterium Dehalococcoides species strain CBDB1.** *Nat Biotechnol* 2005, **23:**1269-1273.

18. Ernst A, Kirschenlohr H, Diez J, Boger P: **Glycogen content and nitrogenase activity in *Anabaena variabilis*.** *Arch Microbiol* 1984, **140:**120-125.

19. Selstam E, Campbell D: **Membrane lipid composition of the unusual cyanobacterium Gloeobacter violaceus sp. PCC 7421, which lacks sulfoquinovosyl diacylglycerol.** *Arch Microbiol* 1996, **166:**132-135.

20. Wolk CP, Cai Y, Cardemil L, Flores E, Hohn B, Murry M, Schmetterer G, Schrautemeier B, Wilson R: **Isolation and complementation of mutants of Anabaena sp. strain PCC 7120 unable to grow aerobically on dinitrogen.** *J Bacteriol* 1988, **170:**1239-1244.

21. Morris JJ, Kirkegaard R, Szul MJ, Johnson ZI, Zinser ER: **Facilitation of robust growth of Prochlorococcus colonies and dilute liquid cultures by "helper" heterotrophic bacteria.** *Appl Environ Microbiol* 2008, **74:**4530-4534.

22. Mori T, Binder B, Johnson CH: **Circadian gating of cell division in cyanobacteria growing with average doubling times of less than 24 hours.** *Proc Natl Acad Sci U S A* 1996, **93:**10183-10188.

23. Eriksson MJ, Schelin J, Miskiewicz E, Clarke AK: **Novel form of ClpB/HSP100 protein in the cyanobacterium Synechococcus.** *J Bacteriol* 2001, **183:**7392-7396.

24. Suzuki I, Sugiyami T, Omata T: **Regulation by cyanate of the genes involved in carbon and nitrogen assimilation in the cyanobacterium Synechococcus sp. strain PCC 7942.** *J Bacteriol* 1996, **178:**2688-2694.

25. Brahamsha B: **A genetic manipulation system for oceanic cyanobacteria of the genus Synechococcus.** *Appl Environ Microbiol* 1996, **62:**1747-1751.

26. Binder BJ, Chisholm SW: **Cell Cycle Regulation in Marine Synechococcus sp. Strains.** *Appl Environ Microbiol* 1995, **61:**708-717.

27. Bartsevich VV, Pakrasi HB: **Manganese transport in the cyanobacterium Synechocystis sp. PCC 6803.** *J Biol Chem* 1996, **271:**26057-26061.

28. Kirilovsky D, Roncel M, Boussac A, Wilson A, Zurita JL, Ducruet JM, Bottin H, Sugiura M, Ortega JM, Rutherford AW: **Cytochrome c550 in the cyanobacterium Thermosynechococcus elongatus: study of redox mutants.** *J Biol Chem* 2004, **279:**52869-52880.

29. Oshima T, Imahori K: **Description of Thermus thermophilus (Yoshida and Oshima) comb. nov., a nonsporulating thermophilic bacterium from a Japanese thermal spa.** *International Journal of Systematic Bacteriology* 1974,102-112.

30. Williams RA, Smith KE, Welch SG, Micallef J, Sharp RJ: **DNA relatedness of Thermus strains, description of Thermus brockianus sp. nov., and proposal to reestablish Thermus thermophilus (Oshima and Imahori).** *Int J Syst Bacteriol* 1995, **45:**495-499.

31. Bai X, Zhang J, Ewing A, Miller SA, Jancso RA, Shevchenko DV, Tsukerman K, Walunas T, Lapidus A, Campbell JW et al.: **Living with genome instability: the adaptation of phytoplasmas to diverse environments of their insect and plant hosts.** *J Bacteriol* 2006, **188:**3682-3696.

32. Wu M, Ren Q, Durkin AS, Daugherty SC, Brinkac LM, Dodson RJ, Madupu R, Sullivan SA, Kolonay JF, Haft DH et al.: **Life in hot carbon monoxide: the complete genome sequence of Carboxydothermus hydrogenoformans Z-2901.** *PLoS Genet* 2005, **1:**e65.

33. Suyama A, Iwakiri R, Kai K, Tokunaga T, Sera N, Furukawa K: **Isolation and characterization of Desulfitobacterium sp. strain Y51 capable of efficient dehalogenation of tetrachloroethene and polychloroethanes.** *Biosci Biotechnol Biochem* 2001, **65:**1474-1481.

34. Nazina TN, Tourova TP, Poltaraus AB, Novikova EV, Grigoryan AA, Ivanova AE, Lysenko AM, Petrunyaka VV, Osipov GA, Belyaev SS et al.: **Taxonomic study of aerobic thermophilic bacilli: descriptions of Geobacillus subterraneus gen. nov., sp. nov. and Geobacillus uzenensis sp. nov. from petroleum reservoirs and transfer of Bacillus stearothermophilus, Bacillus thermocatenulatus, Bacillus thermoleovorans, Bacillus kaustophilus, Bacillus thermodenitrificans to Geobacillus as the new combinations G. stearothermophilus, G. th.** *Int J Syst Evol Microbiol* 2001, **51:**433-446.

35. Rodas AM, Ferrer S, Pardo I: **Polyphasic study of wine Lactobacillus strains: taxonomic implications.** *Int J Syst Evol Microbiol* 2005, **55:**197-207.

36. Nomura M, Kimoto H, Someya Y, Suzuki I: **Novel characteristic for distinguishing Lactococcus lactis subsp. lactis from subsp. cremoris.** *Int J Syst Bacteriol* 1999, **49 Pt 1:**163-166.

37. Fraser CM, Gocayne JD, White O, Adams MD, Clayton RA, Fleischmann RD, Bult CJ, Kerlavage AR, Sutton G, Kelley JM et al.: **The minimal gene complement of Mycoplasma genitalium.** *Science* 1995, **270:**397-403.

38. Miyata M, Yamamoto H, Shimizu T, Uenoyama A, Citti C, Rosengarten R: **Gliding mutants of Mycoplasma mobile: relationships between motility and cell morphology, cell adhesion and microcolony formation.** *Microbiology* 2000, **146 (Pt 6):**1311-1320.

39. Kannan TR, Baseman JB: **Hemolytic and hemoxidative activities in Mycoplasma penetrans.** *Infect Immun* 2000, **68:**6419-6422.

40. Vaisman N, von dW, I, da Mota FF, Seldin L: **Molecular detection of Oceanobacillus iheyensis in sand of Brazilian beaches.** *J Gen Appl Microbiol* 2008, **54:**305-310.

41. Dicks LM, Dellaglio F, Collins MD: **Proposal to reclassify Leuconostoc oenos as Oenococcus oeni [corrig.] gen. nov., comb. nov.** *Int J Syst Bacteriol* 1995, **45:**395-397.

42. Xue Y, Xu Y, Liu Y, Ma Y, Zhou P: **Thermoanaerobacter tengcongensis sp. nov., a novel anaerobic, saccharolytic, thermophilic bacterium isolated from a hot spring in Tengcong, China.** *Int J Syst Evol Microbiol* 2001, **51:**1335-1341.

43. Gade D, Stuhrmann T, Reinhardt R, Rabus R: **Growth phase dependent regulation of protein composition in Rhodopirellula baltica.** *Environ Microbiol* 2005, **7:**1074-1084.

44. Munderloh UG, Lynch MJ, Herron MJ, Palmer AT, Kurtti TJ, Nelson RD, Goodman JL: **Infection of endothelial cells with Anaplasma marginale and A. phagocytophilum.** *Vet Microbiol* 2004, **101:**53-64.

45. Lin Q, Rikihisa Y, Felek S, Wang X, Massung RF, Woldehiwet Z: **Anaplasma phagocytophilum has a functional msp2 gene that is distinct from p44.** *Infect Immun* 2004, **72:**3883-3889.

46. Brenner DJ, O'Connor SP, Winkler HH, Steigerwalt AG: **Proposals to unify the genera Bartonella and Rochalimaea, with descriptions of Bartonella quintana comb. nov., Bartonella vinsonii comb. nov., Bartonella henselae comb. nov., and Bartonella elizabethae comb. nov., and to remove the family Bartonellaceae from the order Rickettsiales.** *Int J Syst Bacteriol* 1993, **43:**777-786.

47. Smit J, Hermodson M, Agabian N: **Caulobacter crescentus pilin. Purification, chemical characterization, and NH2-terminal amino acid sequence of a structural protein regulated during development.** *J Biol Chem* 1981, **256:**3092-3097.

48. Breitschwerdt EB, Hegarty BC, Hancock SI: **Sequential evaluation of dogs naturally infected with Ehrlichia canis, Ehrlichia chaffeensis, Ehrlichia equi, Ehrlichia ewingii, or Bartonella vinsonii.** *J Clin Microbiol* 1998, **36:**2645-2651.

49. Yurkov V, Stackebrandt E, Holmes A, Fuerst JA, Hugenholtz P, Golecki J, Gad'on N, Gorlenko VM, Kompantseva EI, Drews G: **Phylogenetic positions of novel aerobic, bacteriochlorophyll a-containing bacteria and description of Roseococcus thiosulfatophilus gen. nov., sp. nov., Erythromicrobium ramosum gen. nov., sp. nov., and Erythrobacter litoralis sp. nov.** *Int J Syst Bacteriol* 1994, **44:**427-434.

50. Tanaka M, Nakata Y, Mori T, Okamura Y, Miyasaka H, Takeyama H, Matsunaga T: **Development of a cell surface display system in a magnetotactic bacterium, "Magnetospirillum magneticum" AMB-1.** *Appl Environ Microbiol* 2008, **74:**3342-3348.

51. Bent SJ, Gucker CL, Oda Y, Forney LJ: **Spatial distribution of Rhodopseudomonas palustris ecotypes on a local scale.** *Appl Environ Microbiol* 2003, **69:**5192-5197.

52. Kelly PJ, Raoult D, Mason PR: **Isolation of spotted fever group rickettsias from triturated ticks using a modification of the centrifugation-shell vial technique.** *Trans R Soc Trop Med Hyg* 1991, **85:**397-398.

53. Johnson JW, Pedersen CE, Jr.: **Plaque formation by strains of spotted fever rickettsiae in monolayer cultures of various cell types.** *J Clin Microbiol* 1978, **7:**389-391.

54. Hjelm M, Riaza A, Formoso F, Melchiorsen J, Gram L: **Seasonal incidence of autochthonous antagonistic Roseobacter spp. and Vibrionaceae strains in a turbot larva (Scophthalmus maximus) rearing system.** *Appl Environ Microbiol* 2004, **70:**7288-7294.

55. Gonzalez JM, Covert JS, Whitman WB, Henriksen JR, Mayer F, Scharf B, Schmitt R, Buchan A, Fuhrman JA, Kiene RP et al.: **Silicibacter pomeroyi sp. nov. and Roseovarius nubinhibens sp. nov., dimethylsulfoniopropionate-demethylating bacteria from marine environments.** *Int J Syst Evol Microbiol* 2003, **53:**1261-1269.

56. Fenollar F, La SB, Inokuma H, Dumler JS, Taylor MJ, Raoult D: **Culture and phenotypic characterization of a Wolbachia pipientis isolate.** *J Clin Microbiol* 2003, **41:**5434-5441.

57. Goodnow RA: **Biology of Bordetella bronchiseptica.** *Microbiol Rev* 1980, **44:**722-738.

58. Ulrich MP, Norwood DA, Christensen DR, Ulrich RL: **Using real-time PCR to specifically detect Burkholderia mallei.** *J Med Microbiol* 2006, **55:**551-559.

59. Brett PJ, Deshazer D, Woods DE: **Burkholderia thailandensis sp. nov., a Burkholderia pseudomallei-like species.** *Int J Syst Bacteriol* 1998, **48 Pt 1:**317-320.

60. Urakami T, Komagata K: **Emendation of mathylobacillus Yordy and weaver 1977, a genus for methanol-utilizing bacteria.** *Int J Syst Bacteriol* 1986, **36:**502-511.

61. Hommes NG, Sayavedra-Soto LA, Arp DJ: **Mutagenesis of hydroxylamine oxidoreductase in Nitrosomonas europaea by transformation and recombination.** *J Bacteriol* 1996, **178:**3710-3714.

62. Brandt KK, Hesselsoe M, Roslev P, Henriksen K, Sorensen J: **Toxic effects of linear alkylbenzene sulfonate on metabolic activity, growth rate, and microcolony formation of Nitrosomonas and Nitrosospira strains.** *Appl Environ Microbiol* 2001, **67:**2489-2498.

63. Handrick R, Reinhardt S, Jendrossek D: **Mobilization of poly(3-hydroxybutyrate) in Ralstonia eutropha.** *J Bacteriol* 2000, **182:**5916-5918.

64. Finneran KT, Johnsen CV, Lovley DR: **Rhodoferax ferrireducens sp. nov., a psychrotolerant, facultatively anaerobic bacterium that oxidizes acetate with the reduction of Fe(III).** *Int J Syst Evol Microbiol* 2003, **53:**669-673.

65. Claus G, Kutzner HJ: **Autotrophic denitrificans by thiobacillus denitrificans in a packed bed reactor.** *Appl Microbiol Biotechnol* 1985, **22:**289-296.

66. Sanford RA, Cole JR, Tiedje JM: **Characterization and description of Anaeromyxobacter dehalogenans gen. nov., sp. nov., an aryl-halorespiring facultative anaerobic myxobacterium.** *Appl Environ Microbiol* 2002, **68:**893-900.

67. Beck S, Schwudke D, Strauch E, Appel B, Linscheid M: **Bdellovibrio bacteriovorus strains produce a novel major outer membrane protein during predacious growth in the periplasm of prey bacteria.** *J Bacteriol* 2004, **186:**2766-2773.

68. Knoblauch C, Sahm K, Jorgensen BB: **Psychrophilic sulfate-reducing bacteria isolated from permanently cold arctic marine sediments: description of Desulfofrigus oceanense gen. nov., sp. nov., Desulfofrigus fragile sp. nov., Desulfofaba gelida gen. nov., sp. nov., Desulfotalea psychrophila gen. nov., sp. nov. and Desulfotalea arctica sp. nov.** *Int J Syst Bacteriol* 1999, **49 Pt 4:**1631-1643.

69. McDougall R, Robson J, Paterson D, Tee W: **Bacteremia caused by a recently described novel Desulfovibrio species.** *J Clin Microbiol* 1997, **35:**1805-1808.

70. Lovley DR, Giovannoni SJ, White DC, Champine JE, Phillips EJ, Gorby YA, Goodwin S: **Geobacter metallireducens gen. nov. sp. nov., a microorganism capable of coupling the complete oxidation of organic compounds to the reduction of iron and other metals.** *Arch Microbiol* 1993, **159:**336-344.

71. Ortiz-Bernad I, Anderson RT, Vrionis HA, Lovley DR: **Vanadium respiration by Geobacter metallireducens: novel strategy for in situ removal of vanadium from groundwater.** *Appl Environ Microbiol* 2004, **70:**3091-3095.

72. Vinogradov E, Korenevsky A, Lovley DR, Beveridge TJ: **The structure of the core region of the lipopolysaccharide from Geobacter sulfurreducens.** *Carbohydr Res* 2004, **339:**2901-2904.

73. Burchard RP: **Growth of surface colonies of the gliding bacterium Myxococcus xanthus.** *Arch Microbiol* 1974, **96:**247-254.

74. McInerney MJ, Rohlin L, Mouttaki H, Kim U, Krupp RS, Rios-Hernandez L, Sieber J, Struchtemeyer CG, Bhattacharyya A, Campbell JW et al.: **The genome of Syntrophus aciditrophicus: life at the thermodynamic limit of microbial growth.** *Proc Natl Acad Sci U S A* 2007, **104:**7600-7605.

75. Jannasch HW, Wirsen CO, Nelson DC, Robertson LA: **Thiomicrospira crunogena sp. nov., a colorless, sulfur-oxidizing bacterium from a deep-sea hydrothermal vent.** *Int J Syst Bacteriol* 1985, **35:**422-424.

76. Barbe V, Vallenet D, Fonknechten N, Kreimeyer A, Oztas S, Labarre L, Cruveiller S, Robert C, Duprat S, Wincker P et al.: **Unique features revealed by the genome sequence of Acinetobacter sp. ADP1, a versatile and naturally transformation competent bacterium.** *Nucleic Acids Res* 2004, **32:**5766-5779.

77. Martinez-Murcia A, Monera A, Alperi A, Figueras MJ, Saavedra MJ: **Phylogenetic evidence suggests that strains of Aeromonas hydrophila subsp. dhakensis belong to the species Aeromonas aquariorum sp. nov.** *Curr Microbiol* 2009, **58:**76-80.

78. Yakimov MM, Golyshin PN, Lang S, Moore ER, Abraham WR, Lunsdorf H, Timmis KN: **Alcanivorax borkumensis gen. nov., sp. nov., a new, hydrocarbon-degrading and surfactant-producing marine bacterium.** *Int J Syst Bacteriol* 1998, **48 Pt 2:**339-348.

79. Schneiker S, Martins dS, V, Bartels D, Bekel T, Brecht M, Buhrmester J, Chernikova TN, Denaro R, Ferrer M, Gertler C et al.: **Genome sequence of the ubiquitous hydrocarbon-degrading marine bacterium Alcanivorax borkumensis.** *Nat Biotechnol* 2006, **24:**997-1004.

80. Wu D, Daugherty SC, Van Aken SE, Pai GH, Watkins KL, Khouri H, Tallon LJ, Zaborsky JM, Dunbar HE, Tran PL et al.: **Metabolic complementarity and genomics of the dual bacterial symbiosis of sharpshooters.** *PLoS Biol* 2006, **4:**e188.

81. Musso D, Raoult D: **Coxiella burnetii blood cultures from acute and chronic Q-fever patients.** *J Clin Microbiol* 1995, **33:**3129-3132.

82. Penn RL: **Francisella tuarensis (tularemia).** In *Principles and practices of infectious diseases*. 4th edition. Edited by Mandell GL, Bennett JE, Dolin R. New York: Churchill Livingstone; 1995:2061-2068.

83. Donachie SP, Hou S, Gregory TS, Malahoff A, Alam M: **Idiomarina loihiensis sp. nov., a halophilic gamma-Proteobacterium from the Lo'ihi submarine volcano, Hawai'i.** *Int J Syst Evol Microbiol* 2003, **53:**1873-1879.

84. Zahn JA, Duncan C, DiSpirito AA: **Oxidation of hydroxylamine by cytochrome P-460 of the obligate methylotroph Methylococcus capsulatus Bath.** *J Bacteriol* 1994, **176:**5879-5887.

85. Ekborg NA, Taylor LE, Longmire AG, Henrissat B, Weiner RM, Hutcheson SW: **Genomic and proteomic analyses of the agarolytic system expressed by Saccharophagus degradans 2-40.** *Appl Environ Microbiol* 2006, **72:**3396-3405.

86. Wernegreen JJ, Degnan PH, Lazarus AB, Palacios C, Bordenstein SR: **Genome evolution in an insect cell: distinct features of an ant-bacterial partnership.** *Biol Bull* 2003, **204:**221-231.

87. Norris SJ, Howell JK, Garza SA, Ferdows MS, Barbour AG: **High- and low-infectivity phenotypes of clonal populations of in vitro-cultured Borrelia burgdorferi.** *Infect Immun* 1995, **63:**2206-2212.

88. Guner ES, Hashimoto N, Kadosaka T, Imai Y, Masuzawa T: **A novel, fast-growing Borrelia sp. isolated from the hard tick Hyalomma aegyptium in Turkey.** *Microbiology* 2003, **149:**2539-2544.

89. Wuthiekanun V, Chierakul W, Limmathurotsakul D, Smythe LD, Symonds ML, Dohnt MF, Slack AT, Limpaiboon R, Suputtamongkol Y, White NJ et al.: **Optimization of culture of Leptospira from humans with leptospirosis.** *J Clin Microbiol* 2007, **45:**1363-1365.

90. Vesey PM, Kuramitsu HK: **Genetic analysis of Treponema denticola ATCC 35405 biofilm formation.** *Microbiology* 2004, **150:**2401-2407.

91. Lafond RE, Lukehart SA: **Biological basis for syphilis.** *Clin Microbiol Rev* 2006, **19:**29-49.

92. Huber R, Langworthy TA, Konig H, Thomm M, Woese CR, Sleytr UB, Stetter KO: **Thermotoga maritima sp. nov. represents a new genus of unique extremely thermophilic eubacteria growing up to 90°C.** *Arch Microbiol* 1986, **144:**324-333.
