# Supplementary figures and images for: Massive comparative genomic analysis reveals convergent evolution of specialized bacteria
Source: Biol Direct. 2009 Apr 10;4:13. doi: 10.1186/1745-6150-4-13 (PMC2688493; doi:10.1186/1745-6150-4-13)

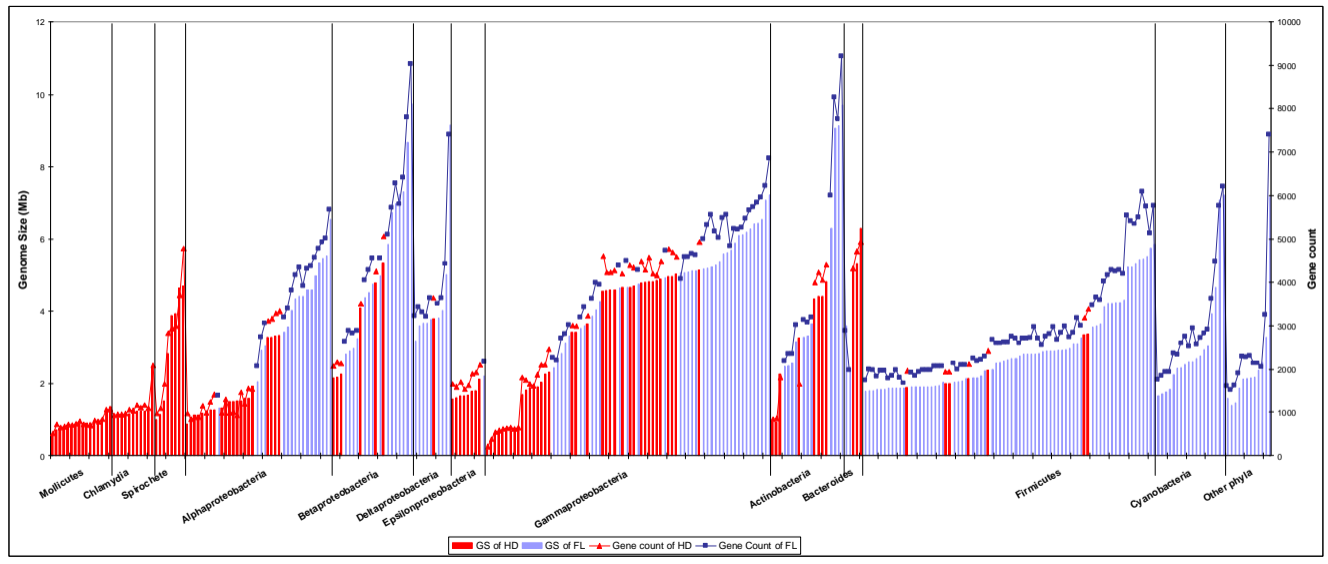

Supplement: Additional file 2 — Trends between genome size and gene count in different bacterial phyla. Columns correspond to the genome size (left axis), and the points correspond to the gene count (right axis). Red and blue colours correspond to host dependent (HD) and free-living (FL) bacteria, respectively. Other phyla: Aquifex, Thermotoga, Chlorobium, Dehalococcoides, Deinococci, Thermus, Fusobacteria, and planctomyces. Taxa are listed in Additional file 1. [file 1745-6150-4-13-S2.pdf]

## Phylogenetic relationships

## Genome content

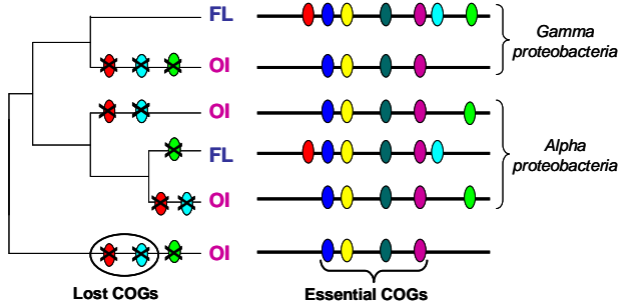

Supplement: Additional file 3 — Schematic representation of strategy used to identify essential and lost COGs. FL corresponds to free-living and OI corresponds to obligate intracellular bacteria. [file 1745-6150-4-13-S3.pdf]

**Alpha  
mutualists**

**Gamma  
mutualists**

**Alpha  
parasites**

**Gamma  
parasites**

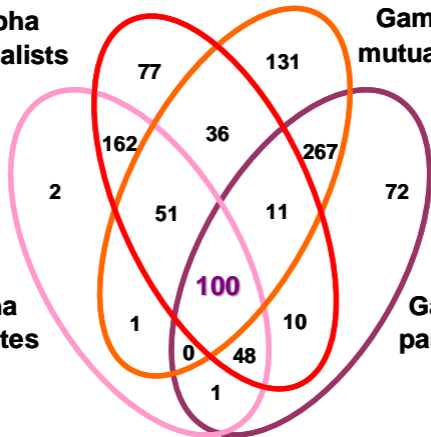

Supplement: Additional file 4 — Comparison of the sets of lost COGs. Venn diagram shows the number of shared and group-specific COGs lost in obligate intracellular bacteria and conserved in their close free-living relatives in alpha- and gamma-proteobacteria. Mycobacterium leprae lost only 63% of these COGs, however its genome content is rapidly degrading according to the high number of pseudogene [27]. [file 1745-6150-4-13-S4.pdf]

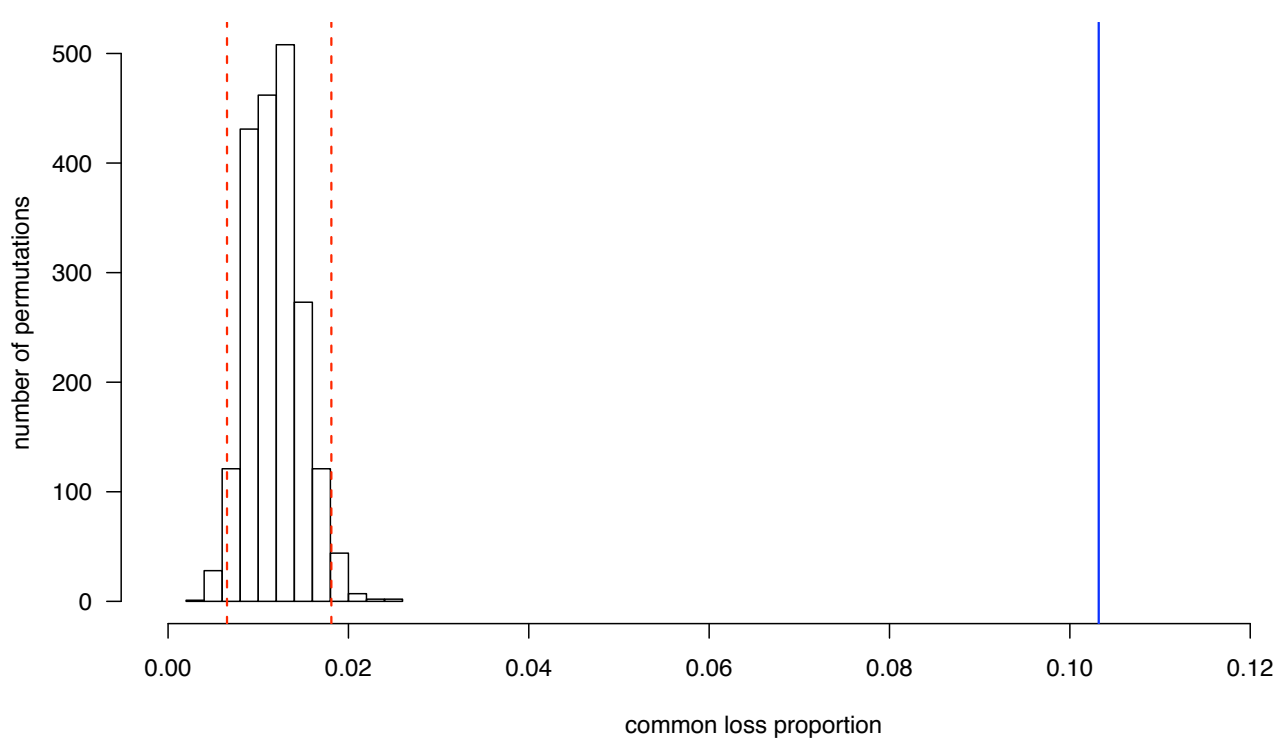

Supplement: Additional file 5 — Distribution of the common loss proportion, simulated from 2000 re-samplings. The number of COGs lost by each phylum is fixed and equals the observed loss numbers. Red dashed lines represent the 2.5% and 97.5% quantiles and blue line indicates the observed common loss proportion. 100 COGs were lost in concert among the obligate intracellular bacteria over the 969 COGs that are lost by at least one of the studied bacteria, which give a proportion of 0.1032 (100/969). The number of common COGs lost was significantly more important than expected if the loss were random (Randomization test, n = 2000, p < 10-6). [file 1745-6150-4-13-S5.pdf]

Probability of observing at least 100 COGs lost in common

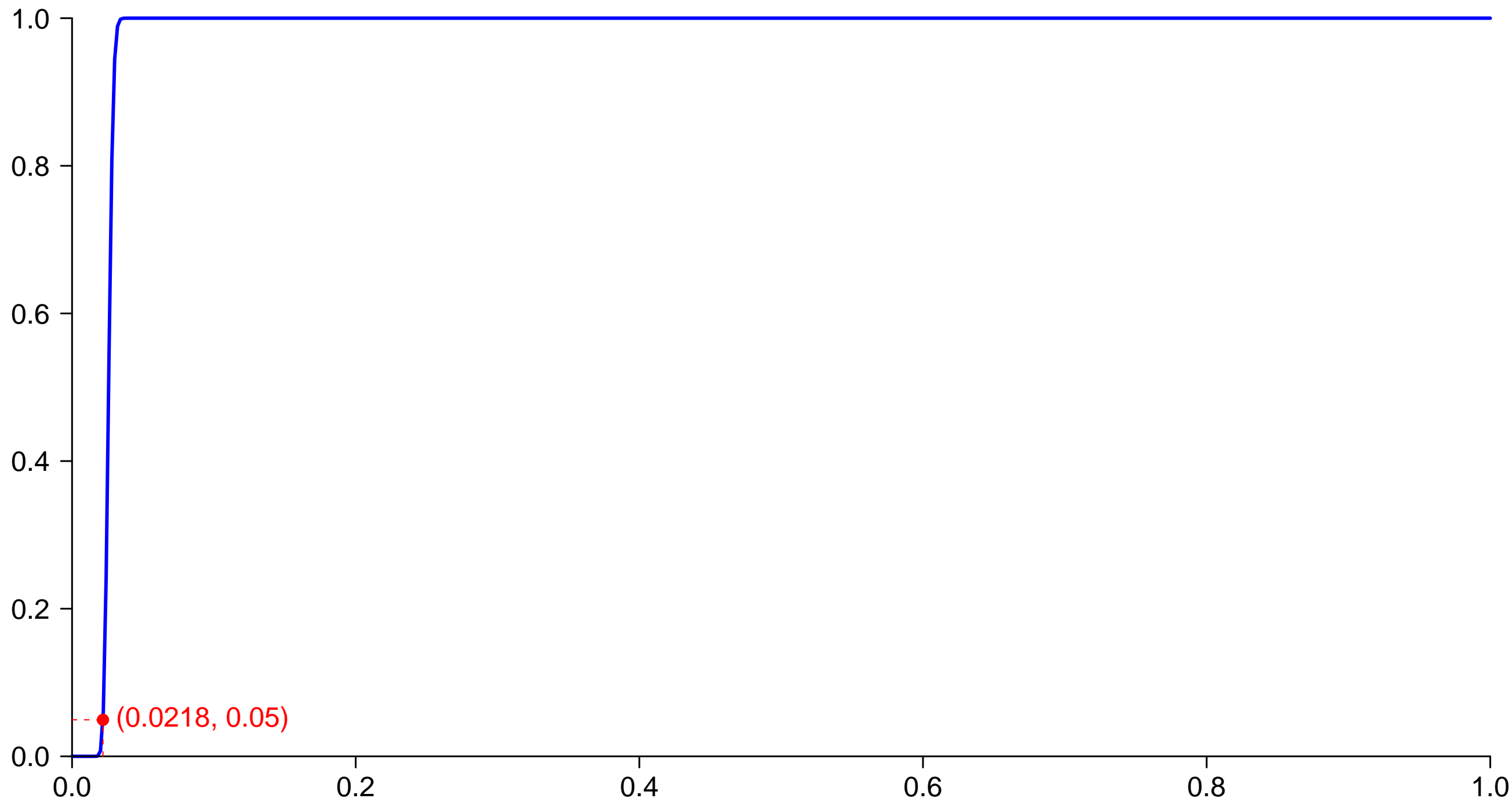

Theoretical probability  $p_0$

Supplement: Additional file 6 — Probability of losing at least 100 COGs in common Curve describing the probability of losing at least 100 COGs in common with respect to the theoretical probability of random loss (p0). The red point corresponds to the probability-threshold under which the hypothesis of independent loss between phyla is rejected (with α = 5%). [file 1745-6150-4-13-S6.pdf]

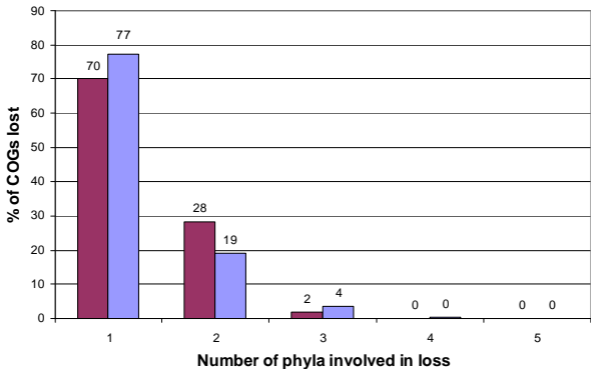

■ Among the set of 100 COGs lost in common by obligate intracellular bacteria  
■ Among all other COGs

Supplement: Additional file 10 — Distribution of COGs lost by small free-living bacteria among the set of 100 COGs lost in common by obligate intracellular bacteria and among all other COGs. [file 1745-6150-4-13-S10.pdf]

Probability of observing at least 100 COGs lost in common

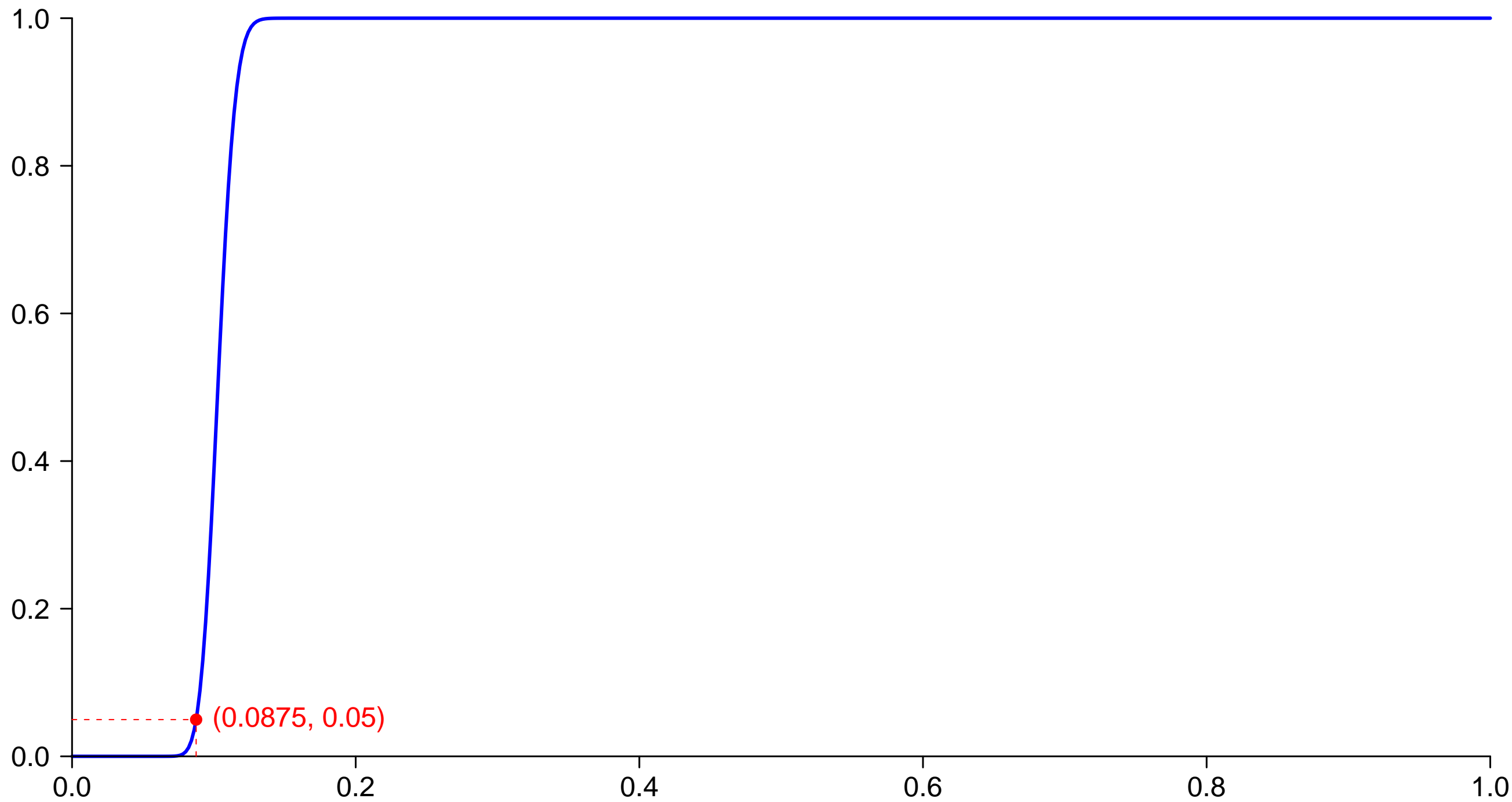

Theoretical probability  $p_0$

Supplement: Additional file 11 — Probability of losing at least 100 COGs in common among the 969 COGs lost at least by one obligate intracellular bacterium. Curve describing the probability of losing at least 100 COGs in common with respect to the theoretical probability of random loss (p0). The red point corresponds to the probability-threshold under which the hypothesis of independent loss between phyla is rejected (with α = 5%). [file 1745-6150-4-13-S11.pdf]
