# Supplementary material for: Massive comparative genomic analysis reveals convergent evolution of specialized bacteria
Source: Biol Direct. 2009 Apr 10;4:13. doi: 10.1186/1745-6150-4-13 (PMC2688493; doi:10.1186/1745-6150-4-13)
Supplement: Additional file 7 — Mean number of genes per Mb encoding for intracellular trafficking. *p-values < 0.05 are shown in bold to indicate significant differences between mutualists and parasites (Wilcoxon rank sum test). [file 1745-6150-4-13-S7.doc]

**Additional file 7- Mean number of genes per Mb encoding for intracellular trafficking (mean per Mb ± s.d.)**

|  | **Mutualists (n=13)** | **Parasites (n=27)** | **Facultative host-associated** (n=85) | **Host-Dependent** (n=125) | **Free-Living** (n=192) | ***p-*value*** |
| --- | --- | --- | --- | --- | --- | --- |
| ***Secretory pathway*** | | | | | | |
| Type II | 0.00 ± 0.00 | 1.29 ± 1.63 | 1.14 ± 1.15 | 1.05 ± 1.26 | 1.87 ± 1.61 | **0.0058** |
| Type III | 2.97 ± 3.97 | 2.72 ± 3.53 | 1.26 ± 1.60 | 1.75 ± 2.53 | 0.75 ± 0.99 | 0.9480 |
| Type IV | 1.71 ± 4.20 | 5.41 ± 5.90 | 1.24 ± 2.30 | 2.19 ± 3.94 | 0.43 ± 0.80 | **0.0485** |
| Type V | 0.00 ± 0.00 | 0.00 ± 0.00 | 0.51 ± 0.86 | 0.35 ± 0.75 | 0.07 ± 0.27 | 1.0000 |
| Type VI | 0.64 ± 0.88 | 2.07 ± 0.67 | 2.57 ± 3.75 | 2.26 ± 3.17 | 2.50 ± 2.52 | **0.0001** |
| **Other intracellular trafficking** | 18.29 ± 6.92 | 15.90 ± 3.23 | 10.82 ± 3.89 | 12.70 ± 5.00 | 9.10 ± 2.81 | **0.0453** |

*^*^p-*values < 0.05 are shown in bold to indicate significant differences between mutualists and parasites (Wilcoxon rank sum test).
