# Supplementary material for: Massive comparative genomic analysis reveals convergent evolution of specialized bacteria
Source: Biol Direct. 2009 Apr 10;4:13. doi: 10.1186/1745-6150-4-13 (PMC2688493; doi:10.1186/1745-6150-4-13)
Supplement: Additional file 8 — COGs involved in transcription. [file 1745-6150-4-13-S8.doc]

**Additional file 8-** COGs involved in transcription

| **Basal transcription factors** | | |
| --- | --- | --- |
| **COG** | **Code** | **Description** |
| COG0085 | K | DNA-directed RNA polymerase, beta subunit/140 kD subunit |
| COG0195 | K | Transcription elongation factor |
| COG0202 | K | DNA-directed RNA polymerase, alpha subunit/40 kD subunit |
| COG0317 | TK | Guanosine polyphosphate pyrophosphohydrolases/synthetases |
| COG0378 | OK | Ni2+-binding GTPase involved in regulation of expression and maturation of urease and hydrogenase |
| COG0513 | LKJ | Superfamily II DNA and RNA helicases |
| COG0515 | RTKL | Serine/threonine protein kinase |
| COG0553 | KL | Superfamily II DNA/RNA helicases, SNF2 family |
| COG0568 | K | DNA-directed RNA polymerase, sigma subunit (sigma70/sigma32) |
| COG0781 | K | Transcription termination factor |
| COG0782 | K | Transcription elongation factor |
| COG0846 | K | NAD-dependent protein deacetylases, SIR2 family |
| COG1061 | KL | DNA or RNA helicases of superfamily II |
| COG1095 | K | DNA-directed RNA polymerase, subunit E' |
| COG1158 | K | Transcription termination factor |
| COG1191 | K | DNA-directed RNA polymerase specialized sigma subunit |
| COG1199 | KL | Rad3-related DNA helicases |
| COG1200 | LK | RecG-like helicase |
| COG1224 | K | DNA helicase TIP49, TBP-interacting protein |
| COG1243 | KB | Histone acetyltransferase |
| COG1278 | K | Cold shock proteins |
| COG1293 | K | Predicted RNA-binding protein homologous to eukaryotic snRNP |
| COG1308 | K | Transcription factor homologous to NACalpha-BTF3 |
| COG1405 | K | Transcription initiation factor TFIIIB, Brf1 subunit/Transcription initiation factor TFIIB |
| COG1508 | K | DNA-directed RNA polymerase specialized sigma subunit, sigma54 homolog |
| COG1581 | K | Archaeal DNA-binding protein |
| COG1594 | K | DNA-directed RNA polymerase, subunit M/Transcription elongation factor TFIIS |
| COG1595 | K | DNA-directed RNA polymerase specialized sigma subunit, sigma24 homolog |
| COG1644 | K | DNA-directed RNA polymerase, subunit N (RpoN/RPB10) |
| COG1675 | K | Transcription initiation factor IIE, alpha subunit |
| COG1758 | K | DNA-directed RNA polymerase, subunit K/omega |
| COG1761 | K | DNA-directed RNA polymerase, subunit L |
| COG1813 | K | Predicted transcription factor, homolog of eukaryotic MBF1 |
| COG1842 | KT | Phage shock protein A (IM30), suppresses sigma54-dependent transcription |
| COG1954 | K | Glycerol-3-phosphate responsive antiterminator (mRNA-binding) |
| COG1958 | K | Small nuclear ribonucleoprotein (snRNP) homolog |
| COG1996 | K | DNA-directed RNA polymerase, subunit RPC10 (contains C4-type Zn-finger) |
| COG2012 | K | DNA-directed RNA polymerase, subunit H, RpoH/RPB5 |
| COG2093 | K | DNA-directed RNA polymerase, subunit E |
| COG2101 | K | TATA-box binding protein (TBP), component of TFIID and TFIIIB |
| COG2183 | K | Transcriptional accessory protein |
| COG2207 | K | AraC-type DNA-binding domain-containing proteins |
| COG2732 | K | Barstar, RNAse (barnase) inhibitor |
| COG2740 | K | Predicted nucleic-acid-binding protein implicated in transcription termination |
| COG2771 | K | DNA-binding HTH domain-containing proteins |
| COG2901 | KL | Factor for inversion stimulation Fis, transcriptional activator |
| COG3284 | QK | Transcriptional activator of acetoin/glycerol metabolism |
| COG3327 | K | Phenylacetic acid-responsive transcriptional repressor |
| COG3343 | K | DNA-directed RNA polymerase, delta subunit |
| COG3561 | K | Phage anti-repressor protein |
| COG3617 | K | Prophage antirepressor |
| COG3710 | K | DNA-binding winged-HTH domains |
| COG3711 | K | Transcriptional antiterminator |
| COG3933 | K | Transcriptional antiterminator |
| COG4568 | K | Transcriptional antiterminator |
| COG4578 | K | Glucitol operon activator |
| COG4646 | KL | DNA methylase |
| COG4725 | TK | Transcriptional activator, adenine-specific DNA methyltransferase |
| COG4776 | K | Exoribonuclease II |
| COG4903 | K | Genetic competence transcription factor |
| COG4936 | TK | Predicted sensor domain |
| COG4941 | K | Predicted RNA polymerase sigma factor containing a TPR repeat domain |
| COG5025 | K | Transcription factor of the Forkhead/HNF3 family |
| COG5035 | DKT | Cell cycle control protein |
| COG5108 | K | Mitochondrial DNA-directed RNA polymerase |
| COG5111 | K | DNA-directed RNA polymerase III, subunit C34 |
| COG5123 | K | Transcription initiation factor IIA, gamma subunit |
| COG5132 | KD | Cell cycle control protein, G10 family |
| COG5144 | KL | RNA polymerase II transcription initiation/nucleotide excision repair factor TFIIH, subunit TFB2 |
| COG5147 | KAD | Myb superfamily proteins, including transcription factors and mRNA splicing factors |
| COG5151 | KL | RNA polymerase II transcription initiation/nucleotide excision repair factor TFIIH, subunit SSL1 |
| COG5157 | K | RNA polymerase II assessory factor |
| COG5162 | K | Transcription initiation factor TFIID, subunit TAF10 (also component of histone acetyltransferase SAGA) |
| COG5165 | KLB | Nucleosome-binding factor SPN, POB3 subunit |
| COG5174 | K | Transcription initiation factor IIE, beta subunit |
| COG5179 | K | Transcription initiation factor TFIID, subunit TAF1 |
| COG5190 | K | TFIIF-interacting CTD phosphatases, including NLI-interacting factor |
| COG5248 | K | Transcription initiation factor TFIID, subunit TAF13 |
| COG5251 | K | Transcription initiation factor TFIID, subunit TAF11 |
| COG5624 | K | Transcription initiation factor TFIID, subunit TAF12 (also component of histone acetyltransferase SAGA) |
| COG5641 | K | GATA Zn-finger-containing transcription factor |
| COG0250 | K | Transcription antiterminator |
| COG0454 | KR | Histone acetyltransferase HPA2 and related acetyltransferases |
| COG0557 | K | Exoribonuclease R |
| COG0571 | K | dsRNA-specific ribonuclease |
| COG1197 | LK | Transcription-repair coupling factor (superfamily II helicase) |
| COG4008 | K | Predicted metal-binding transcription factor |
| COG0086 | K | DNA-directed RNA polymerase, beta' subunit/160 kD subunit |
| COG4271 | K | Predicted nucleotide-binding protein containing TIR -like domain |
| COG4512 | OTK | Membrane protein putatively involved in post-translational modification of the autoinducing quorum-sensing peptide |
| **Transcriptional regulators** | | |
| **COG** | **Code** | **Description** |
| COG0745 | TK | Response regulators consisting of a CheY-like receiver domain and a winged-helix DNA-binding domain |
| COG2002 | K | regulators of stationary/sporulation gene expression |
| COG2747 | KNU | Negative regulator of flagellin synthesis (anti-sigma28 factor) |
| COG3070 | K | regulator of competence-specific genes |
| COG3279 | KT | Response regulator of the LytR/AlgR family |
| COG3901 | K | regulator of nitric oxide reductase transcription |
| COG4219 | KT | Antirepressor regulating drug resistance, predicted signal transduction N-terminal membrane component |
| COG4565 | KT | Response regulator of citrate/malate metabolism |
| COG4567 | TK | Response regulator consisting of a CheY-like receiver domain and a Fis-type HTH domain |
| COG4650 | KT | Sigma54-dependent transcription regulator containing an AAA-type ATPase domain and a DNA-binding domain |
| COG5068 | K | regulator of arginine metabolism and related MADS box-containing transcription factors |
| COG5097 | K | RNA polymerase II transcriptional regulation mediator |
| COG2197 | TK | Response regulator containing a CheY-like receiver domain and an HTH DNA-binding domain |
| COG3835 | KT | Sugar diacid utilization regulator |
| COG2208 | TK | Serine phosphatase RsbU, regulator of sigma subunit |
| COG1974 | KT | SOS-response transcriptional repressors (RecA-mediated autopeptidases) |
| COG3160 | K | Regulator of sigma D |
| COG3437 | KT | Response regulator containing a CheY-like receiver domain and an HD-GYP domain |
| COG0583 | K | Transcriptional regulator |
| COG0640 | K | Predicted transcriptional regulators |
| COG0789 | K | Predicted transcriptional regulators |
| COG0819 | K | Putative transcription activator |
| COG0864 | K | Predicted transcriptional regulators containing the CopG/Arc/MetJ DNA-binding domain and a metal-binding domain |
| COG1167 | KE | Transcriptional regulators containing a DNA-binding HTH domain and an aminotransferase domain (MocR family) and their eukaryotic orthologs |
| COG1221 | KT | Transcriptional regulators containing an AAA-type ATPase domain and a DNA-binding domain |
| COG1316 | K | Transcriptional regulator |
| COG1318 | K | Predicted transcriptional regulators |
| COG1321 | K | Mn-dependent transcriptional regulator |
| COG1327 | K | Predicted transcriptional regulator, consists of a Zn-ribbon and ATP-cone domains |
| COG1329 | K | Transcriptional regulators, similar to M. xanthus CarD |
| COG1339 | KH | Transcriptional regulator of a riboflavin/FAD biosynthetic operon |
| COG1349 | KG | Transcriptional regulators of sugar metabolism |
| COG1378 | K | Predicted transcriptional regulators |
| COG1386 | K | Predicted transcriptional regulator containing the HTH domain |
| COG1395 | K | Predicted transcriptional regulator |
| COG1396 | K | Predicted transcriptional regulators |
| COG1414 | K | Transcriptional regulator |
| COG1420 | K | Transcriptional regulator of heat shock gene |
| COG1438 | K | Arginine repressor |
| COG1475 | K | Predicted transcriptional regulators |
| COG1476 | K | Predicted transcriptional regulators |
| COG1497 | K | Predicted transcriptional regulator |
| COG1510 | K | Predicted transcriptional regulators |
| COG1521 | K | Putative transcriptional regulator, homolog of Bvg accessory factor |
| COG1522 | K | Transcriptional regulators |
| COG1548 | KG | Predicted transcriptional regulator/sugar kinase |
| COG1609 | K | Transcriptional regulators |
| COG1678 | K | Putative transcriptional regulator |
| COG1695 | K | Predicted transcriptional regulators |
| COG1709 | K | Predicted transcriptional regulator |
| COG1725 | K | Predicted transcriptional regulators |
| COG1733 | K | Predicted transcriptional regulators |
| COG1737 | K | Transcriptional regulators |
| COG1777 | K | Predicted transcriptional regulators |
| COG1802 | K | Transcriptional regulators |
| COG1846 | K | Transcriptional regulators |
| COG1940 | KG | Transcriptional regulator/sugar kinase |
| COG1983 | KT | Putative stress-responsive transcriptional regulator |
| COG2186 | K | Transcriptional regulators |
| COG2188 | K | Transcriptional regulators |
| COG2345 | K | Predicted transcriptional regulator |
| COG2378 | K | Predicted transcriptional regulator |
| COG2390 | K | Transcriptional regulator, contains sigma factor-related N-terminal domain |
| COG2522 | K | Predicted transcriptional regulator |
| COG2808 | K | Transcriptional regulator |
| COG2865 | K | Predicted transcriptional regulator containing an HTH domain and an uncharacterized domain shared with the mammalian protein Schlafen |
| COG2909 | K | ATP-dependent transcriptional regulator |
| COG2932 | K | Predicted transcriptional regulator |
| COG2944 | K | Predicted transcriptional regulator |
| COG2973 | K | Trp operon repressor |
| COG3054 | K | Predicted transcriptional regulator |
| COG3060 | KE | Transcriptional regulator of met regulon |
| COG3311 | K | Predicted transcriptional regulator |
| COG3355 | K | Predicted transcriptional regulator |
| COG3357 | K | Predicted transcriptional regulator containing an HTH domain fused to a Zn-ribbon |
| COG3423 | K | Predicted transcriptional regulator |
| COG3432 | K | Predicted transcriptional regulator |
| COG3604 | KT | Transcriptional regulator containing GAF, AAA-type ATPase, and DNA binding domains |
| COG3609 | K | Predicted transcriptional regulators containing the CopG/Arc/MetJ DNA-binding domain |
| COG3620 | K | Predicted transcriptional regulator with C-terminal CBS domains |
| COG3636 | K | Predicted transcriptional regulator |
| COG3655 | K | Predicted transcriptional regulator |
| COG3682 | K | Predicted transcriptional regulator |
| COG3722 | K | Transcriptional regulator |
| COG3800 | K | Predicted transcriptional regulator |
| COG3829 | KT | Transcriptional regulator containing PAS, AAA-type ATPase, and DNA-binding domains |
| COG3888 | K | Predicted transcriptional regulator |
| COG3905 | K | Predicted transcriptional regulator |
| COG4109 | K | Predicted transcriptional regulator containing CBS domains |
| COG4189 | K | Predicted transcriptional regulator |
| COG4190 | K | Predicted transcriptional regulator |
| COG4465 | K | Pleiotropic transcriptional repressor |
| COG4738 | K | Predicted transcriptional regulator |
| COG4742 | K | Predicted transcriptional regulator |
| COG4800 | K | Predicted transcriptional regulator with an HTH domain |
| COG4957 | K | Predicted transcriptional regulator |
| COG4977 | K | Transcriptional regulator containing an amidase domain and an AraC-type DNA-binding HTH domain |
| COG4978 | KT | Transcriptional regulator, effector-binding domain/component |
| COG5007 | K | Predicted transcriptional regulator, BolA superfamily |
| COG5340 | K | Predicted transcriptional regulator |
| COG5450 | K | Transcription regulator of the Arc/MetJ class |
| COG5499 | K | Predicted transcription regulator containing HTH domain |
| COG5631 | K | Predicted transcription regulator, contains HTH domain (MarR family) |
| COG5662 | K | Predicted transmembrane transcriptional regulator (anti-sigma factor) |
| COG1309 | K | Transcriptional regulator |
| COG1959 | K | Predicted transcriptional regulator |
| COG2524 | K | Predicted transcriptional regulator, contains C-terminal CBS domains |
| COG3283 | KE | Transcriptional regulator of aromatic amino acids metabolism |
| COG4463 | K | Transcriptional repressor of class III stress genes |
| COG5175 | K | Transcriptional repressor |
| COG5625 | K | Predicted transcription regulator containing HTH domain |
