# Supplementary material for: Massive comparative genomic analysis reveals convergent evolution of specialized bacteria
Source: Biol Direct. 2009 Apr 10;4:13. doi: 10.1186/1745-6150-4-13 (PMC2688493; doi:10.1186/1745-6150-4-13)
Supplement: Additional file 9 — COGs involved in intracellular trafficking and secretion. [file 1745-6150-4-13-S9.doc]

**Additional file 9-** COGs involved in intracellular trafficking and secretion

| **Secretory pathways** | | | | |
| --- | --- | --- | --- | --- |
| COG2804 | NU | | Type II secretory pathway, ATPase PulE/Tfp pilus assembly pathway, ATPase PilB | |
| COG3267 | U | | Type II secretory pathway, component ExeA (predicted ATPase) | |
| COG4796 | U | | Type II secretory pathway, component HofQ | |
| COG3031 | U | | Type II secretory pathway, component PulC | |
| COG1450 | NU | | Type II secretory pathway, component PulD | |
| COG1459 | NU | | Type II secretory pathway, component PulF | |
| COG4795 | U | | Type II secretory pathway, component PulJ | |
| COG3156 | U | | Type II secretory pathway, component PulK | |
| COG3297 | U | | Type II secretory pathway, component PulL | |
| COG3149 | U | | Type II secretory pathway, component PulM | |
| COG1989 | NOU | | Type II secretory pathway, prepilin signal peptidase PulO and related peptidases | |
| COG2165 | NU | | Type II secretory pathway, pseudopilin PulG | |
| COG4790 | U | | Type III secretory pathway, component EscR | |
| COG4794 | U | | Type III secretory pathway, component EscS | |
| COG4791 | U | | Type III secretory pathway, component EscT | |
| COG4792 | U | | Type III secretory pathway, component EscU | |
| COG4789 | U | | Type III secretory pathway, component EscV | |
| COG4669 | U | | Type III secretory pathway, lipoprotein EscJ | |
| COG3736 | U | | Type IV secretory pathway, component VirB8 | |
| COG4959 | OU | | Type IV secretory pathway, protease TraF | |
| COG5268 | NU | | Type IV secretory pathway, TrbD component | |
| COG3701 | U | | Type IV secretory pathway, TrbF components | |
| COG3846 | U | | Type IV secretory pathway, TrbL components | |
| COG2948 | U | | Type IV secretory pathway, VirB10 components | |
| COG0630 | NU | | Type IV secretory pathway, VirB11 components, and related ATPases involved in archaeal flagella biosynthesis | |
| COG3838 | U | | Type IV secretory pathway, VirB2 components (pilins) | |
| COG3702 | U | | Type IV secretory pathway, VirB3 components | |
| COG3451 | U | | Type IV secretory pathway, VirB4 components | |
| COG3704 | U | | Type IV secretory pathway, VirB6 components | |
| COG3504 | U | | Type IV secretory pathway, VirB9 components | |
| COG3843 | U | | Type IV secretory pathway, VirD2 components (relaxase) | |
| COG3505 | U | | Type IV secretory pathway, VirD4 components | |
| COG3946 | U | | Type IV secretory pathway, VirJ component | |
| COG3468 | MU | | Type V secretory pathway, adhesin AidA | |
| COG0542 | O | | ATPases with chaperone activity, ATP-binding subunit | |
| COG1357 | S | | Uncharacterized low-complexity proteins | |
| COG3157 | S | | Hemolysin-coregulated protein (uncharacterized) | |
| COG3455 | S | | Uncharacterized protein conserved in bacteria | |
| COG3501 | S | | Uncharacterized protein conserved in bacteria | |
| COG3515 | S | | Uncharacterized protein conserved in bacteria | |
| COG3516 | S | | Uncharacterized protein conserved in bacteria | |
| COG3517 | S | | Uncharacterized protein conserved in bacteria | |
| COG3518 | S | | Uncharacterized protein conserved in bacteria | |
| COG3519 | S | | Uncharacterized protein conserved in bacteria | |
| COG3520 | S | | Uncharacterized protein conserved in bacteria | |
| COG3521 | S | | Uncharacterized protein conserved in bacteria | |
| COG3522 | S | | Uncharacterized protein conserved in bacteria | |
| COG3523 | S | | Uncharacterized protein conserved in bacteria | |
| COG3913 | S | | Uncharacterized protein conserved in bacteria | |
| COG4455 | R | | Protein of avirulence locus involved in temperature-dependent protein secretion | |
| **Others intracellular trafficking** | | | | |
| COG5073 | | U | | Vacuolar import and degradation protein |
| COG5295 | | UW | | Autotransporter adhesin |
| COG0848 | | U | | Biopolymer transport protein |
| COG0811 | | U | | Biopolymer transport proteins |
| COG4537 | | U | | Competence protein ComGC |
| COG4940 | | U | | Competence protein ComGF |
| COG5314 | | U | | Conjugal transfer/entry exclusion protein |
| COG5196 | | U | | ER lumen protein retaining receptor |
| COG5249 | | U | | Golgi protein involved in Golgi-to-ER retrieval |
| COG5347 | | U | | GTPase-activating protein that regulates ARFs (ADP-ribosylation factors), involved in ARF-mediated vesicular transport |
| COG3114 | | U | | Heme exporter protein D |
| COG2831 | | U | | Hemolysin activation/secretion protein |
| COG5064 | | U | | Karyopherin (importin) alpha |
| COG3210 | | U | | Large exoproteins involved in heme utilization or adhesion |
| COG0597 | | MU | | Lipoprotein signal peptidase |
| COG1585 | | OU | | Membrane protein implicated in regulation of membrane protease activity |
| COG5102 | | U | | Membrane protein involved in ER to Golgi transport |
| COG5120 | | U | | Membrane protein involved in Golgi transport |
| COG2095 | | U | | Multiple antibiotic transporter |
| COG5101 | | U | | Nuclear transport receptor CRM1/MSN5 (importin beta superfamily) |
| COG5070 | | GOU | | Nucleotide-sugar transporter |
| COG1538 | | MU | | Outer membrane protein |
| COG0823 | | U | | Periplasmic component of the Tol biopolymer transport system |
| COG0616 | | OU | | Periplasmic serine proteases (ClpP class) |
| COG5032 | | TDBLU | | Phosphatidylinositol kinase and protein kinases of the PI-3 kinase family |
| COG4473 | | U | | Predicted ABC-type exoprotein transport system, permease component |
| COG0758 | | LU | | Predicted Rossmann fold nucleotide-binding protein involved in DNA uptake |
| COG5130 | | UT | | Prenylated rab acceptor 1 and related proteins |
| **Preprotein translocase** | | | | |
| COG4023 | | U | | Preprotein translocase subunit Sec61beta |
| COG5538 | | U | | Preprotein translocase subunit Sec66 |
| COG0653 | | U | | Preprotein translocase subunit SecA (ATPase, RNA helicase) |
| COG1952 | | U | | Preprotein translocase subunit SecB |
| COG0342 | | U | | Preprotein translocase subunit SecD |
| COG0690 | | U | | Preprotein translocase subunit SecE |
| COG0341 | | U | | Preprotein translocase subunit SecF |
| COG1314 | | U | | Preprotein translocase subunit SecG |
| COG0201 | | U | | Preprotein translocase subunit SecY |
| COG2443 | | U | | Preprotein translocase subunit Sss1 |
| COG1862 | | U | | Preprotein translocase subunit YajC |
| COG0706 | | U | | Preprotein translocase subunit YidC |
| COG0740 | | OU | | Protease subunit of ATP-dependent Clp proteases |
| COG5052 | | U | | Protein involved in membrane traffic |
| COG5167 | | U | | Protein involved in vacuole import and degradation |
| COG5058 | | U | | Protein transporter of the TRAM (translocating chain-associating membrane) superfamily, longevity assurance factor |
| COG5153 | | UI | | Putative lipase essential for disintegration of autophagic bodies inside the vacuole |
| COG5080 | | U | | Rab GTPase interacting factor, Golgi membrane protein |
| COG5171 | | U | | Ran GTPase-activating protein (Ran-binding protein) |
| COG0805 | | U | | Sec-independent protein secretion pathway component TatC |
| COG1826 | | U | | Sec-independent protein secretion pathway components |
| COG0681 | | U | | Signal peptidase I |
| COG1400 | | U | | Signal recognition particle 19 kDa protein |
| COG0541 | | U | | Signal recognition particle GTPase |
| COG0552 | | U | | Signal recognition particle GTPase |
| COG5143 | | U | | Synaptobrevin/VAMP-like protein |
